# Supplementary material for: Indirect impacts of the COVID-19 pandemic at two tertiary neonatal units in Zimbabwe and Malawi: an interrupted time series analysis
Source: BMJ Open. 2022 Jun 21;12(6):e048955. doi: 10.1136/bmjopen-2021-048955 (PMC9213761; doi:10.1136/bmjopen-2021-048955)
Supplement: Supplementary data [file bmjopen-2021-048955supp001.pdf]

SUPPLEMENTARY FIGURES

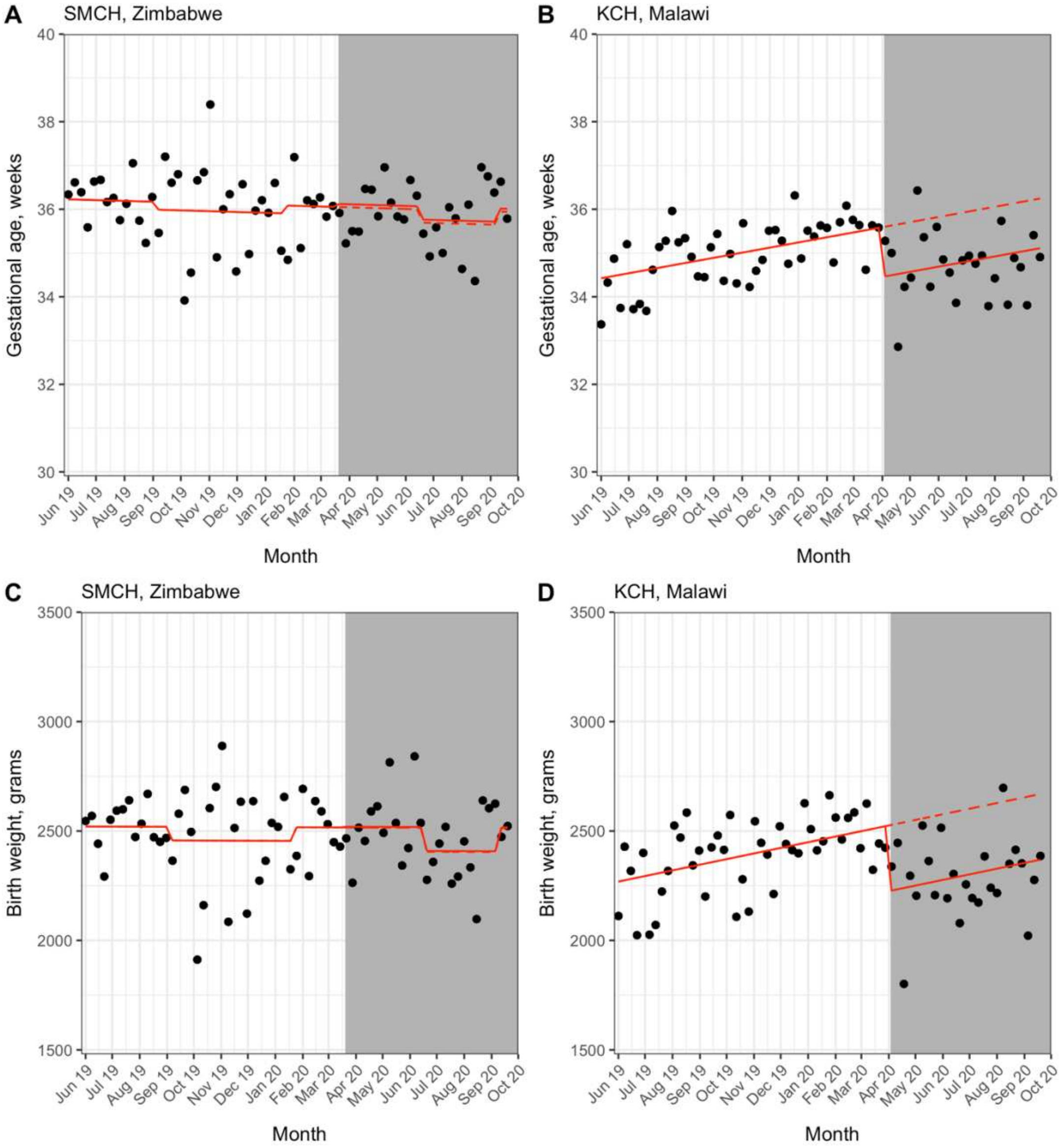

Supplementary Figure 1: Interrupted time series for gestational age and birth weight

- Data points represent weekly mean gestational age or birth weight to avoid overplotting.
- White background: pre-COVID-19 period; grey background: post-COVID-19 period.
- Solid line: predicted trend from linear regression model; dashed line: counterfactual scenario.
- SMCH models (panels A & C) adjusted for doctors' and nurses' strike periods, KCH models (panels B & D) unadjusted.
- Data from all admission forms completed, irrespective of match status.
- *SMCH: Sally Mugabe Central Hospital; KCH: Kamuzu Central Hospital*

Chimhuya S, *et al. BMJ Open* 2022; 12:e048955. doi: 10.1136/bmjopen-2021-048955

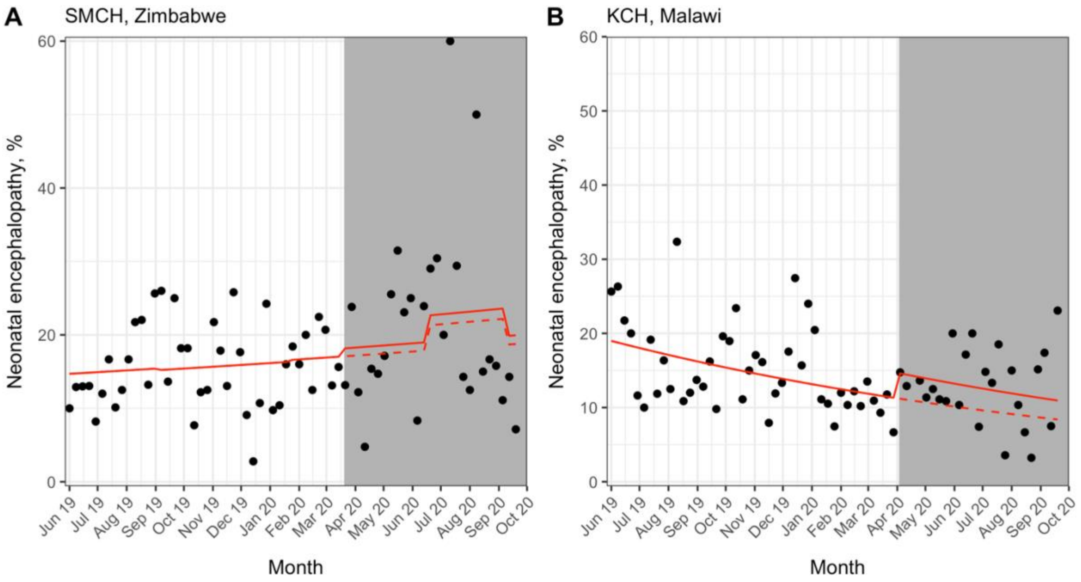

Supplementary Figure 2: Interrupted time series for prevalence of neonatal encephalopathy

- White background: pre-COVID-19 period; grey background: post-COVID-19 period.
- Solid line: predicted trend from Poisson regression model; dashed line: counterfactual scenario.
- SMCH model (panel A) adjusted for doctors' and nurses' strike periods, KCH model (panel B) unadjusted.
- Data from matched admission and outcome forms only.
- *SMCH: Sally Mugabe Central Hospital; KCH: Kamuzu Central Hospital*

APPENDIX 1: STROBE CHECKLIST

	Item No.	Recommendation	Page No.
**Title and abstract**	1	(*a*) Indicate the study's design with a commonly used term in the title or the abstract	1-2
		(*b*) Provide in the abstract an informative and balanced summary of what was done and what was found	2
**Introduction**			
Background/rationale	2	Explain the scientific background and rationale for the investigation being reported	4-5
Objectives	3	State specific objectives, including any prespecified hypotheses	5
**Methods**			
Study design	4	Present key elements of study design early in the paper	9-10
Setting	5	Describe the setting, locations, and relevant dates, including periods of recruitment, exposure, follow-up, and data collection	6-8
Participants	6	(*a*) *Cohort study*—Give the eligibility criteria, and the sources and methods of selection of participants. Describe methods of follow-up<br>*Case-control study*—Give the eligibility criteria, and the sources and methods of case ascertainment and control selection. Give the rationale for the choice of cases and controls<br>*Cross-sectional study*—Give the eligibility criteria, and the sources and methods of selection of participants	7
		(*b*) *Cohort study*—For matched studies, give matching criteria and number of exposed and unexposed<br>*Case-control study*—For matched studies, give matching criteria and the number of controls per case	n/a
Variables	7	Clearly define all outcomes, exposures, predictors, potential confounders, and effect modifiers. Give diagnostic criteria, if applicable	8
Data sources/ measurement	8*	For each variable of interest, give sources of data and details of methods of assessment (measurement). Describe comparability of assessment methods if there is more than one group	7-8
Bias	9	Describe any efforts to address potential sources of bias	9-10
Study size	10	Explain how the study size was arrived at	7
Quantitative variables	11	Explain how quantitative variables were handled in the analyses. If applicable, describe which groupings were chosen and why	9-10

Chimhuya S, *et al. BMJ Open* 2022; 12:e048955. doi: 10.1136/bmjopen-2021-048955

Statistical methods	12	(*a*) Describe all statistical methods, including those used to control for confounding	9-10
		(*b*) Describe any methods used to examine subgroups and interactions	9-10, Appendix 5
		(*c*) Explain how missing data were addressed	9-10, Appendix 4
		(*d*) *Cohort study*—If applicable, explain how loss to follow-up was addressed<br>*Case-control study*—If applicable, explain how matching of cases and controls was addressed<br>*Cross-sectional study*—If applicable, describe analytical methods taking account of sampling strategy	9
		(*e*) Describe any sensitivity analyses	9-10, Appendix 5
**Results**			
Participants	13*	(a) Report numbers of individuals at each stage of study—eg numbers potentially eligible, examined for eligibility, confirmed eligible, included in the study, completing follow-up, and analysed	12, Appendix 3
		(b) Give reasons for non-participation at each stage	9, Appendix 3
		(c) Consider use of a flow diagram	Appendix 3
Descriptive data	14*	(a) Give characteristics of study participants (eg demographic, clinical, social) and information on exposures and potential confounders	12-13, Appendix 5
		(b) Indicate number of participants with missing data for each variable of interest	Appendix 4
		(c) *Cohort study*—Summarise follow-up time (eg, average and total amount)	7
Outcome data	15*	*Cohort study*—Report numbers of outcome events or summary measures over time	12-15
		Case-control study—Report numbers in each exposure category, or summary measures of exposure	n/a
		Cross-sectional study—Report numbers of outcome events or summary measures	n/a
Main results	16	(*a*) Give unadjusted estimates and, if applicable, confounder-adjusted estimates and their precision (eg, 95% confidence interval). Make clear which confounders were adjusted for and why they were included	12-15
		(*b*) Report category boundaries when continuous variables were categorized	12-15
		(*c*) If relevant, consider translating estimates of relative risk into absolute risk for a meaningful time period	12-15

4

Other analyses	17	Report other analyses done—eg analyses of subgroups and interactions, and sensitivity analyses	Appendix 5, Appendix 6
**Discussion**			
Key results	18	Summarise key results with reference to study objectives	16
Limitations	19	Discuss limitations of the study, taking into account sources of potential bias or imprecision. Discuss both direction and magnitude of any potential bias	18-19
Interpretation	20	Give a cautious overall interpretation of results considering objectives, limitations, multiplicity of analyses, results from similar studies, and other relevant evidence	16-19
Generalisability	21	Discuss the generalisability (external validity) of the study results	16-19
**Other information**			
Funding	22	Give the source of funding and the role of the funders for the present study and, if applicable, for the original study on which the present article is based	26

Adapted from: von Elm E, Altman DG, Egger M, Pocock SJ, Gøtzsche PC, et al. (2007) The Strengthening the Reporting of Observational Studies in Epidemiology (STROBE) Statement: Guidelines for Reporting Observational Studies. PLOS Medicine 4(10): e296. https://doi.org/10.1371/journal.pmed.0040296

APPENDIX 2: ETHICAL APPROVAL

Ethical approval for this study was granted by the following ethics committees.

Table A2.1: Ethical approval

Committee	Reference
United Kingdom	
University College London Research Ethics Committee	17123/001
Malawi	
College of Medicine Research and Ethics Committee	P.01/20/2909
Zimbabwe	
Medical Research Council of Zimbabwe	MRCZ/A/2570
Joint Research Ethics Committee for the University of Zimbabwe, College of Health Sciences and Parirenyatwa Group of Hospitals	JREC/327/19
Biomedical Research and Training Institute Institutional Review Board	AP155/2020
Sally Mugabe (Harare) Central Hospital Ethics Committee	071119/64

APPENDIX 3: FLOW DIAGRAMS OF RECORD INCLUSION

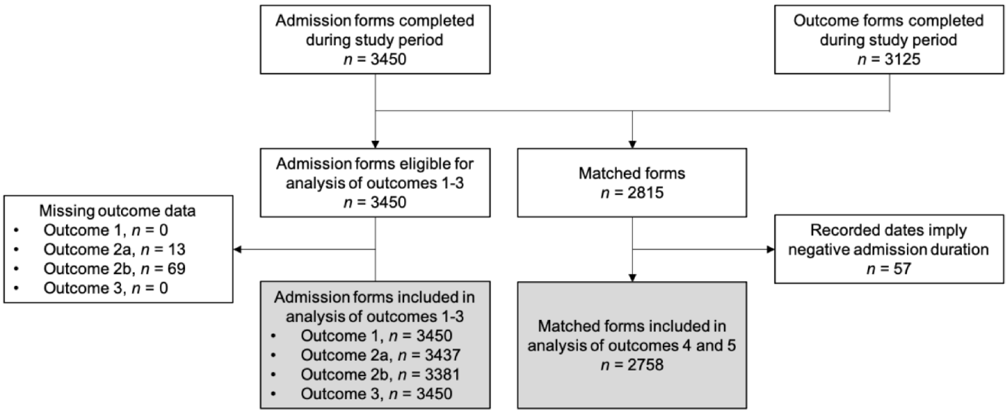

Figure A3.1: Flow diagram of record inclusion for Sally Mugabe Central Hospital, Zimbabwe

- Outcome 1: number of admissions; outcome 2a: gestational age; outcome 2b: birth weight; outcome 3: source of admission; outcome 4: prevalence of neonatal encephalopathy; outcome 5: overall mortality rate

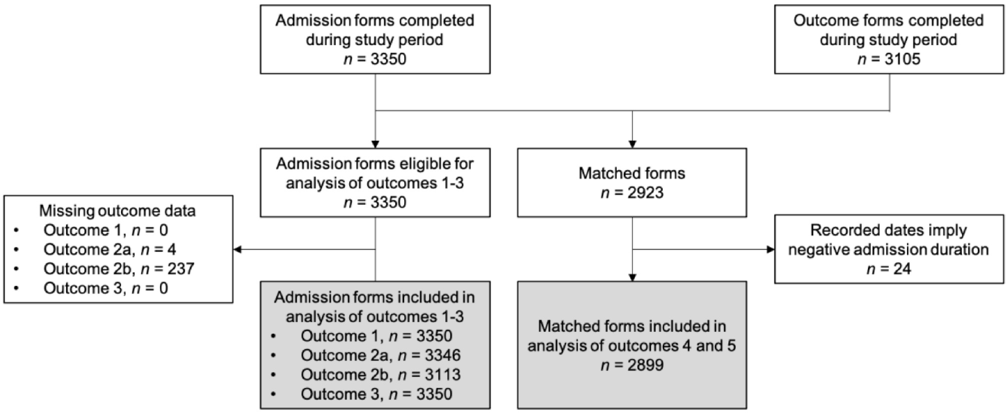

Figure A3.2: Flow diagram of record inclusion for Kamuzu Central Hospital, Malawi

- Outcome 1: number of admissions; outcome 2a: gestational age; outcome 2b: birth weight; outcome 3: source of admission; outcome 4: prevalence of neonatal encephalopathy; outcome 5: overall mortality rate

Chimhuya S, *et al. BMJ Open* 2022; 12:e048955. doi: 10.1136/bmjopen-2021-048955

APPENDIX 4: MISSING DATA

The table below shows the number of participants with missing data for each outcome and the number of participants remaining for each analysis after pairwise deletion of missing values.

Table A4.1: Summary of missing data

Characteristics	*n* missing (%)		*n* remaining*	
	SMCH	KCH	SMCH	KCH
Gestational age	13 (0·4)	4 (0·1)	3437 (99·6)	3346 (99·9)
Birth weight	69 (2·0)	237 (7·1)	3381 (98·0)	3113 (92·9)
Source of admission	0 (0·0)	0 (0·0)	3450 (100·0)	3350 (100·0)
Neonatal encephalopathy	0 (0·0)	0 (0·0)	2758 (100·0)†	2899 (100·0)†
Death	0 (0·0)	0 (0·0)	2758 (100·0)†	2899 (100·0)†

- * Remaining for analysis after pairwise deletion.
- † Only matched admission and outcome forms considered for analysis of neonatal encephalopathy and death.
- *SMCH: Sally Mugabe Central Hospital; KCH: Kamuzu Central Hospital, Malawi*

Chimhuya S, *et al. BMJ Open* 2022; 12:e048955. doi: 10.1136/bmjopen-2021-048955

APPENDIX 5: FURTHER REGRESSION ANALYSIS RESULTS

Outcome 1: Admissions to the neonatal unit

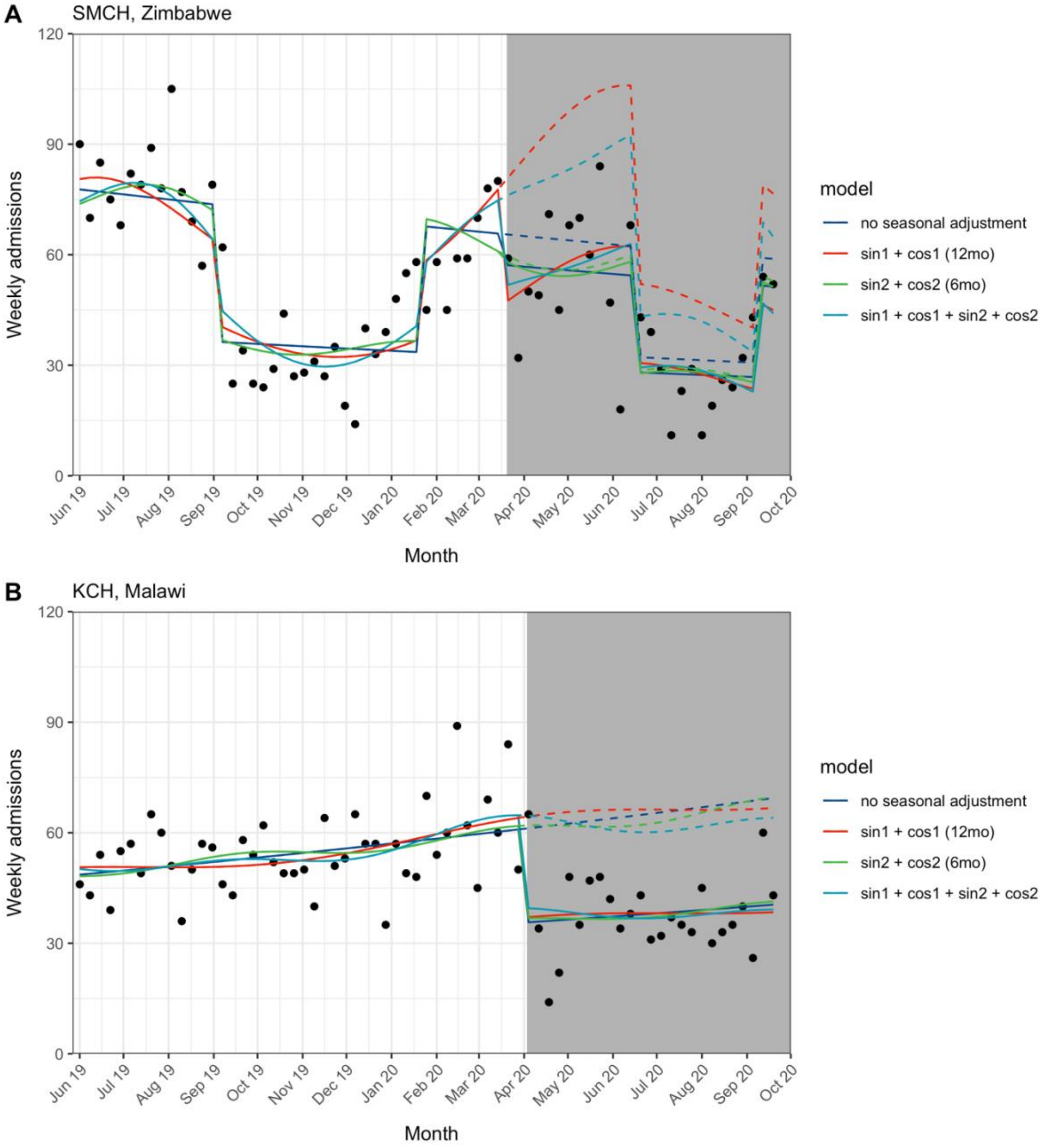

Figure A5.1.1: Interrupted time series for weekly admissions to the neonatal unit, negative binomial regression models with and without seasonal adjustment

Table A5.1.1: SMCH, Zimbabwe; Results of the models with and without adjustment for seasonality

Model*		BIC	LR statistic†	Df	*p*-value
0	Negative binomial, unadjusted for seasonality	585·6	ref		
1	Negative binomial, cosine function with 6-month period	588·9	5·23	2	0·07
2	Negative binomial, cosine function with 12-month period	592·9	1·22	2	0·54
3	Negative binomial, mixture of two cosine functions with 6-month and 12-month periods	595·6	6·96	4	0·13

- * All models adjusted for the doctors' and nurses' strike periods.
- † Likelihood ratio χ^2-test compared to Model 0.

Table A5.1.2: SMCH, Zimbabwe; Negative binomial model, unadjusted for seasonality (Model 0)

	Coef	SE	Exp	95% CI	*p*-value
Intercept	*4·35*	*0·09*			
Post-COVID-19 period, yes	-0·14	0·15	0·87	0·65 – 1·17	0·37
Study time elapsed, weeks	-0·00	0·00	1·00	0·99 – 1·00	0·25
Doctors' strike period, yes	-0·70	0·10	0·49	0·41 – 0·60	< 0·001
Nurses' strike period, yes	-0·66	0·13	0·52	0·41 – 0·66	< 0·001

Table A5.1.3: KCH, Malawi; Results of the models with and without adjustment for seasonality

Model		BIC	LR statistic†	Df	*p*-value
0	Negative binomial, unadjusted for seasonality	534·5	ref		
1	Negative binomial, cosine function with 6-month period	541·5	1·40	2	0·50
2	Negative binomial, cosine function with 12-month period	542·4	0·52	2	0·77
3	Negative binomial, mixture of two cosine functions with 6-month and 12-month periods	549·1	2·36	4	0·67

- † Likelihood ratio χ^2-test compared to Model 0.

Table A5.1.4: KCH, Malawi; Negative binomial model, unadjusted for seasonality (Model 0)

	Coef	SE	Exp	95% CI	*p*-value
Intercept	*3·88*	*0·06*			
Post-COVID-19 period, yes	-0·54	0·10	0·58	0·48 – 0·70	< 0·001
Study time elapsed, weeks	0·01	0·00	1·01	1·00 – 1·01	0·022

Chimhuya S, *et al. BMJ Open* 2022; 12:e048955. doi: 10.1136/bmjopen-2021-048955

Outcome 2: Gestational age at birth and birth weight

a. Gestational age at birth

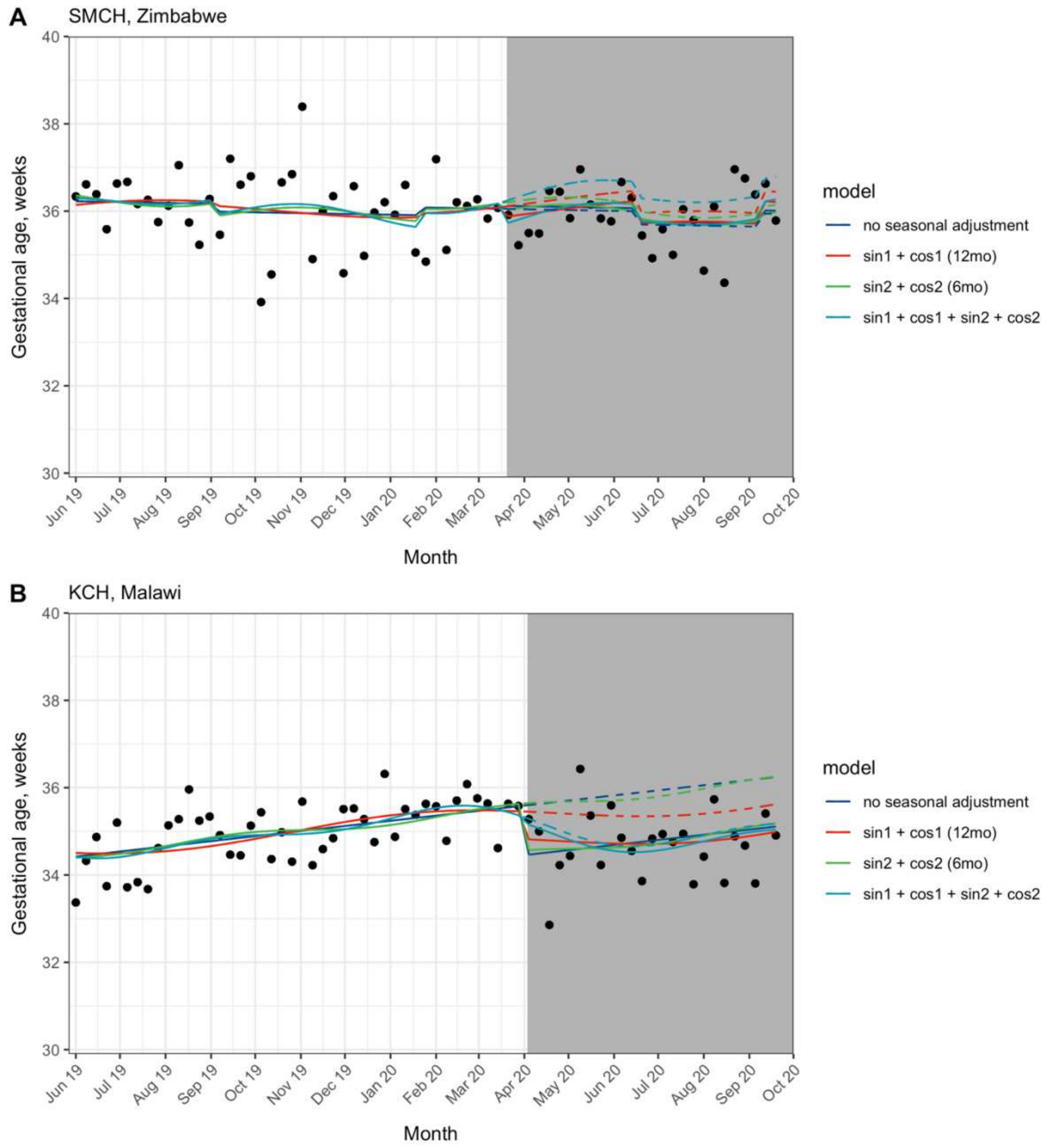

Figure A5.2.1: Interrupted time series for gestational age at birth, linear regression models with and without seasonal adjustment

Table A5.2.1: SMCH, Zimbabwe; Results of the models with and without adjustment for seasonality

Model*		BIC	Deviance†	Df	*p*-value
0	Linear, unadjusted for seasonality	19851·6	ref		
1	Linear, cosine function with 6-month period	19866·6	24·0	2	0·53
2	Linear, cosine function with 12-month period	19867·0	15·8	2	0·65
3	Linear, mixture of two cosine functions with 6-month and 12-month periods	19881·4	50·9	4	0·60

- * All models adjusted for the doctors' and nurses' strike periods.
- † χ^2-test compared to Model 0.

Table A5.2.2: SMCH, Zimbabwe; Linear model, unadjusted for seasonality (Model 0)

	Coef	SE	95% CI	*p*-value
Intercept	*36·23*	*0·15*		
Post-COVID-19 period, yes	0·07	0·29	-0·50 – 0·64	0·81
Study time elapsed, weeks	-0·00	0·01	-0·02 – 0·01	0·52
Doctors' strike period, yes	-0·18	0·20	-0·58 – 0·22	0·38
Nurses' strike period, yes	-0·30	0·29	-0·87 – 0·27	0·30

Table A5.2.3: KCH, Malawi; Results of the models with and without adjustment for seasonality

Model		BIC	Deviance†	Df	*p*-value
0	Linear, unadjusted for seasonality	18631·8	ref		
1	Linear, cosine function with 6-month period	18645·2	43·2	2	0·24
2	Linear, cosine function with 12-month period	18647·2	12·9	2	0·65
3	Linear, mixture of two cosine functions with 6-month and 12-month periods	18658·4	89·0	4	0·21

- † χ^2-test compared to Model 0.

Table A5.2.4: KCH, Malawi; Linear model, unadjusted for seasonality (Model 0)

	Coef	SE	95% CI	*p*-value
Intercept	*34·42*	*0·15*		
Post-COVID-19 period, yes	-1·14	0·25	-1·62 – -0·65	< 0·001
Study time elapsed, weeks	0·03	0·01	0·02 – 0·04	< 0·001

Chimhuya S, *et al. BMJ Open* 2022; 12:e048955. doi: 10.1136/bmjopen-2021-048955

b. Birth weight

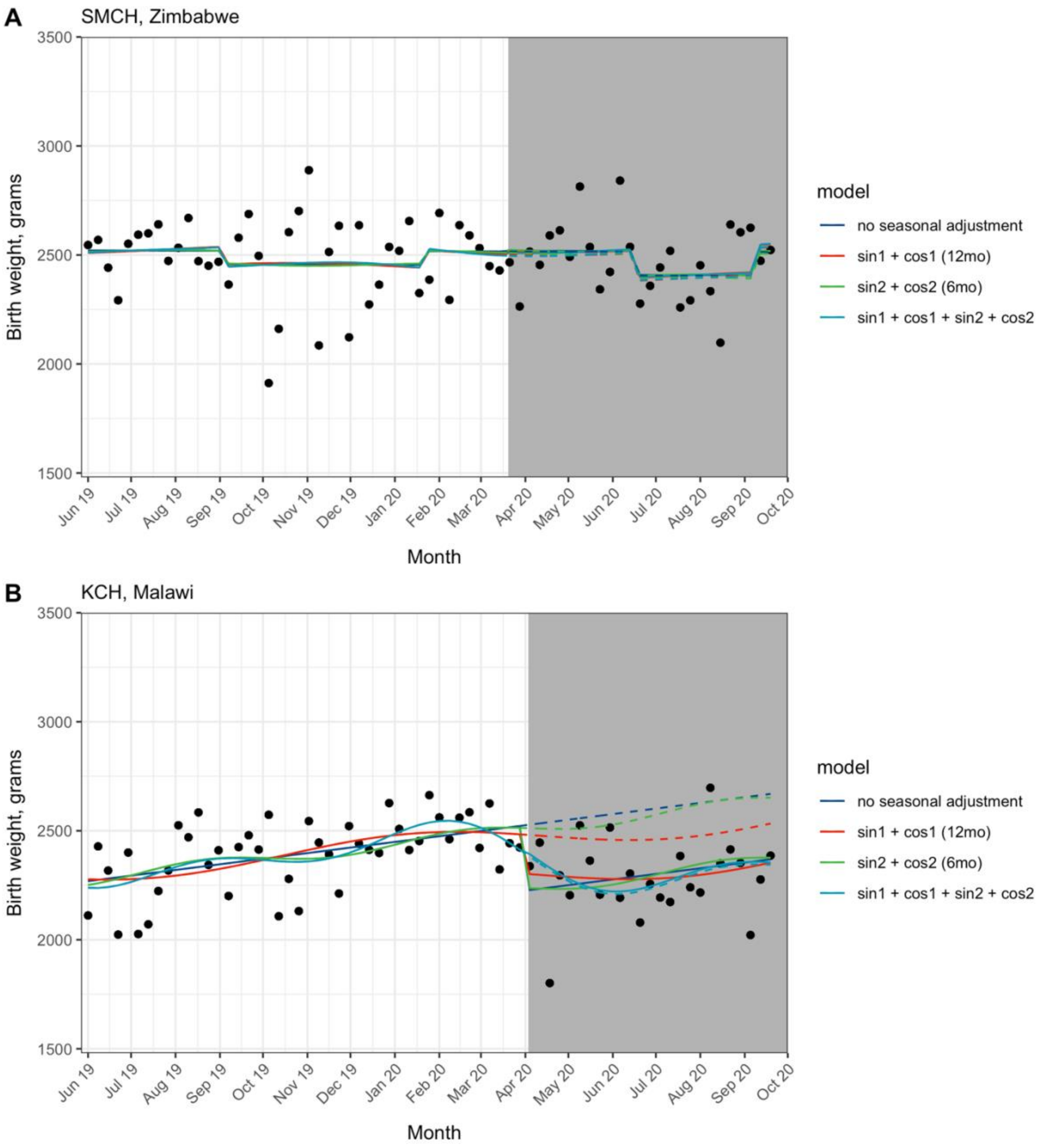

Figure A5.2.2: Interrupted time series for birth weight, linear regression models with and without seasonal adjustment

Table A5.2.5: SMCH, Zimbabwe; Results of the models with and without adjustment for seasonality

Model*		BIC	Deviance†	Df	*p*-value
0	Linear, unadjusted for seasonality	55660·9	ref		
1	Linear, cosine function with 6-month period	55676·8	289194	2	0·84
2	Linear, cosine function with 12-month period	55677·1	28641	2	0·98
3	Linear, mixture of two cosine functions with 6-month and 12-month periods	55693·0	351647	4	0·98

- * All models adjusted for the doctors' and nurses' strike periods.
- † χ^2-test compared to Model 0.

Table A5.2.6: SMCH, Zimbabwe; Linear model, unadjusted for seasonality (Model 0)

	Coef	SE	95% CI	*p*-value
Intercept	*2520·71*	*31·89*		
Post-COVID-19 period, yes	3·38	61·42	-117·0 – 123·8	0·96
Study time elapsed, weeks	-0·11	1·38	-2·8 – 2·6	0·94
Doctors' strike period, yes	-62·52	42·92	-146·6 – 21·6	0·15
Nurses' strike period, yes	-109·4	61·0	-229·0 – 10·2	0·07

Table A5.2.7: KCH, Malawi; Results of the models with and without adjustment for seasonality

Model		BIC	Deviance†	Df	*p*-value
0	Linear, unadjusted for seasonality	51050·5	ref		
1	Linear, cosine function with 6-month period	51064·1	1922568	2	0·29
2	Linear, cosine function with 12-month period	51065·2	1105739	2	0·49
3	Linear, mixture of two cosine functions with 6-month and 12-month periods	51073·9	6744491	4	0·07

- † χ^2-test compared to Model 0.

Table A5.2.8: KCH, Malawi; Linear model, unadjusted for seasonality (Model 0)

	Coef	SE	95% CI	*p*-value
Intercept	*2268·96*	*36·02*		
Post-COVID-19 period, yes	-299·89	57·34	-412·3 – -187·5	< 0·001
Study time elapsed, weeks	5·88	1·37	3·2 – 8·6	< 0·001

Chimhuya S, *et al. BMJ Open* 2022; 12:e048955. doi: 10.1136/bmjopen-2021-048955

Outcome 3: Source of admission referral

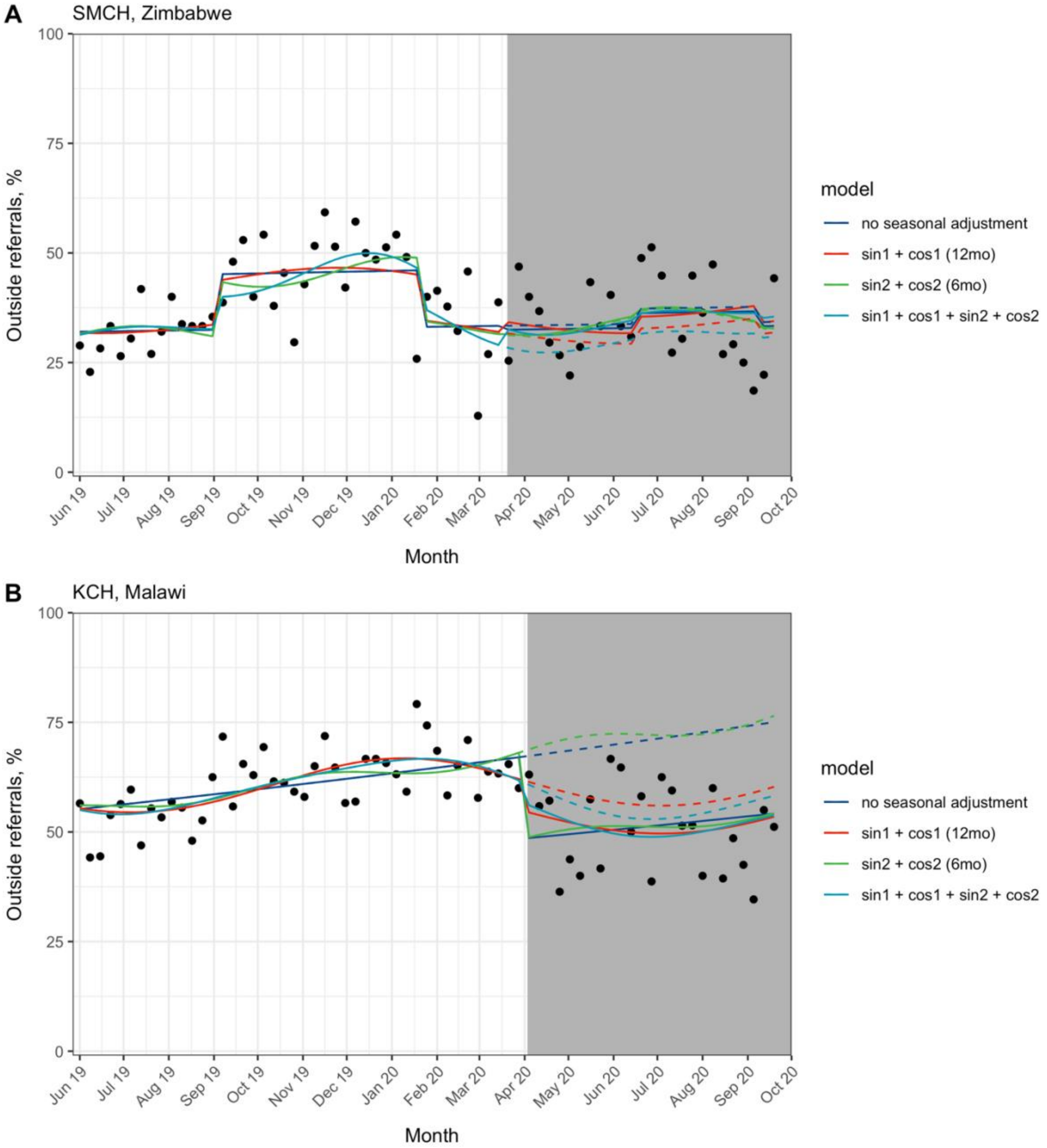

Figure A5.3.1: Interrupted time series for outside referrals to the neonatal unit, Poisson regression models with and without seasonal adjustment

Chimhuya S, *et al. BMJ Open* 2022; 12:e048955. doi: 10.1136/bmjopen-2021-048955

Table A5.3.1: SMCH, Zimbabwe; Results of the models with and without adjustment for seasonality

Model*		BIC	Deviance†	Df	*p*-value
0	Poisson, unadjusted for seasonality	406·3	ref		
1	Poisson, cosine function with 6-month period	414·2	0·56	2	0·76
2	Poisson, cosine function with 12-month period	412·9	1·85	2	0·40
3	Poisson, mixture of two cosine functions with 6-month and 12-month periods	419·8	3·42	4	0·49

- * All models adjusted for the doctors' and nurses' strike periods.
- † χ^2-test compared to Model 0.

Table A5.3.2: SMCH, Zimbabwe; Poisson model, unadjusted for seasonality (Model 0)

	Coef	SE	Exp	95% CI	*p*-value
Intercept	*-1·14*	*0·06*			
Post-COVID-19 period, yes	-0·03	0·12	0·97	0·77 – 1·22	0·81
Study time elapsed, weeks	0·00	0·00	1·00	1·00 – 1·01	0·70
Doctors' strike period, yes	0·33	0·07	1·39	1·20 – 1·61	< 0·001
Nurses' strike period, yes	0·10	0·11	1·10	0·88 – 1·37	0·39

Table A5.3.3: KCH, Malawi; Results of the models with and without adjustment for seasonality

Model		BIC	Deviance†	Df	*p*-value
0	Poisson, unadjusted for seasonality	398·0	ref		
1	Poisson, cosine function with 6-month period	403·3	3·23	2	0·20
2	Poisson, cosine function with 12-month period	405·9	0·58	2	0·75
3	Poisson, mixture of two cosine functions with 6-month and 12-month periods	411·5	3·43	4	0·49

- † χ^2-test compared to Model 0.

Table A5.3.4: KCH, Malawi; Poisson model, unadjusted for seasonality (Model 0)

	Coef	SE	Exp	95% CI	*p*-value
Intercept	*-0·59*	*0·05*			
Post-COVID-19 period, yes	-0·33	0·08	0·72	0·61 – 0·85	< 0·001
Study time elapsed, weeks	0·01	0·00	1·01	1·00 – 1·01	0·020

Chimhuya S, *et al. BMJ Open* 2022; 12:e048955. doi: 10.1136/bmjopen-2021-048955

Outcome 4: Prevalence of neonatal encephalopathy

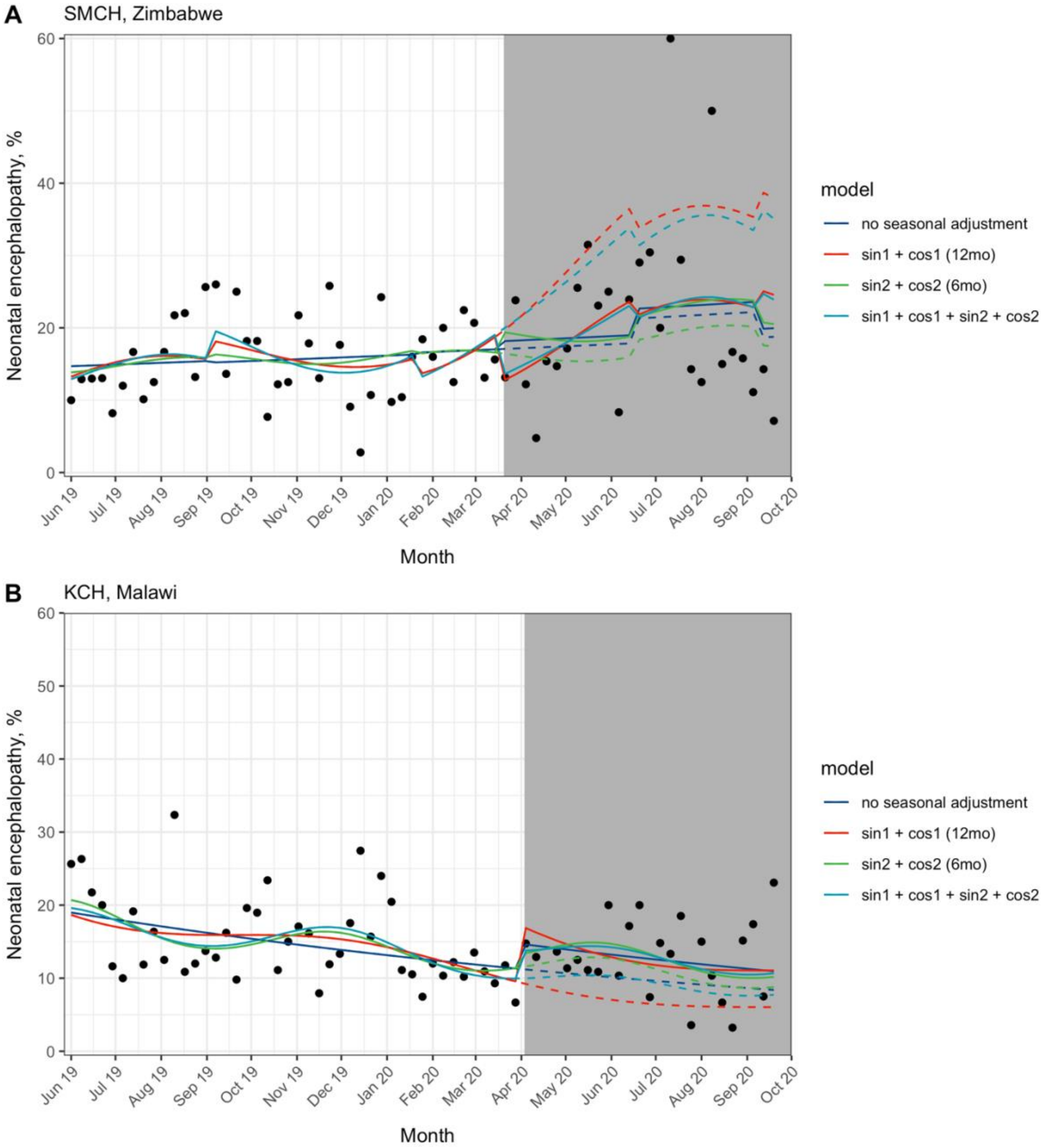

Figure A5.4.1: Interrupted time series for prevalence of neonatal encephalopathy, Poisson regression models with and without seasonal adjustment

Table A5.4.1: SMCH, Zimbabwe; Results of the models with and without adjustment for seasonality

Model*		BIC	Deviance†	Df	*p*-value
0	Poisson, unadjusted for seasonality	333·5	ref		
1	Poisson, cosine function with 6-month period	336·9	5·06	2	0·08
2	Poisson, cosine function with 12-month period	341·5	0·45	2	0·80
3	Poisson, mixture of two cosine functions with 6-month and 12-month periods	345·0	5·39	4	0·25

- * All models adjusted for the doctors' and nurses' strike periods.
- † χ^2-test compared to Model 0.

Table A5.4.2: SMCH, Zimbabwe; Poisson model, unadjusted for seasonality (Model 0)

	Coef	SE	Exp	95% CI	*p*-value
Intercept	*-1·92*	*0·10*			
Post-COVID-19 period, yes	0·06	0·18	1·06	0·74 – 1·52	0·74
Study time elapsed, weeks	0·00	0·00	1·00	1·00 – 1·01	0·39
Doctors' strike period, yes	-0·02	0·13	0·99	0·77 – 1·26	0·91
Nurses' strike period, yes	0·18	0·18	1·19	0·84 – 1·69	0·33

Table A5.4.3: KCH, Malawi; Results of the models with and without adjustment for seasonality

Model		BIC	Deviance†	Df	*p*-value
0	Poisson, unadjusted for seasonality	302·3	ref		
1	Poisson, cosine function with 6-month period	308·9	1·83	2	0·40
2	Poisson, cosine function with 12-month period	307·5	3·29	2	0·19
3	Poisson, mixture of two cosine functions with 6-month and 12-month periods	315·3	3·92	4	0·42

- † χ^2-test compared to Model 0.

Table A5.4.4: KCH, Malawi; Poisson model, unadjusted for seasonality (Model 0)

	Coef	SE	Exp	95% CI	*p*-value
Intercept	*-1·66*	*0·10*			
Post-COVID-19 period, yes	0·27	0·19	1·31	0·91 – 1·88	0·15
Study time elapsed, weeks	-0·01	0·00	0·99	0·99 – 1·00	0·005

Chimhuya S, *et al. BMJ Open* 2022; 12:e048955. doi: 10.1136/bmjopen-2021-048955

Outcome 5: Overall mortality

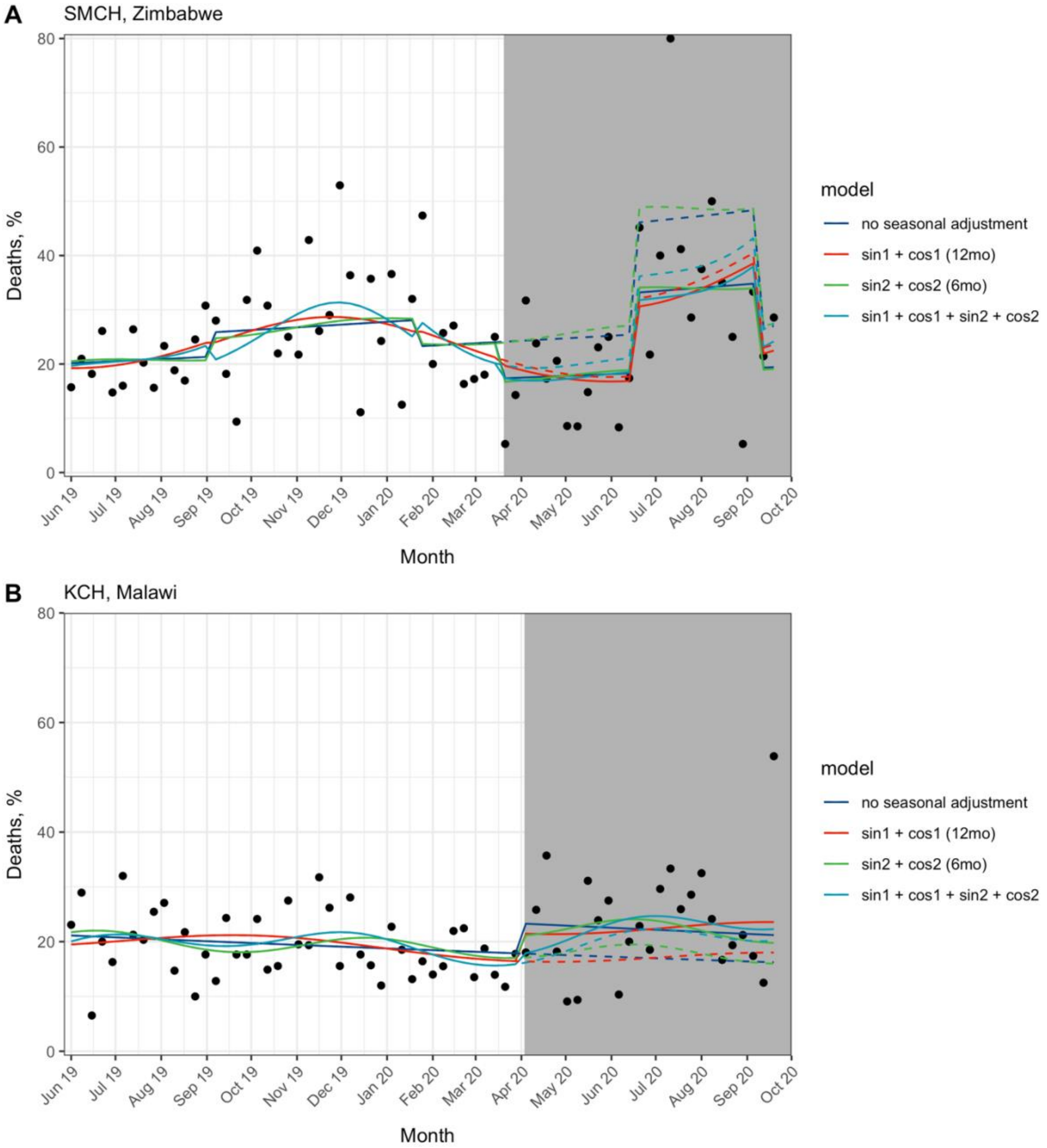

Figure A5.5.1: Interrupted time series for overall mortality, negative binomial regression models (SMCH, Zimbabwe) and Poisson regression models (KCH, Malawi) with and without seasonal adjustment

Table A5.5.1: SMCH, Zimbabwe; Results of the models with and without adjustment for seasonality

Model*		BIC	LR statistic†	Df	*p*-value
0	Negative binomial, unadjusted for seasonality	373·0	ref		
1	Negative binomial, cosine function with 6-month period	379·2	2·32	2	0·31
2	Negative binomial, cosine function with 12-month period	381·2	0·26	2	0·88
3	Negative binomial, mixture of two cosine functions with 6-month and 12-month periods	385·9	4·02	4	0·40

- * All models adjusted for the doctors' and nurses' strike periods.
- † Likelihood ratio χ^2-test compared to Model 0.

Table A5.5.2: SMCH, Zimbabwe; Negative binomial model, unadjusted for seasonality (Model 0)

	Coef	SE	Exp	95% CI	*p*-value
Intercept	*-1·60*	*0·09*			
Post-COVID-19 period, yes	-0·33	0·17	0·72	0·52 – 1·00	0·05
Study time elapsed, weeks	0·00	0·00	1·00	1·00 – 1·01	0·24
Doctors' strike period, yes	0·19	0·10	1·21	0·99 – 1·48	0·07
Nurses' strike period, yes	0·59	0·16	1·81	1·31 – 2·49	< 0·001

Table A5.5.3: KCH, Malawi; Results of the models with and without adjustment for seasonality

Model		BIC	Deviance†	Df	*p*-value
0	Poisson, unadjusted for seasonality	343·1	ref		
1	Poisson, cosine function with 6-month period	349·7	1·86	2	0·39
2	Poisson, cosine function with 12-month period	349·7	1·90	2	0·39
3	Poisson, mixture of two cosine functions with 6-month and 12-month periods	355·4	4·69	4	0·32

- † χ^2-test compared to Model 0.

Table A5.5.4: KCH, Malawi; Poisson model, unadjusted for seasonality (Model 0)

	Coef	SE	Exp	95% CI	*p*-value
Intercept	*-1·56*	*0·09*			
Post-COVID-19 period, yes	0·27	0·15	1·31	0·97 – 1·76	0·08
Study time elapsed, weeks	-0·00	0·00	1·00	0·99 – 1·00	0·29

Chimhuya S, *et al. BMJ Open* 2022; 12:e048955. doi: 10.1136/bmjopen-2021-048955

APPENDIX 6: ADDITIONAL ANALYSES

Mode of delivery of admitted neonates

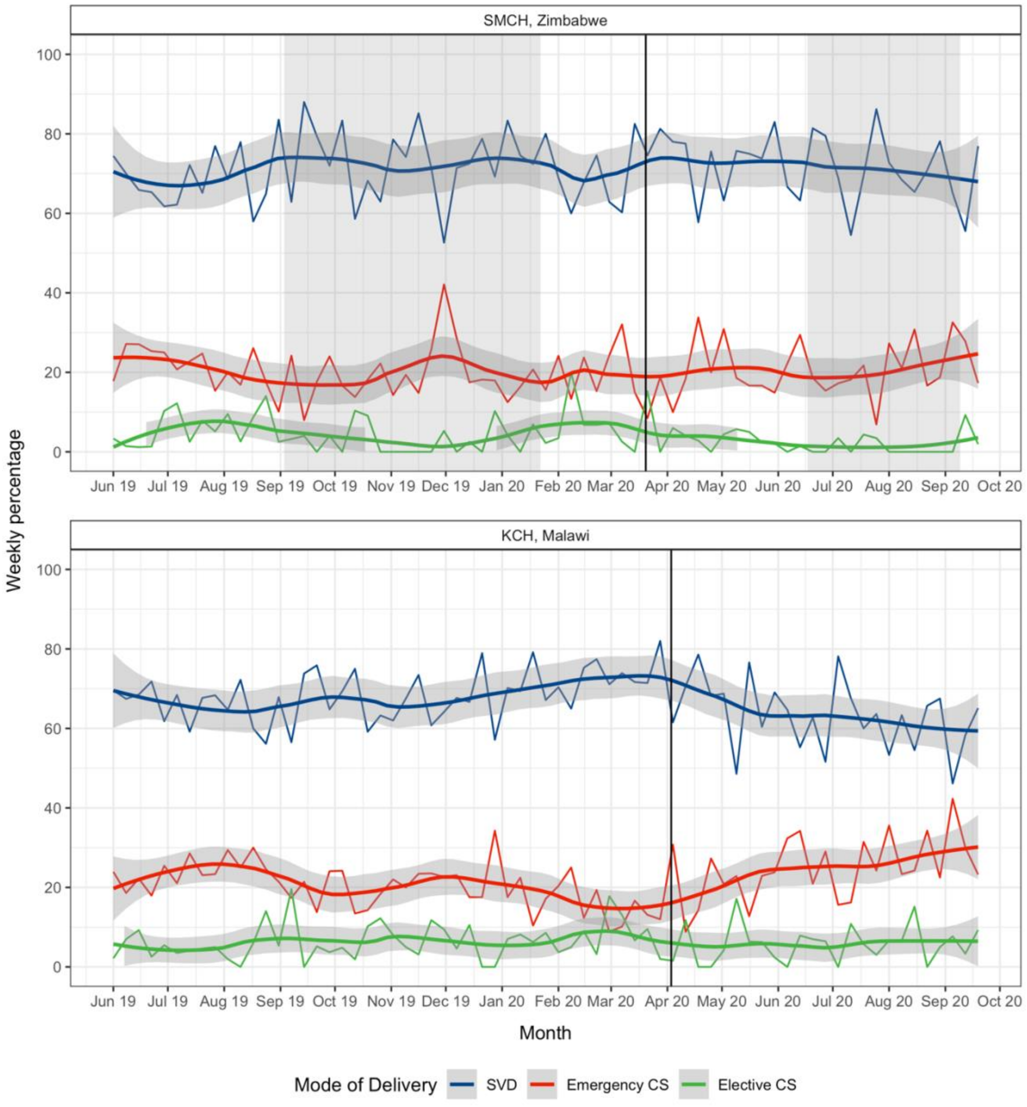

Figure A6.1.1: Trend in mode of delivery of admitted neonates per week

- Only SVD, emergency CS and elective CS displayed here to avoid overplotting.
- Smoothed line: local regression (LOESS) model; shaded region: 95% confidence interval.
- Solid vertical line: first confirmed case of COVID-19 in each country.
- Shaded periods on SMCH, Zimbabwe panel: industrial action by doctors (3 September 2019 to 22 January 2020) and nurses (17 July 2020 to 9 September 2020).
- Counts based on all admission forms completed, irrespective of match status.
- *SMCH: Sally Mugabe Central Hospital; KCH: Kamuzu Central Hospital; SVD: spontaneous vaginal delivery; CS: caesarean section*

Chimhuya S, *et al. BMJ Open* 2022; 12:e048955. doi: 10.1136/bmjopen-2021-048955

Reason for elective caesarean section

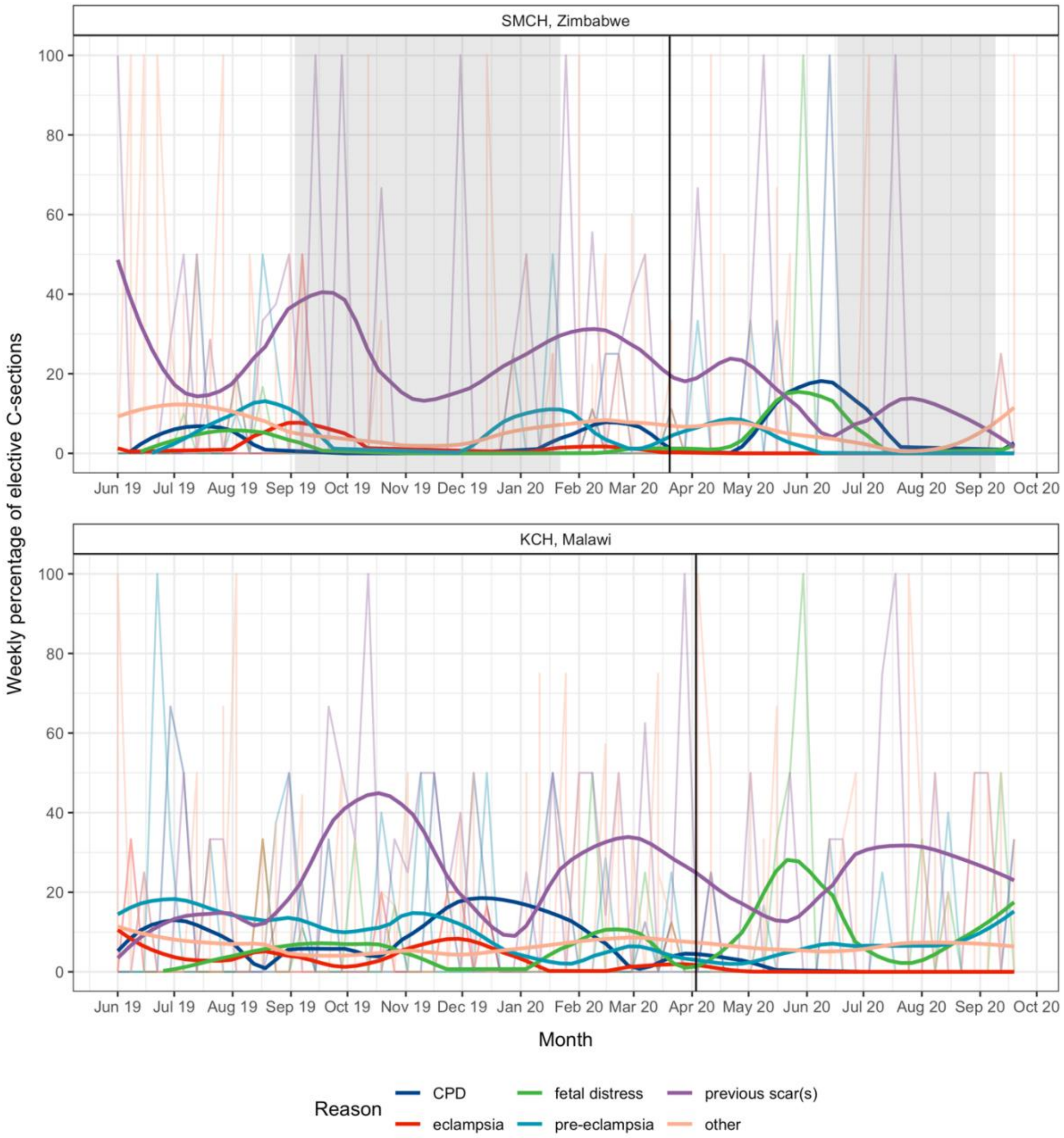

Figure A6.2.1: Trend in reason for elective caesarean section per week

- Smoothed line: local regression (LOESS) model; 95% confidence interval not presented to avoid overplotting.
- Solid vertical line: first confirmed case of COVID-19 in each country.
- Shaded periods on SMCH, Zimbabwe panel: industrial action by doctors (3 September 2019 to 22 January 2020) and nurses (17 July 2020 to 9 September 2020).
- Counts based on all admission forms completed, irrespective of match status.
- *SMCH: Sally Mugabe Central Hospital; KCH: Kamuzu Central Hospital; CPD: cephalopelvic disproportion*

Chimhuya S, *et al. BMJ Open* 2022; 12:e048955. doi: 10.1136/bmjopen-2021-048955

Reason for emergency caesarean section

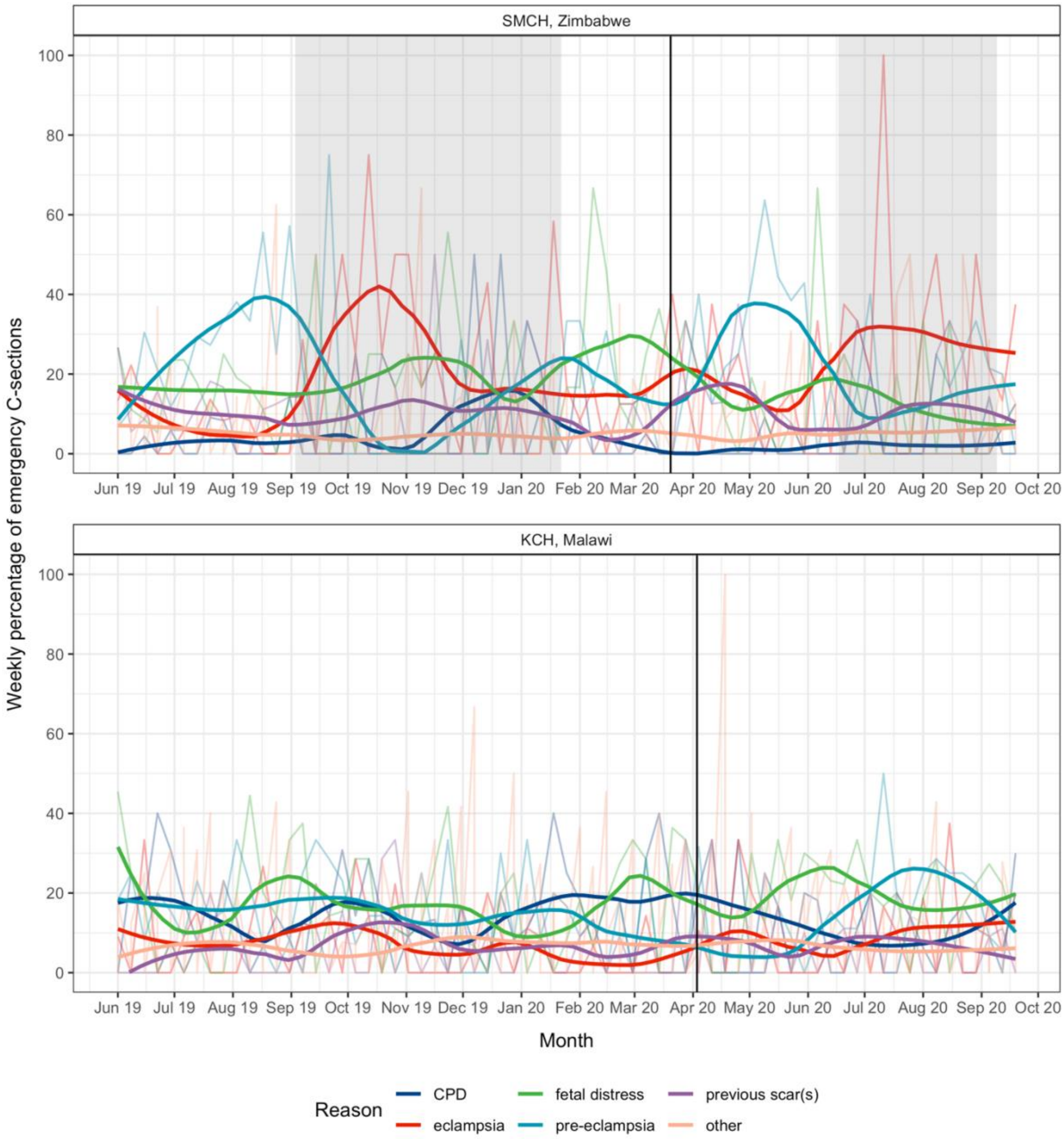

Figure A6.3.1: Trend in reason for emergency caesarean section per week

- Smoothed line: local regression (LOESS) model; 95% confidence interval not presented to avoid overplotting.
- Solid vertical line: first confirmed case of COVID-19 in each country.
- Shaded periods on SMCH, Zimbabwe panel: industrial action by doctors (3 September 2019 to 22 January 2020) and nurses (17 July 2020 to 9 September 2020).
- Counts based on all admission forms completed, irrespective of match status.
- *SMCH: Sally Mugabe Central Hospital; KCH: Kamuzu Central Hospital; CPD: cephalopelvic disproportion*

Chimhuya S, *et al. BMJ Open* 2022; 12:e048955. doi: 10.1136/bmjopen-2021-048955
