# Supplementary material for: Indirect impacts of the COVID-19 pandemic at two tertiary neonatal units in Zimbabwe and Malawi: an interrupted time series analysis
Source: BMJ Open. 2022 Jun 21;12(6):e048955. doi: 10.1136/bmjopen-2021-048955 (PMC9213761; doi:10.1136/bmjopen-2021-048955)
Supplement: Author's manuscript [file bmjopen-2021-048955.draft_revisions.pdf]

BMJ Open

BMJ Open is committed to open peer review. As part of this commitment we make the peer review history of every article we publish publicly available.

When an article is published we post the peer reviewers' comments and the authors' responses online. We also post the versions of the paper that were used during peer review. These are the versions that the peer review comments apply to.

The versions of the paper that follow are the versions that were submitted during the peer review process. They are not the versions of record or the final published versions. They should not be cited or distributed as the published version of this manuscript.

BMJ Open is an open access journal and the full, final, typeset and author-corrected version of record of the manuscript is available on our site with no access controls, subscription charges or pay-per-view fees (http://bmjopen.bmj.com).

If you have any questions on BMJ Open's open peer review process please email info.bmjopen@bmj.com

BMJ Open

Indirect impacts of the COVID-19 pandemic at two tertiary neonatal units in Zimbabwe and Malawi: an interrupted time series analysis

Journal:	*BMJ Open*
Manuscript ID	bmjopen-2021-048955
Article Type:	Original research
Date Submitted by the Author:	15-Jan-2021

Complete List of Authors:	Chimhuya, Simbarashe; University of Zimbabwe<br>Neal, Samuel; University College London, Institute of Child Health<br>Chimhini, Gwen; University of Zimbabwe<br>Cortina Borja, Mario; University College London, Institute of Child Health<br>Crehan, Caroline; University College London, Institute of Child Health<br>Nkhoma, Deliwe<br>Wilson, Emma; University College London, Institute of Child Health<br>Hull-Bailey, Tim; University College London, Institute of Child Health<br>Fitzgerald, Felicity<br>Heys, Michelle; University College London, Population<br>Gannon, Hannah; UCL<br>Chiyaka, Tarisai; Biomedical Research and Training Institute<br>Chiume, Msandeni; Kamuzu Central Hospital, Department of Pediatrics

Keywords:	COVID-19, PAEDIATRICS, Neonatal intensive & critical care < INTENSIVE & CRITICAL CARE, Quality in health care < HEALTH SERVICES ADMINISTRATION & MANAGEMENT, International health services < HEALTH SERVICES ADMINISTRATION & MANAGEMENT

Note: The following files were submitted by the author for peer review, but cannot be converted to PDF. You must view these files (e.g. movies) online.

figure2.eps
figure3.eps

SCHOLARONE™
Manuscripts

I, the Submitting Author has the right to grant and does grant on behalf of all authors of the Work (as defined in the below author licence), an exclusive licence and/or a non-exclusive licence for contributions from authors who are: i) UK Crown employees; ii) where BMJ has agreed a CC-BY licence shall apply, and/or iii) in accordance with the terms applicable for US Federal Government officers or employees acting as part of their official duties; on a worldwide, perpetual, irrevocable, royalty-free basis to BMJ Publishing Group Ltd ("BMJ") its licensees and where the relevant Journal is co-owned by BMJ to the co-owners of the Journal, to publish the Work in this journal and any other BMJ products and to exploit all rights, as set out in our licence.

The Submitting Author accepts and understands that any supply made under these terms is made by BMJ to the Submitting Author unless you are acting as an employee on behalf of your employer or a postgraduate student of an affiliated institution which is paying any applicable article publishing charge ("APC") for Open Access articles. Where the Submitting Author wishes to make the Work available on an Open Access basis (and intends to pay the relevant APC), the terms of reuse of such Open Access shall be governed by a Creative Commons licence – details of these licences and which Creative Commons licence will apply to this Work are set out in our licence referred to above.

Other than as permitted in any relevant BMJ Author's Self Archiving Policies, I confirm this Work has not been accepted for publication elsewhere, is not being considered for publication elsewhere and does not duplicate material already published. I confirm all authors consent to publication of this Work and authorise the granting of this licence.

For peer review only

Indirect impacts of the COVID-19 pandemic at two tertiary neonatal units in Zimbabwe and Malawi: an interrupted time series analysis

Simbarashe Chimhuya, MMED*[1], Samuel R. Neal, MRes*[2], Gwendoline Chimhini, MMED[1], Hannah Gannon, MBChB[2], Mario Cortina-Borja, PhD[2], Caroline Crehan, MSc[2], Deliwe Nkhoma, MSc[3], Tarisai Chiyaka, MSc[4], Emma Wilson, PhD[2], Tim Hull-Bailey, MPhil[2], Felicity Fitzgerald, PhD[5], Msandeni Chiume, MBBS ‡[6], and Michelle Heys, MD(Res)‡ † [2]

1. Child and Adolescent Health Unit, Faculty of Medicine and Health Sciences, University of Zimbabwe, Harare, Zimbabwe
2. Population, Policy and Practice Research and Teaching Department, UCL Great Ormond Street Institute of Child Health, University College London, UK
3. Parent and Child Health Initiative Trust, Lilongwe, Malawi
4. Biomedical Research and Training Institute, Harare, Zimbabwe
5. Infection, Immunity and Inflammation, UCL Great Ormond Street Institute of Child Health, University College London, UK
6. Department of Paediatrics, Kamuzu Central Hospital, Lilongwe, Malawi

*Contributed equally as first author
†Corresponding author
‡ Contributed equally as last author

Correspondence to:
Dr Michelle Heys
Population, Policy and Practice Department,
UCL Great Ormond Street Institute of Child Health,
30 Guilford Street,
London, WC1N 1EH
Email: m.heys@ucl.ac.uk
Telephone: +44 (0)20 7905 2212

Word count: 2917

Keywords: COVID-19, neonatal, NICU, low-resource settings, global health

Contributors' statement

Concept and study design by SC, SRN, GC, FF, MCB, CC, MC and MH with input from other authors. Data collected by HG, DN, TC, CC and THB. Analysis performed by SRN and MCB with contributions from FF, SC, EW & MH. Manuscript drafted by SC and SRN with input from GC, FF, MCB, MC & MH. All authors proof-read and approved final draft. Underlying data accessed and verified by SRN, MCB, HG, FF & MH.

For peer review only

ABSTRACT

Objectives: To examine indirect impacts of the COVID-19 pandemic on neonatal care in low-income and middle-income countries.

Design: Interrupted time series analysis.

Setting: Two tertiary neonatal units in Harare, Zimbabwe and Lilongwe, Malawi.

Participants: We included a total of 6,800 neonates who were admitted to either neonatal unit from 1 June 2019 to 25 September 2020 (Zimbabwe: 3,450; Malawi: 3,350). We applied no specific exclusion criteria.

Interventions: The first cases of COVID-19 in each country (Zimbabwe: 20 March 2020; Malawi: 3 April 2020).

Primary outcome measures: Changes in the number of admissions, gestational age and birth weight, source of admission referrals, prevalence of neonatal encephalopathy, and overall mortality before and after the first cases of COVID-19.

Results: Admission numbers in Zimbabwe did not initially change after the first case of COVID-19 but fell by 48% during a nurses' strike (relative risk (RR) 0·52, 95% CI 0·40-0·68, *p* < 0·001). In Malawi, admissions dropped by 42% soon after the first case of COVID-19 (RR 0·58, 95% CI 0·48-0·70, *p* < 0·001). In Malawi, gestational age and birth weight decreased slightly by around 1 week (beta -1·14, 95% CI -1·62-[-]0·65, *p* < 0·001) and 300 grams (beta -299·9, 95% CI -412·3-[-]187·5, *p* < 0·001), outside referrals dropped by 28% (RR 0·72, 95% CI 0·65-0·81, *p* < 0·001), and there was a slight weekly increase in mortality (RR 1·02 per week, 95% CI 1·00-1·04, *p* = 0·04). No changes in these outcomes were found in Zimbabwe and no changes in the prevalence of neonatal encephalopathy were found at either site (*p* > 0.05).

Conclusions: The indirect impacts of COVID-19 are context-specific. While our study provides vital evidence to inform health providers and policy makers, national data are required to ascertain the true impacts of the pandemic on newborn health.

Strengths and limitations of this study

- We address the need for increased research into the indirect impacts of the COVID-19 pandemic on neonatal care in low-income and middle-income countries.

For peer review only - http://bmjopen.bmj.com/site/about/guidelines.xhtml

- We collected data digitally and in real time using the NeoTree application, which enabled a large sample size of 6800 neonates with minimal missing data.
- It is possible that unobserved events occurred close to the first case of COVID-19 in either country, which could have influenced our results
- We only collected data on neonates admitted to the neonatal unit and did not capture stillbirths or neonatal deaths that occurred in the community.

For peer review only

For peer review only - http://bmjopen.bmj.com/site/about/guidelines.xhtml

LIST OF ABBREVIATIONS

app	application
CI	confidence interval
COVID-19	coronavirus disease 2019
KCH	Kamuzu Central Hospital
LMIC	low-income and middle-income country
NE	neonatal encephalopathy
NNU	neonatal unit
RR	Relative risk
SD	standard deviation
SMCH	Sally Mugabe Central Hospital

For peer review only

INTRODUCTION

The World Health Organization declared coronavirus disease (COVID-19) a Public Health Emergency of International Concern on 30 January 2020.[1] Confirmed cases have exceeded 80 million globally with nearly 2 million deaths.[2] Zimbabwe recorded its first case on 20 March and has reported >17000 cases with >400 deaths to date.[2] Malawi confirmed its first three cases on 3 April and has reported >7000 cases and ~200 deaths to date.[2]

Before the COVID-19 pandemic, considerable improvements were made in global child health: the global neonatal mortality rate fell from 31 to 18 deaths per 1,000 live births between 2000 and 2018.[3] Yet there were disparities in the rates of decline with the sub-Saharan Africa region facing highest neonatal mortality rates[3]. Now, there is a danger that health outcomes in low-income and middle-income countries (LMICs) will fall further behind high-income countries. While countries worldwide face challenges related to the COVID-19 pandemic, LMICs are particularly struggling with financial constraints, limited testing capacity, lack of personal protective equipment, and staff shortages.[4 5] As children are at low-risk of infection or severe disease from COVID-19,[6-10] any impacts on their health outcomes will likely be attributable to the indirect effects of the pandemic on health systems, as in previous disease outbreaks.[11 12] These include increased rates of parental unemployment, food and housing insecurity, and reduced access to routine care.[13 14]

The NeoTree application (app) is an Android tablet-based quality improvement platform that aims to reduce neonatal mortality in low-resource settings.[15] Developed in collaboration with local stakeholders, it is embedded in routine practice at two

For peer review only

neonatal units (NNUs) in Zimbabwe and Malawi, providing real-time clinical decision support, neonatal care education, and digital data capture.[16 17]

We aimed to examine trends in markers of neonatal care before and during the COVID-19 pandemic at Sally Mugabe Central Hospital (SMCH), Zimbabwe, and Kamuzu Central Hospital (KCH), Malawi. Specifically, we compared the:

1. number of admissions,
2. gestational age and birth weight of admitted neonates,
3. source of admission referrals,
4. prevalence of neonatal encephalopathy (NE), and
5. overall mortality rate

before and after the first reported cases of COVID-19.

For peer review only - http://bmjopen.bmj.com/site/about/guidelines.xhtml

METHODS

This study is reported in accordance with the Strengthening the Reporting of Observational Studies in Epidemiology (STROBE) statement (Appendix 1).

Setting

SMCH is a public referral hospital in Harare, Zimbabwe. It has the largest of three tertiary NNUs nationwide with 100 cots and predominantly doctor-led care. KCH, Lilongwe, is one of four regional referral hospitals in Malawi and the NNU has 75 cots. In contrast to SMCH, care in the NNU is mostly nurse-led. Both units accept local and national referrals for specialist surgical care.

Participants

All neonates admitted to each NNU over a 16-month period from 1 June 2019 to 25 September 2020 (69 complete weeks) were eligible for inclusion. We applied no specific exclusion criteria.

Data collection

Data were collected prospectively using the NeoTree app. Health workers complete a digital form when a neonate is admitted to the unit (admission form) and when they are discharged or die (outcome form). The app guides assessment of the neonate and collects data on patient demographics, examination findings, diagnoses, and interventions. Pseudonymised forms are uploaded monthly to University College London servers (Zimbabwe data) and Amazon Web Services (Malawi data). Admission and outcome forms are linked by a unique identifier generated by the app at admission.

For peer review only - http://bmjopen.bmj.com/site/about/guidelines.xhtml

Outcomes

We evaluated five outcomes:

1. Number of admissions: determined from the admission date of each completed admission form.
2. Gestational age at birth (weeks) and birth weight (grams): as entered into the admission form from obstetric records.
3. Source of admission: defined as 'within' (labour ward, postnatal ward, antenatal ward, obstetric theatre, or fee-paying ward [KCH only]) or 'outside' (referral from another health facility or postnatal self-referral from home).
4. Diagnosis of NE: defined as "hypoxic ischaemic encephalopathy" or "birth asphyxia" recorded as a diagnosis, cause of death or contributory cause of death on the outcome form.
5. Mortality: defined as an outcome of "neonatal death" on the outcome form. All other neonates, including those discharged, transferred to another facility or who left on parental request, were considered alive.

Ethical approval

Research ethics approval was granted by the UCL Research Ethics Committee (17123/001) and ethics committees in Malawi (P.01/20/2909) and Zimbabwe (MRCZ/A/2570) (Appendix 2). The need to obtain informed consent was waived as we collected only pseudonymised data routinely documented for clinical care.

For peer review only

Statistical analysis

Analyses were performed in R version 3.6.3,[18] running on RStudio version 1.2.5033.[19] First, admission forms were matched with their corresponding outcome form based on the unique identifier generated at admission. Lack of completed outcome forms (SMCH: *n*=316[9.1%]; KCH: *n*=243[7.2%]) or errors in entry of the unique identifier at discharge (SMCH: *n*=318[9.2%]; KCH: *n*=182[5.4%]) meant we were unable to match some admission forms with outcome forms (SMCH: *n*=634[18.3%]; KCH: *n*=425[12.6%]). For outcomes 1-3, we based analyses on data from all admission forms, regardless of match status. For outcomes 4 and 5, we based analyses on matched records only. Matched records implying a negative admission duration (i.e. outcome date prior to admission date) were excluded (SMCH: *n*=58[2%]; KCH: *n*=25[1%]). See Appendix 3 for a flow diagram of record inclusion. Missing data were excluded using pairwise deletion for each analysis as frequencies of missing values were minimal (Appendix 4).

This study used an interrupted time series design with weekly data windows. We considered the first confirmed case of COVID-19 in each country as the intervention (Zimbabwe: 20 March 2020; Malawi: 3 April 2020).[2] For all outcomes, we hypothesised a level change impact model without a lag (for a description of these models, see Bernal et al.[20]). Gestational age and birth weight were modelled with linear regression. All other outcomes were modelled using quasi-Poisson regression to account for overdispersion,[21] with the logarithm of the number of admissions in each weekly window included as an offset. All SMCH models were adjusted for a period of doctors' strikes from 3 September 2019 to 22 January 2020.[22] KCH models were unadjusted. Additional models were constructed to explore the effects of a nurses' strike in

For peer review only - http://bmjopen.bmj.com/site/about/guidelines.xhtml

Zimbabwe (17 June to 9 September 2020)[23] and alternative impact models. Nested models were compared with the *F*-test. See Appendix 5 for model details.

Patient and Public Involvement

Although patients and the public were not directly involved in this study, within the broader NeoTree co-development project we are carrying out a series of workshops and focus group discussions with healthcare workers and parents of admitted babies to ensure local ownership and relevance of this digital quality involvement tool aimed at improving healthcare outcomes for vulnerable neonates.

Role of the funding source

The funders had no role in study design, data collection, data analysis, data interpretation, or preparation of this manuscript.

For peer review only

RESULTS

Outcome 1: Admissions to the neonatal unit

We included 3,450 neonates at SMCH and 3,350 neonates at KCH. Figure 1 shows the seven-day moving average of admissions to the NNU.

At SMCH, the mean (SD) number of weekly admissions was 54·6 (23·5) before the first case of COVID-19 (pre-COVID-19) and 42·8 (19·9) afterwards (post-COVID-19). The level change regression model, adjusted for the doctors' strike, showed no evidence of a change in admissions after the first case of COVID-19 (relative risk [RR] 0·83; 95% confidence interval [CI] 0·60-1·14; p = 0·25) but the scatterplot indicated this model fit the data poorly (model 1, Figure 2A). An alternative model, additionally adjusted for the nurses' strike, again showed no change in the overall post-COVID-19 period (RR 0·90; 95%CI 0·69-1·17; p = 0·43) (model 2, Figure 2B). However, this model suggested that admissions fell by 48% during the nurses' strike period (RR 0·52, 95%CI 0·40-0·68, p < 0·001) and fit the data better (F[1, 64] = 24·66, p < 0·001).

At KCH, the mean (SD) number of weekly admissions was 54·5 (10·8) in the pre-COVID-19 period and 38·0 (10·9) in the post-COVID-19 period. The level change model suggested a 42% reduction in admissions after the first case of COVID-19 (RR 0·58; 95%CI 0·48-0·70; p < 0·001) (Figure 2C).

For peer review only - http://bmjopen.bmj.com/site/about/guidelines.xhtml

Outcome 2: Gestational age and birth weight

At SMCH, the mean (SD) gestational age at birth was 36·1 (4·4) weeks in the pre-COVID-19 period and 36·0 (4·2) weeks in the post-COVID-19 period. The mean (SD) birth weight was 2500 (908) grams in the pre-COVID-19 period and 2487 (896) grams in the post-COVID-19 period. Regression analysis indicated no change in gestational age at birth nor birth weight after the first case of COVID-19 (gestational age: beta 0·04; 95%CI -0·53-0·61; *p* = 0·89, birth weight: beta -7·2; 95%CI -127·1-112·6; *p* = 0·91) (Supplementary Figure 1A, Supplementary Figure 1C,). Adjusting for the nurses' strike did not improve model fit (data not shown).

At KCH, the mean (SD) gestational age was 35·0 (3·9) weeks in the pre-COVID-19 period and 34·8 (3·9) weeks in the post-COVID-19 period. The mean (SD) birth weight was 2402 (883) grams in the pre-COVID-19 period and 2299 (870) grams in the post-COVID-19 period. Gestational age decreased by one week in the post-COVID-19 period (beta -1·14; 95%CI -1·62-[-]0·65; *p* < 0·001) (Supplementary Figure 1B) and birth weight decreased by 300 grams (beta -299·9; 95%CI -412·3-[-]187·5; *p* < 0·001) (Supplementary Figure 1D).

Outcome 3: Source of admission referral

At SMCH, the mean (SD) percentage of outside referrals to the NNU was 39(11)% in the pre-COVID-19 period and 35(9)% in the post-COVID-19 period. The regression model showed no evidence of a change in the percentage of outside referrals after the first case of COVID-19 (RR 0·98; 95%CI 0·79-1·23; *p* = 0·88) (Figure 3A). Adjusting for the nurses' strike did not improve model fit (data not shown).

For peer review only - http://bmjopen.bmj.com/site/about/guidelines.xhtml

At KCH, the mean (SD) percentage of outside referrals was 61(8)% in the pre-COVID-19 period and 51(10)% in the post-COVID-19 period. Regression analysis suggested a 28% relative reduction in outside referrals after the first case of COVID-19 (RR 0·72; 95%CI 0·65-0·81; p < 0·001) (Figure 3B).

Outcome 4: Prevalence of neonatal encephalopathy

At SMCH, the mean (SD) percentage of admitted neonates diagnosed with NE was 16(6)% in the pre-COVID-19 period and 21(12)% in the post-COVID-19 period suggesting a possible increase. Regression analysis showed no statistically significant change in the percentage of neonates diagnosed with NE post-COVID-19 (RR 1·08; 95%CI 0·76-1·55; p = 0·67) (Supplementary Figure 2A). Adjusting for the nurses' strike did not improve model fit (data not shown).

At KCH, the mean (SD) percentage of admitted neonates diagnosed with NE was 15(6)% in the pre-COVID-19 period and 13(5)% in the post-COVID-19 period. The regression model suggested a possible increase in diagnoses of NE after the first case of COVID-19, but this was not statistically significant (RR 1·30; 95%CI 0·95-1·80; p = 0·11) (Supplementary Figure 2B).

Outcome 5: Overall mortality

For SMCH, the mean (SD) percentage of deaths per week of admission was 25(10)% in the pre-COVID-19 period and 26(16)% in the post-COVID-19 period. The level change regression model, adjusted for the doctors' strike, showed no evidence of a

For peer review only - http://bmjopen.bmj.com/site/about/guidelines.xhtml

change in mortality after the first case of COVID-19 (RR 0·80; 95%CI 0·56-1·15; *p* = 0·23) but the scatterplot indicated this model fit the data poorly (model 1, Figure 4A). An alternative model, additionally adjusted for the nurses' strike, again showed no change in overall mortality (RR 0·72; 95%CI 0·51-1·03; *p* = 0·07) but fit the data better (F[1, 64] = 11·61, *p* = 0·001) (model 2, Figure 4B).

For KCH, the mean (SD) percentage of deaths per week of admission was 19(6)% in the pre-COVID-19 period and 23(9)% in the post-COVID-19 period. The level change regression model suggested a possible increase in mortality after the first case of COVID-19, but this was not statistically significant (RR 1·31; 95%CI 0·98-1·73; *p* = 0·07) (Figure 4C). However, fitting a slope change impact model suggested a small relative increase in mortality by 2% per week in the post-COVID-19 period (RR 1·02 per week; 95%CI 1·00-1·04, *p* = 0·04) (Figure 4D).

DISCUSSION

Summary

We performed an interrupted time series analysis to examine changes in neonatal care provision at two tertiary NNUs in Zimbabwe and Malawi after the first cases of COVID-19. We found that admissions at SMCH did not change significantly after the first case of COVID-19 when considering this period as a whole, but there was a considerable decrease (~50%) in the number admissions in June to August 2020, coinciding with a nurses' strike. We did not find significant changes in gestational age or birth weight, source of admission referrals, prevalence of NE or mortality at SMCH. Conversely, we found several changes in markers of neonatal care at KCH after the first case of COVID-19 in Malawi. The number of admissions fell by 42% and we noted

For peer review only - http://bmjopen.bmj.com/site/about/guidelines.xhtml

a decrease in the gestational age and birth weight of admitted neonates (by ~1 week and ~300 grams, respectively), a 28% relative decrease in outside referrals, and a small but statistically significant weekly increase in mortality by 2% after the first case of COVID-19. Although this study is descriptive, we can speculate about explanations for our results based on existing literature and discussions with local health workers.

Interpretation

The number of admissions at SMCH fell by around 50% between June to August 2020, but we noted no change outside this strike period, suggesting some resilience to the impact of the pandemic. However, nurses went on strike over pay and availability of personal protective equipment,[23] so the strike is itself an indirect consequence of COVID-19. A similar reduction in admissions was seen at KCH, but, unlike at SMCH, this 42% decrease was noted within a week of the first case of COVID-19. In Figure 5, we propose several interlinked factors that might explain reduced admissions to the NNU. Several of these factors, such as fear of using health services, disrupted transport networks and staff shortages have been directly reported by local sources in low-resource settings and were highlighted in a recent report by Graham et al.[24]

We found a slight decrease in gestational age and birth weight of neonates at KCH, but not SMCH. Studies have reported increased rates of preterm birth in pregnant women with COVID-19 compared to those without the disease, mostly from medically-induced preterm birth; although none of these studies were conducted in LMICs.[25] Preliminary analysis suggests rates of emergency caesarean section increased at SMCH and KCH, with a more marked increase at KCH (Appendix 6). This is one potential explanation for our findings. However, we noted that the number of outside

For peer review only - http://bmjopen.bmj.com/site/about/guidelines.xhtml

referrals decreased by 28% at KCH, and neonates referred from outside KCH are more likely to be from lower-risk pregnancies that delivered in a health centre with higher gestational ages and birth weights. Further analysis should stratify by source of admission referral to clarify this finding, but is supported by the fact that referrals were rigorously triaged by the on-call paediatrician during the pandemic, and that referrals from some areas were diverted away from KCH.

We hypothesised that rates of NE would increase during the pandemic. NE is the clinical manifestation of disordered brain function and can have multiple aetiologies.[26] The term 'hypoxic-ischaemic encephalopathy' is reserved for cases where there is evidence of intrapartum asphyxia.[26] In LMICs, obstructed labour is a major cause of maternal mortality and can lead to intrapartum asphyxia with subsequent neonatal morbidity and mortality, including NE.[27] Therefore, the prevalence of NE might be expected to increase as a marker of delayed presentation to a health facility. It is reassuring that we did not find increased rates of NE at SMCH or KCH. However, these findings should be interpreted cautiously as some neonates with NE may not have presented to a health facility at all.

Finally, we observed a slight increase in overall mortality at KCH (a relative increase of 2% per week after the first case of COVID-19), although not at SMCH. In KCH, the increase in mortality may be due to decreased gestational age and birthweight, but also due to a reduced rota of nursing staff implemented to protect healthcare workers. In fact, there was a suggestion that mortality decreased after the first case of COVID-19 in Zimbabwe, but this was not statistically significant. The reasons for this are unclear but could include factors such as increased stillbirth rates or improved care for

For peer review only

the smaller number of neonates on the NNU. More complete analysis of facility-based and community-based neonatal mortality is greatly needed.

Limitations and future work

Some limitations should be noted. A limitation intrinsic to interrupted time series analysis is the possibility that another event occurred close to the first case of COVID-19 in either country causing spurious observations. Another potential threat to validity is changing data collection practices. For example, overstretched clinicians might not input data into the NeoTree app for all admitted neonates. However, this is unlikely as the NeoTree app is embedded into routine practice at SMCH and KCH and discussions with local collaborators suggest use of the app has continued without issue.

The NeoTree app only collects data on neonates admitted to the NNU. Therefore, our analysis does not capture stillbirths or neonatal deaths that occur in the community. It is troubling to see a dramatic fall in admissions in both sites, raising the possibility that many unwell neonates did not attend a health facility and died at home. A recent study found that facility births decreased by over 50% during the lockdown in Nepal, and facility stillbirth and neonatal mortality rates increased significantly.[28] The NeoTree research team is currently collecting data on stillbirths at SMCH and KCH, but these data will still only represent stillbirths that occurred in a health facility. Given the COVID-19 pandemic is not over, it will be important to repeat our analysis over the coming months to further examine longer-term trends in neonatal care provision.

For peer review only - http://bmjopen.bmj.com/site/about/guidelines.xhtml

Conclusion

The indirect impacts of COVID-19 are context-specific, with more significant and evident effects on neonatal care provision seen at KCH (Malawi) than SMCH (Zimbabwe). While this study provides vital evidence to inform health providers and policy makers, national data are required to ascertain the true impacts of the pandemic on newborn health.

Figure Legends

Figure 1: Trend in daily admissions to the neonatal unit

- The seven-day moving average of daily admission numbers has been plotted.
- Smoothed line: local regression (LOESS) model fitted on the seven-day moving average of daily admission numbers; shaded region: 95% confidence interval.
- Solid vertical line: first confirmed case of COVID-19 in each country.
- Period between dashed vertical lines: industrial action by doctors in Zimbabwe.
- Counts based on all admission forms completed, irrespective of match status.
- *SMCH: Sally Mugabe Central Hospital; KCH: Kamuzu Central Hospital*

Figure 2: Interrupted time series for weekly admissions to the neonatal unit

- White background: pre-COVID-19 period; grey background: post-COVID-19 period.
- Solid line: predicted trend from regression model; dashed line: counterfactual scenario.
- SMCH model 1 (panel A) adjusted for doctors' strike period; SMCH model 2 (panel B) additionally adjusted for nurses' strike period; KCH model (panel C) unadjusted.
- Counts based on all admission forms completed, irrespective of match status.
- *SMCH: Sally Mugabe Central Hospital; KCH: Kamuzu Central Hospital*

Figure 3: Interrupted time series for outside referrals to the neonatal unit

- White background: pre-COVID-19 period; grey background: post-COVID-19 period.
- Solid line: predicted trend from regression model; dashed line: counterfactual scenario.
- SMCH model (panel A) adjusted for doctors' strike period, KCH model (panel B) unadjusted.
- Data from all admission forms completed, irrespective of match status.
- *SMCH: Sally Mugabe Central Hospital; KCH: Kamuzu Central Hospital*

For peer review only - http://bmjopen.bmj.com/site/about/guidelines.xhtml

Figure 4: Interrupted time series for overall mortality

- White background: pre-COVID-19 period; grey background: post-COVID-19 period.
- Solid line: predicted trend from regression model; dashed line: counterfactual scenario.
- SMCH model 1 (panel A) adjusted for doctors' strike period; SMCH model 2 (panel B) additionally adjusted for nurses' strike period; KCH model 1 (panel C) unadjusted level change model; KCH model 2 (panel D) unadjusted slope change model.
- Data from matched admission and outcome forms only.
- *SMCH: Sally Mugabe Central Hospital; KCH: Kamuzu Central Hospital*

Figure 5: Possible factors influencing the decrease in admissions to the neonatal unit

- Delays (red boxes) derived from the "Three Delays" model of pregnancy-related mortality.[29]
- *COVID-19: coronavirus disease 2019; PPE: personal protective equipment*

For peer review only

REFERENCES

1. World Health Organization. Statement on the second meeting of the International Health Regulations (2005) Emergency Committee regarding the outbreak of novel coronavirus (2019-nCoV) 2020 [cited 2020 04 Sept]. Available from: https://bit.ly/3jihlAU accessed 04 Sept 2020.

2. World Health Organization. WHO Coronavirus Disease (COVID-19) Dashboard 2020 [updated 2020 21 Oct; cited 2020 21 Oct]. Available from: https://covid19.who.int accessed 21 Oct 2020.

3. United Nations Inter-agency Group for Child Mortality Estimation. Levels & Trends in Child Mortality: Report 2019. New York: United Nations Children's Fund, 2019.

4. Makoni M. COVID-19 worsens Zimbabwe's health crisis. *Lancet* 2020;396(10249):457. doi: 10.1016/S0140-6736(20)31751-7

5. Truscott R. Covid-19: Health worker strikes, limited testing, and clinic closures hamper Zimbabwe's response. *BMJ* 2020;370:m3267. doi: 10.1136/bmj.m3267

6. Castagnoli R, Votto M, Licari A, et al. Severe Acute Respiratory Syndrome Coronavirus 2 (SARS-CoV-2) Infection in Children and Adolescents: A Systematic Review. *JAMA Pediatr* 2020 doi: 10.1001/jamapediatrics.2020.1467 [published Online First: 2020/04/23]

7. Götzinger F, Santiago-García B, Noguera-Julián A, et al. COVID-19 in children and adolescents in Europe: a multinational, multicentre cohort study. *Lancet Child Adolesc Health* 2020;4(9):653-61. doi: 10.1016/S2352-4642(20)30177-2

8. Ludvigsson JF. Systematic review of COVID-19 in children shows milder cases and a better prognosis than adults. *Acta Paediatr* 2020;109(6):1088-95. doi: 10.1111/apa.15270

9. Mantovani A, Rinaldi E, Zusi C, et al. Coronavirus disease 2019 (COVID-19) in children and/or adolescents: a meta-analysis. *Pediatr Res* 2020 doi: 10.1038/s41390-020-1015-2

10. Viner RM, Mytton OT, Bonell C, et al. Susceptibility to and transmission of COVID-19 amongst children and adolescents compared with adults: a systematic review and meta-analysis. *medRxiv* 2020:2020.05.20.20108126. doi: 10.1101/2020.05.20.20108126

11. Sochas L, Channon AA, Nam S. Counting indirect crisis-related deaths in the context of a low-resilience health system: the case of maternal and neonatal health during the Ebola epidemic in Sierra Leone. *Health Policy Plan* 2017;32(suppl_3):iii32-iii39. doi: 10.1093/heapol/czx108

12. Yerger P, Jalloh M, Coltart CEM, et al. Barriers to maternal health services during the Ebola outbreak in three West African countries: a literature review. *BMJ Glob Health* 2020;5(9) doi: 10.1136/bmjgh-2020-002974 [published Online First: 2020/09/09]

For peer review only

13. Ahmed S, Mvalo T, Akech S, et al. Protecting children in low-income and middle-income countries from COVID-19. *BMJ Glob Health* 2020;5(5) doi: 10.1136/bmjgh-2020-002844 [published Online First: 2020/05/29]

14. Roberton T, Carter ED, Chou VB, et al. Early estimates of the indirect effects of the COVID-19 pandemic on maternal and child mortality in low-income and middle-income countries: a modelling study. *Lancet Glob Health* 2020;8(7):e901-e08. doi: 10.1016/s2214-109x(20)30229-1 [published Online First: 2020/05/15]

15. NeoTree. NeoTree [GitHub repository]. 2020 [cited 2020 03 Oct]. Available from: https://github.com/neotree/neotree accessed 03 Oct 2020.

16. Crehan C, Kesler E, Nambiar B, et al. The NeoTree application: developing an integrated mHealth solution to improve quality of newborn care and survival in a district hospital in Malawi. *BMJ Glob Health* 2019;4(1):e000860. doi: 10.1136/bmjgh-2018-000860 [published Online First: 2019/02/05]

17. Gannon H, Chimhuya S, Chimhini G, et al. An electronic application to improve management of infections in low-income neonatal units: pilot implementation of the NeoTree Beta App in a public sector hospital in Zimbabwe. *medRxiv* 2020:2020.09.25.20201467. doi: 10.1101/2020.09.25.20201467

18. R: A language and environment for statistical computing [program]. 3.6.3 version. Vienna, Austria: R Foundation for Statistical Computing, 2020.

19. RStudio: Integrated Development Environment for R [program]. 1.2.5033 version. Boston, MA: RStudio, Inc., 2019.

20. Bernal JL, Cummins S, Gasparrini A. Interrupted time series regression for the evaluation of public health interventions: a tutorial. *Int J Epidemiol* 2017;46(1):348-55. doi: 10.1093/ije/dyw098

21. Wedderburn RWM. Quasi-likelihood functions, generalized linear models, and the Gauss—Newton method. *Biometrika* 1974;61(3):439-47. doi: 10.1093/biomet/61.3.439

22. BBC News. Zimbabwe doctors end strike after billionaire's offer 2020 [cited 2020 04 Aug]. Available from: https://www.bbc.co.uk/news/world-africa-51205619 accessed 04 Aug 2020.

23. Reuters. Zimbabwe nurses end three-month strike over pay 2020 [cited 2020 20 Oct]. Available from: https://www.reuters.com/article/us-health-corononavirus-zimbabwe-strike-idUSKBN26011R accessed 20 Oct 2020.

24. Graham WJ, Afolabi B, Benova L, et al. Protecting hard-won gains for mothers and newborns in low-income and middle-income countries in the face of COVID-19: call for a service safety net. *BMJ Glob Health* 2020;5(6) doi: 10.1136/bmjgh-2020-002754 [published Online First: 2020/06/06]

25. Khalil A, Kalafat E, Benlioglu C, et al. SARS-CoV-2 infection in pregnancy: A systematic review and meta-analysis of clinical features and pregnancy outcomes. *EClinicalMedicine* 2020;25 doi: 10.1016/j.eclinm.2020.100446

For peer review only - http://bmjopen.bmj.com/site/about/guidelines.xhtml

26. Robertson NJ, Groenendaal F. Hypoxic-ischaemic brain injury. In: Rennie JM, ed. Rennie and Roberton's Textbook of Neonatology. 5th ed. London: Churchill Livingstone 2012:1114-55.

27. Say L, Chou D, Gemmill A, et al. Global causes of maternal death: a WHO systematic analysis. *Lancet Glob Health* 2014;2(6):e323-e33. doi: 10.1016/S2214-109X(14)70227-X

28. Kc A, Gurung R, Kinney MV, et al. Effect of the COVID-19 pandemic response on intrapartum care, stillbirth, and neonatal mortality outcomes in Nepal: a prospective observational study. *Lancet Glob Health* 2020;8(10):e1273-e81. doi: 10.1016/S2214-109X(20)30345-4

29. Thaddeus S, Maine D. Too far to walk: maternal mortality in context. *Soc Sci Med* 1994;38(8):1091-110. doi: 10.1016/0277-9536(94)90226-7 [published Online First: 1994/04/01]

For peer review only

Declaration of interests

The authors have no conflicts of interest to declare.

Acknowledgements

We are very grateful to the families at SMCH and KCH, and the staff members at both hospitals for their enthusiasm and commitment to the NeoTree project, without which this work would not be possible.

Funders

We would like to thank the funders of this study. Mr S. R. Neal was awarded the International Child Health Group David Morley Elective Bursary for this elective project. Funders of the wider NeoTree project, past and present, include the Wellcome Trust Digital Innovation Award (215742/Z/19/Z: PI: Heys), RCPCH, Naughton-Cliffe Mathews, UCL Grand Challenges and Global Engagement Fund, and the Healthcare Infection Society (SRG 201802004). Dr F. Fitzgerald is supported by the Academy of Medical Sciences and the funders of the Starter Grants for Clinical Lecturers scheme. This study and Drs M. Heys and F. Fitzgerald are further supported by the National Institute for Health Research Great Ormond Street Hospital Biomedical Research Centre. The funders had no role in study design, data collection and analysis, or preparation of this report.

Data sharing statement

Data collected for the study cannot yet be made publicly available yet because primary analysis for the pilot implementation evaluation of the NeoTree, as well as secondary analysis are ongoing. A goal of our pilot implementation is the establishment of an open-source anonymised research database of data collected using the NeoTree in order to maximise the reach and utility for researchers aiming to improve outcomes for neonates in low income settings. This database is under development and subject to negotiation with relevant Ministries of Health.

For peer review only

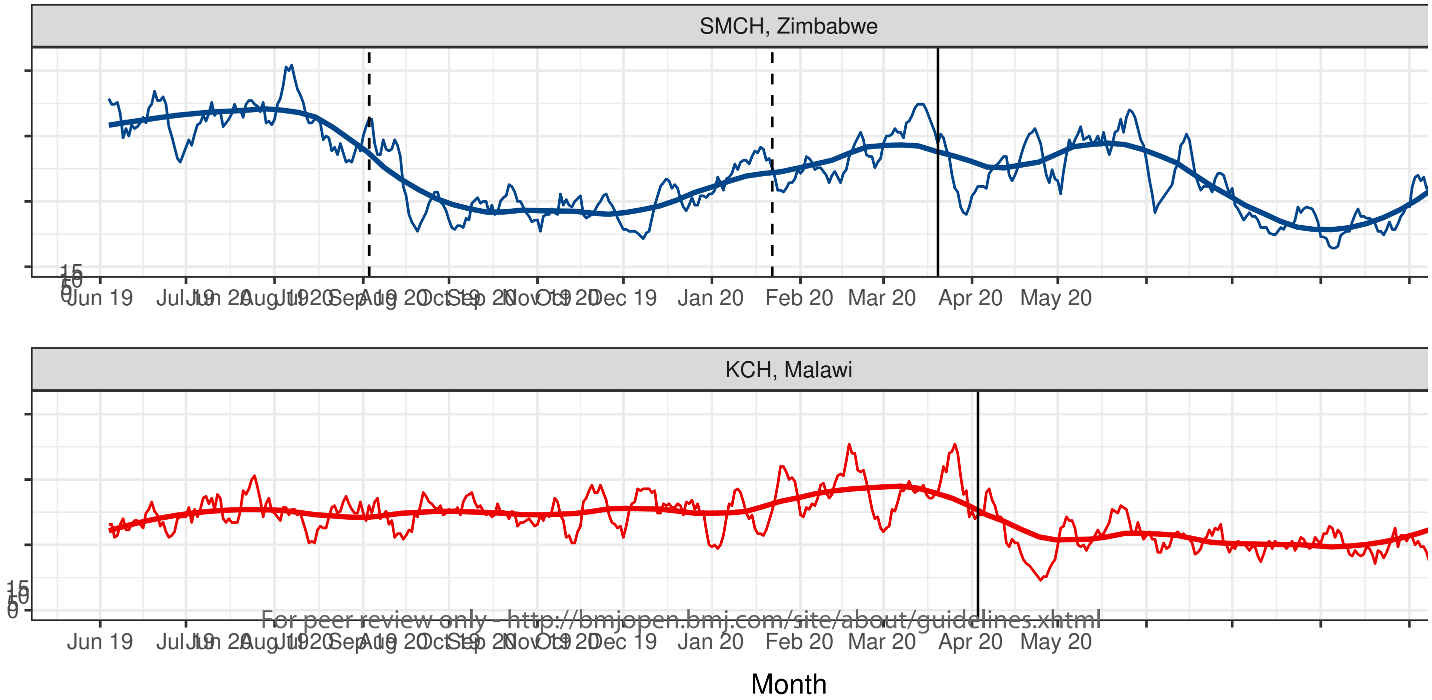

For peer review only - http://bmjopen.bmj.com/site/about/guidelines.xhtml

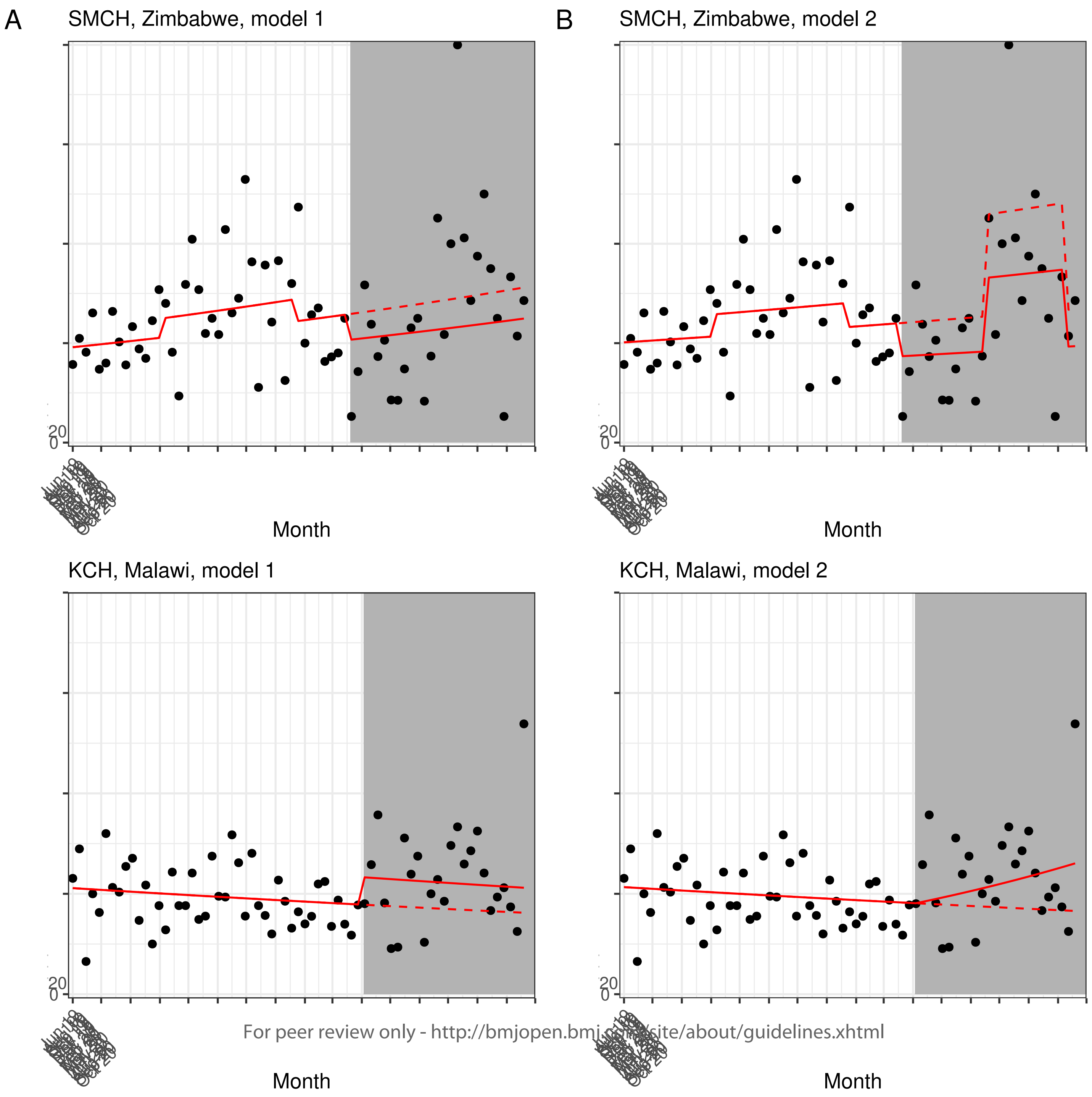
A
SMCH, Zimbabwe, model 1
B
SMCH, Zimbabwe, model 2
KCH, Malawi, model 1
KCH, Malawi, model 2
Month
Month
Month
Month

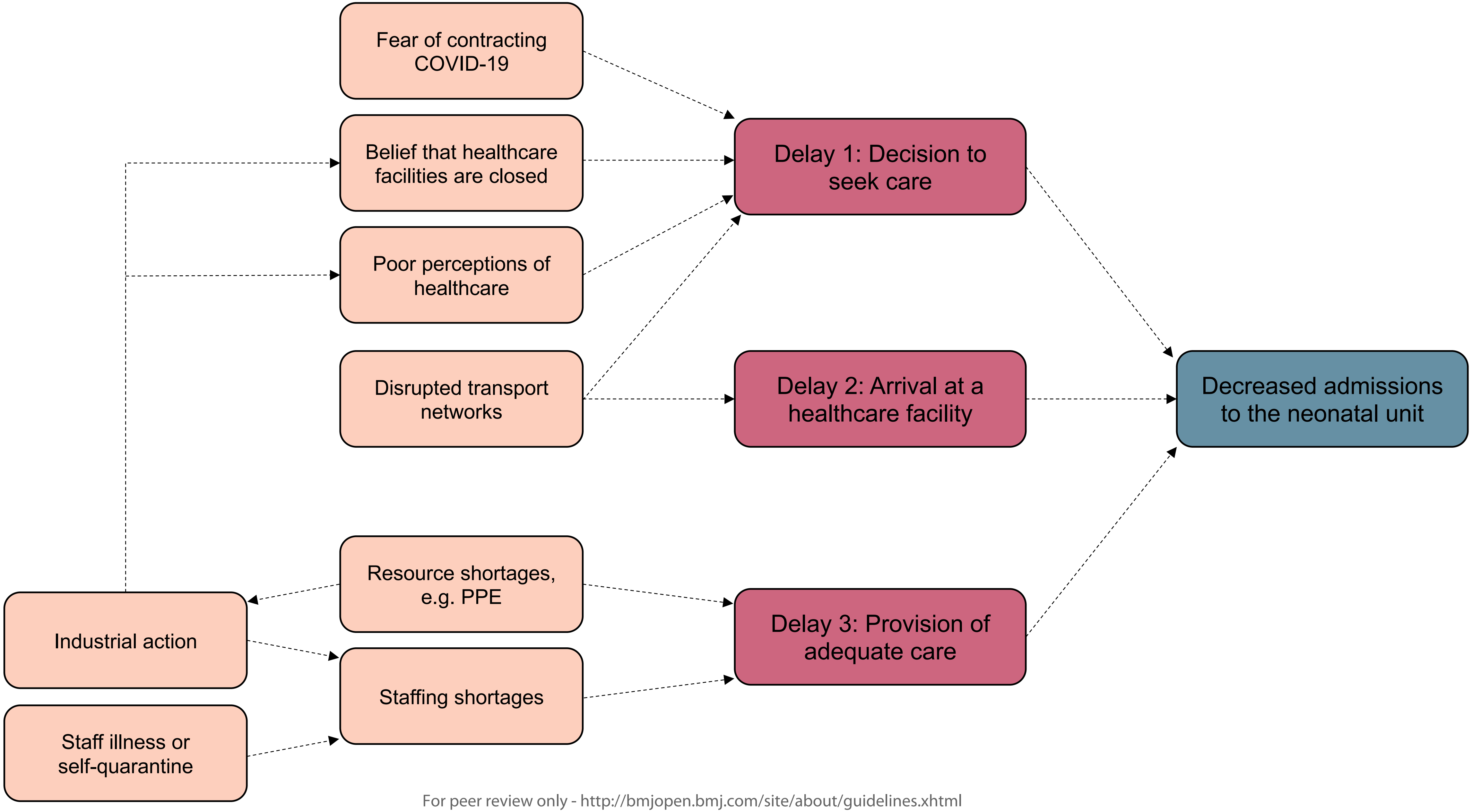
Fear of contracting COVID-19
Belief that healthcare facilities are closed
Poor perceptions of healthcare
Disrupted transport networks
Delay 1: Decision to seek care
Delay 2: Arrival at a healthcare facility
Decreased admissions to the neonatal unit
Resource shortages, e.g. PPE
Industrial action
Staffing shortages
Delay 3: Provision of adequate care
Staff illness or self-quarantine

SUPPLEMENTARY FIGURES

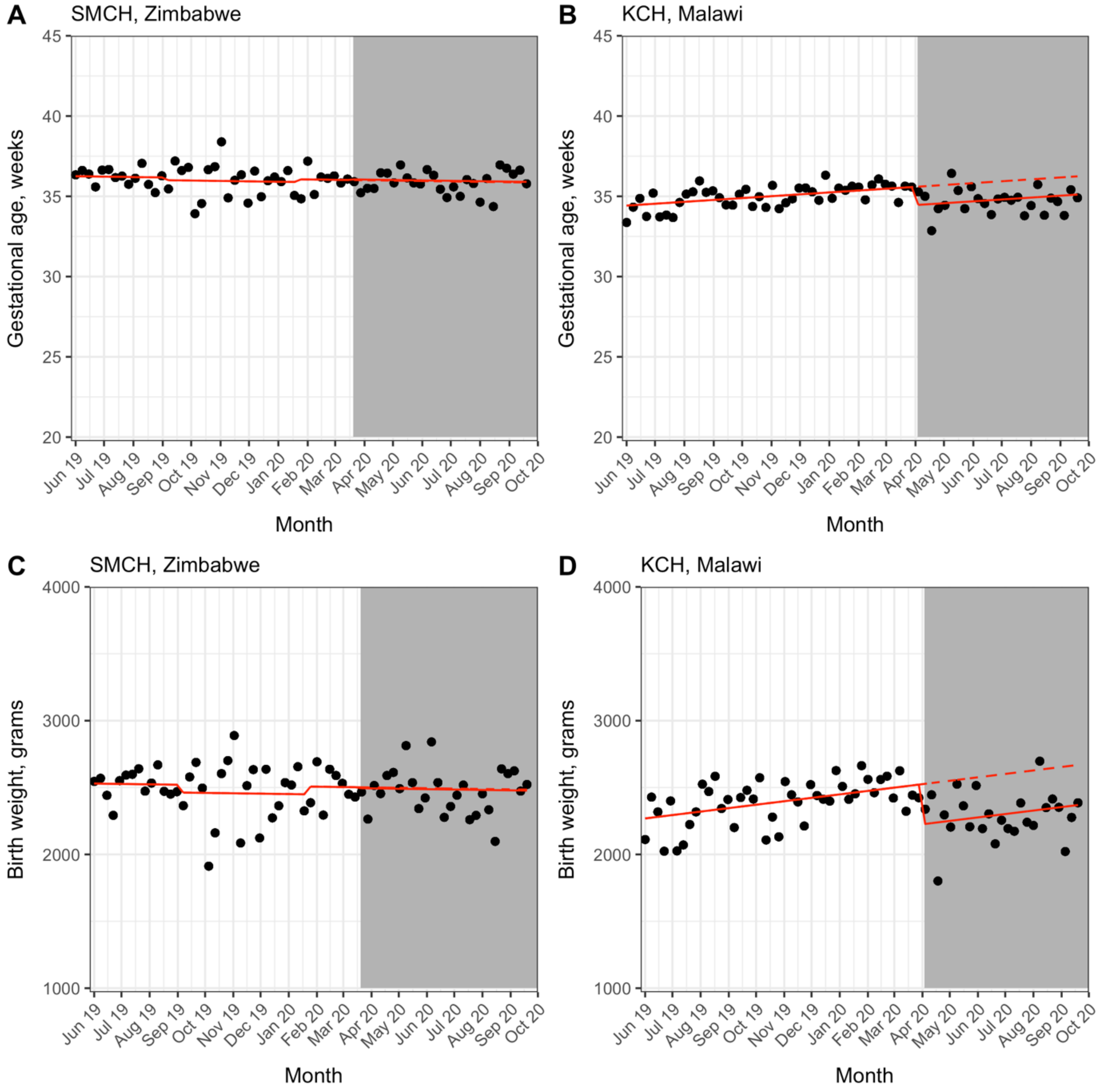

Supplementary Figure 1: Interrupted time series for gestational age and birth weight

- Data points represent weekly mean gestational age or birth weight to avoid overplotting.
- White background: pre-COVID-19 period; grey background: post-COVID-19 period.
- Solid line: predicted trend from regression model; dashed line: counterfactual scenario.
- SMCH models (panels A & C) adjusted for doctors' strike period, KCH models (panels B & D) unadjusted.
- Data from all admission forms completed, irrespective of match status.
- *SMCH: Sally Mugabe Central Hospital; KCH: Kamuzu Central Hospital*

For peer review only - http://bmjopen.bmj.com/site/about/guidelines.xhtml

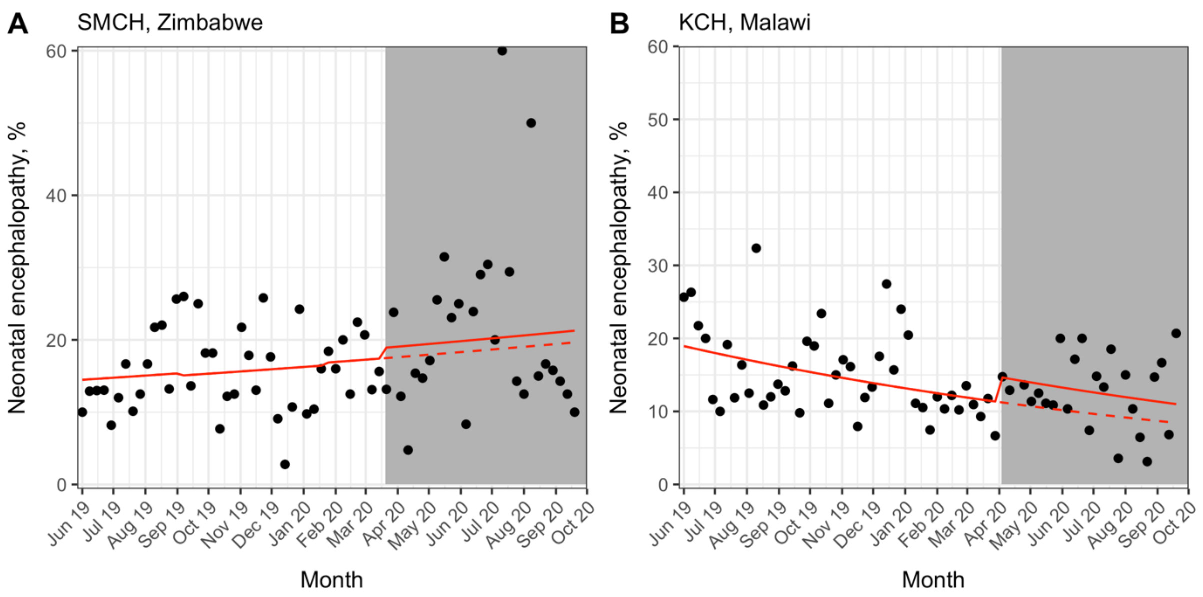

Supplementary Figure 2: Interrupted time series for prevalence of neonatal encephalopathy

- White background: pre-COVID-19 period; grey background: post-COVID-19 period.
- Solid line: predicted trend from regression model; dashed line: counterfactual scenario.
- SMCH model (panel A) adjusted for doctors' strike period, KCH model (panel B) unadjusted.
- Data from matched admission and outcome forms only.
- *SMCH: Sally Mugabe Central Hospital; KCH: Kamuzu Central Hospital*

For peer review only - http://bmjopen.bmj.com/site/about/guidelines.xhtml

APPENDIX 1: STROBE CHECKLIST

	Item No.	Recommendation	Page No.
**Title and abstract**	1	(*a*) Indicate the study's design with a commonly used term in the title or the abstract	1-2
		(*b*) Provide in the abstract an informative and balanced summary of what was done and what was found	2
**Introduction**			
Background/rationale	2	Explain the scientific background and rationale for the investigation being reported	6-7
Objectives	3	State specific objectives, including any prespecified hypotheses	7
**Methods**			
Study design	4	Present key elements of study design early in the paper	7-8
Setting	5	Describe the setting, locations, and relevant dates, including periods of recruitment, exposure, follow-up, and data collection	8
Participants	6	(*a*) *Cohort study*—Give the eligibility criteria, and the sources and methods of selection of participants. Describe methods of follow-up<br>*Case-control study*—Give the eligibility criteria, and the sources and methods of case ascertainment and control selection. Give the rationale for the choice of cases and controls<br>*Cross-sectional study*—Give the eligibility criteria, and the sources and methods of selection of participants	8
		(*b*) *Cohort study*—For matched studies, give matching criteria and number of exposed and unexposed<br>*Case-control study*—For matched studies, give matching criteria and the number of controls per case	
Variables	7	Clearly define all outcomes, exposures, predictors, potential confounders, and effect modifiers. Give diagnostic criteria, if applicable	9
Data sources/ measurement	8*	For each variable of interest, give sources of data and details of methods of assessment (measurement). Describe comparability of assessment methods if there is more than one group	9
Bias	9	Describe any efforts to address potential sources of bias	9-10
Study size	10	Explain how the study size was arrived at	10
Quantitative variables	11	Explain how quantitative variables were handled in the analyses. If applicable, describe which groupings were chosen and why	9-10
Statistical methods	12	(*a*) Describe all statistical methods, including those used to control for confounding	10-11
		(*b*) Describe any methods used to examine subgroups and interactions	10-11, Appendix 5, Appendix 6
		(*c*) Explain how missing data were addressed	10-11, Appendix 4

For peer review only

For peer review only - http://bmjopen.bmj.com/site/about/guidelines.xhtml

		(*d*) *Cohort study*—If applicable, explain how loss to follow-up was addressed<br>*Case-control study*—If applicable, explain how matching of cases and controls was addressed<br>*Cross-sectional study*—If applicable, describe analytical methods taking account of sampling strategy	10-11
		(*e*) Describe any sensitivity analyses	10-11, Appendices 5-6
**Results**			
Participants	13*	(a) Report numbers of individuals at each stage of study—eg numbers potentially eligible, examined for eligibility, confirmed eligible, included in the study, completing follow-up, and analysed	10,12, Appendix 3
		(b) Give reasons for non-participation at each stage	10, 12, Appendix 3
		(c) Consider use of a flow diagram	Appendix 3
Descriptive data	14*	(a) Give characteristics of study participants (eg demographic, clinical, social) and information on exposures and potential confounders	12-15, Appendix 5
		(b) Indicate number of participants with missing data for each variable of interest	Appendix 4
		(c) *Cohort study*—Summarise follow-up time (eg, average and total amount)	12-15
Outcome data	15*	*Cohort study*—Report numbers of outcome events or summary measures over time	12-15
		Case-control study—Report numbers in each exposure category, or summary measures of exposure	
		Cross-sectional study—Report numbers of outcome events or summary measures	
Main results	16	(*a*) Give unadjusted estimates and, if applicable, confounder-adjusted estimates and their precision (eg, 95% confidence interval). Make clear which confounders were adjusted for and why they were included	12-15
		(*b*) Report category boundaries when continuous variables were categorized	12-15
		(*c*) If relevant, consider translating estimates of relative risk into absolute risk for a meaningful time period	12-15
Other analyses	17	Report other analyses done—eg analyses of subgroups and interactions, and sensitivity analyses	Appendix 5, Appendix 6
**Discussion**			
Key results	18	Summarise key results with reference to study objectives	15
Limitations	19	Discuss limitations of the study, taking into account sources of potential bias or imprecision. Discuss both direction and magnitude of any potential bias	17
Interpretation	20	Give a cautious overall interpretation of results considering objectives, limitations, multiplicity of analyses, results from similar studies, and other relevant evidence	15-17
Generalisability	21	Discuss the generalisability (external validity) of the study results	15-18

For peer review only

**Other information**			
Funding	22	Give the source of funding and the role of the funders for the present study and, if applicable, for the original study on which the present article is based	24

Adapted from: von Elm E, Altman DG, Egger M, Pocock SJ, Gøtzsche PC, et al. (2007) The Strengthening the Reporting of Observational Studies in Epidemiology (STROBE) Statement: Guidelines for Reporting Observational Studies. PLOS Medicine 4(10): e296. https://doi.org/10.1371/journal.pmed.0040296

For peer review only

APPENDIX 2: ETHICAL APPROVAL

Ethical approval for this study was granted by the following ethics committees.

Committee	Reference
United Kingdom	
University College London Research Ethics Committee	17123/001
Malawi	
College of Medicine Research and Ethics Committee	P.01/20/2909
Zimbabwe	
Medical Research Council of Zimbabwe	MRCZ/A/2570
Joint Research Ethics Committee for the University of Zimbabwe, College of Health Sciences and Parirenyatwa Group of Hospitals	JREC/327/19
Biomedical Research and Training Institute Institutional Review Board	AP155/2020
Sally Mugabe (Harare) Central Hospital Ethics Committee	071119/64

For peer review only

For peer review only - http://bmjopen.bmj.com/site/about/guidelines.xhtml

APPENDIX 3: FLOW DIAGRAMS OF RECORD INCLUSION

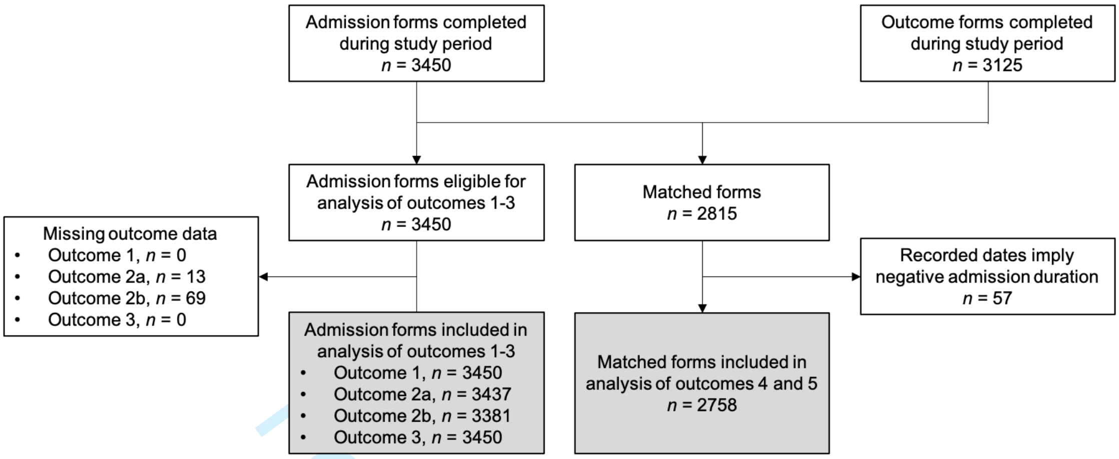

Flow diagram of record inclusion for analysis of data at Sally Mugabe Central Hospital, Zimbabwe

- Outcome 1: number of admissions
- Outcome 2a: gestational age
- Outcome 2b: birth weight
- Outcome 3: source of admission
- Outcome 4: prevalence of neonatal encephalopathy
- Outcome 5: overall mortality rate

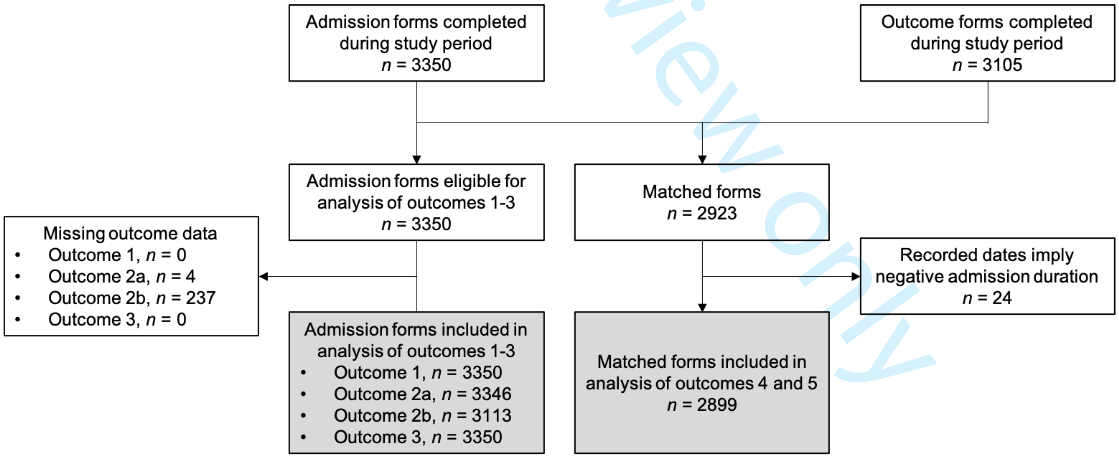

Flow diagram of record inclusion for analysis of data at Kamuzu Central Hospital, Malawi

- Outcome 1: number of admissions
- Outcome 2a: gestational age
- Outcome 2b: birth weight
- Outcome 3: source of admission
- Outcome 4: prevalence of neonatal encephalopathy
- Outcome 5: overall mortality rate

For peer review only - http://bmjopen.bmj.com/site/about/guidelines.xhtml

APPENDIX 4: MISSING DATA

The table below shows the number of participants with missing data for each outcome and the number of participants remaining for each analysis after pairwise deletion of missing values.

Characteristics	*n* missing (%)		*n* remaining*	
	SMCH	KCH	SMCH	KCH
Gestational age	13 (0·4)	4 (0·1)	3437 (99·6)	3346 (99·9)
Birth weight	69 (2·0)	237 (7·1)	3381 (98·0)	3113 (92·9)
Source of admission	0 (0·0)	0 (0·0)	3450 (100·0)	3350 (100·0)
Neonatal encephalopathy	0 (0·0)	0 (0·0)	2758 (100·0)†	2899 (100·0)†
Death	0 (0·0)	0 (0·0)	2758 (100·0)†	2899 (100·0)†

- * Remaining for analysis after pairwise deletion.
- † Only matched admission and outcome forms considered for analysis of neonatal encephalopathy and death.
- *SMCH: Sally Mugabe Central Hospital; KCH: Kamuzu Central Hospital, Malawi*

For peer review only - http://bmjopen.bmj.com/site/about/guidelines.xhtml

APPENDIX 5: FURTHER REGRESSION ANALYSIS RESULTS

Outcome 1: Admissions to the neonatal unit

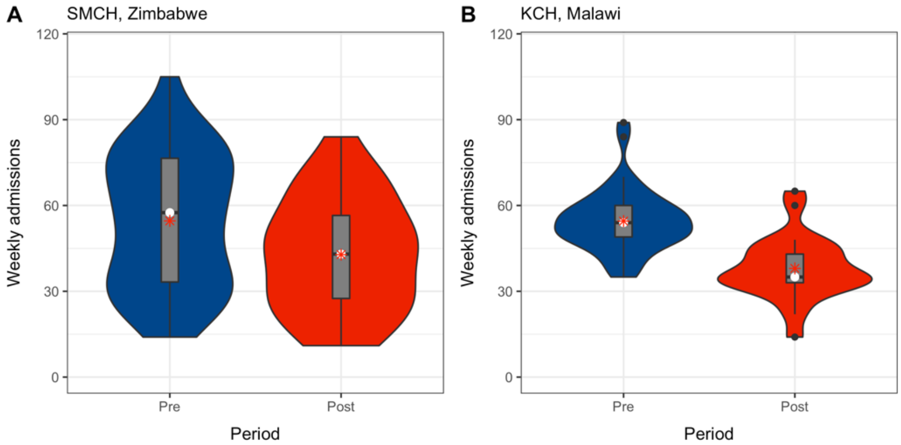

Distribution of weekly admissions by COVID-19 period

SMCH model 1: Level change model, adjusted for doctors' strike period

	Coef	SE	Exp	95% CI	*p*-value
Intercept	4·43	0·08	84·31	71·89 – 98·86	< 0·001
Post-COVID-19 period, yes	-0·19	0·17	0·83	0·60 – 1·14	0·25
Study time elapsed, weeks	-0·009	0·004	0·99	0·98 – 1·00	0·012
Doctors' strike period, yes	-0·68	0·11	0·51	0·41 – 0·63	< 0·001

SMCH model 2: Level change model, additionally adjusted for nurses' strike period

	Coef	SE	Exp	95% CI	*p*-value
Intercept	4·37	0·07	79·20	68·81 – 91·16	< 0·001
Post- COVID-19 period, yes	-0·11	0·14	0·90	0·69 – 1·17	0·90
Study time elapsed, weeks	-0·005	0·003	1·00	0·99 – 1·00	0·10
Doctors' strike period, yes	-0·70	0·09	0·50	0·41 – 0·60	< 0·001
Nurses' strike period, yes	-0·65	0·14	0·52	0·40 – 0·68	< 0·001

KCH model: Level change model, unadjusted

	Coef	SE	Exp	95% CI	*p*-value
Intercept	3·88	0·06	48·42	43·03 – 54·49	< 0·001
Post- COVID-19 period, yes	-0·55	0·10	0·58	0·48 – 0·70	< 0·001
Study time elapsed, weeks	0·005	0·002	1·01	1·00 – 1·01	0·019

For peer review only - http://bmjopen.bmj.com/site/about/guidelines.xhtml

Outcome 2: Gestational age at birth and birth weight

Gestational age at birth

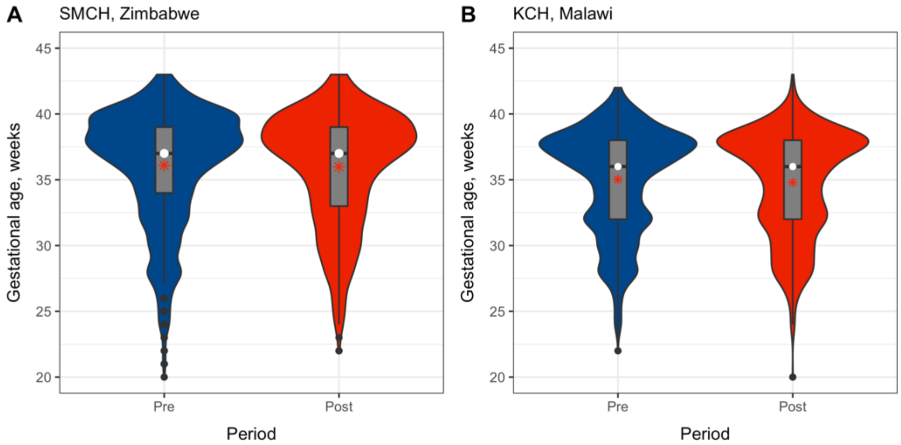

Distribution of gestational age at birth (weeks) by COVID-19 (pre/post-COVID19) period

SMCH model: Level change model, adjusted for doctors' strike period

	Coef	SE	95% CI	*p*-value
Intercept	36·25	0·15	35·96 – 36·54	< 0·001
Post-COVID-19 period, yes	0·04	0·29	-0·53 – 0·61	0·89
Study time elapsed, weeks	-0·006	0·006	-0·02 – 0·007	0·37
Doctors' strike period, yes	-0·17	0·20	-0·57 – 0·23	0·41

KCH model: Level change model, unadjusted

	Coef	SE	95% CI	*p*-value
Intercept	34·42	0·15	34·12 – 34·72	< 0·001
Post-COVID-19 period, yes	-1·14	0·25	-1·62 – -0·65	< 0·001
Study time elapsed, weeks	0·03	0·006	0·02 – 0·04	< 0·001

For peer review only - http://bmjopen.bmj.com/site/about/guidelines.xhtml

Birth weight

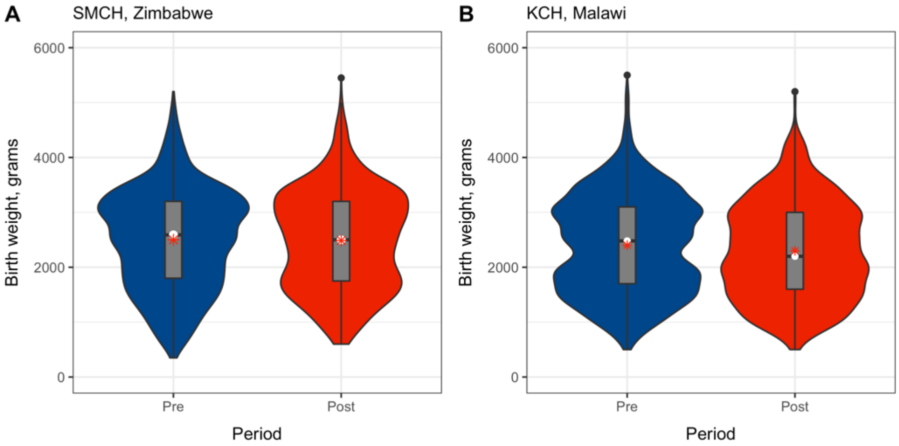

Distribution of birth weight (grams) by COVID-19 (pre/post-COVID19) period

For peer review only

SMCH model: Level change model, adjusted for doctors' strike period

	Coef	SE	95% CI	*p*-value
Intercept	2530·0	31·5	2468·0 – 2591·4	< 0·001
Post- COVID-19 period, yes	-7·2	61·1	-127·1 – 112·6	0·91
Study time elapsed, weeks	-0·7	1·3	-3·3 – 2·0	0·62
Doctors' strike period, yes	-58·1	42·9	-142·1 – 25·9	0·18

KCH model: Level change model, unadjusted

	Coef	SE	95% CI	*p*-value
Intercept	2269·0	36·0	2198·4 – 2339·6	< 0·001
Post- COVID-19 period, yes	-299·9	57·3	-412·3 – -187·5	< 0·001
Study time elapsed, weeks	5·9	1·4	3·2 – 8·6	< 0·001

Outcome 3: Source of admission referral

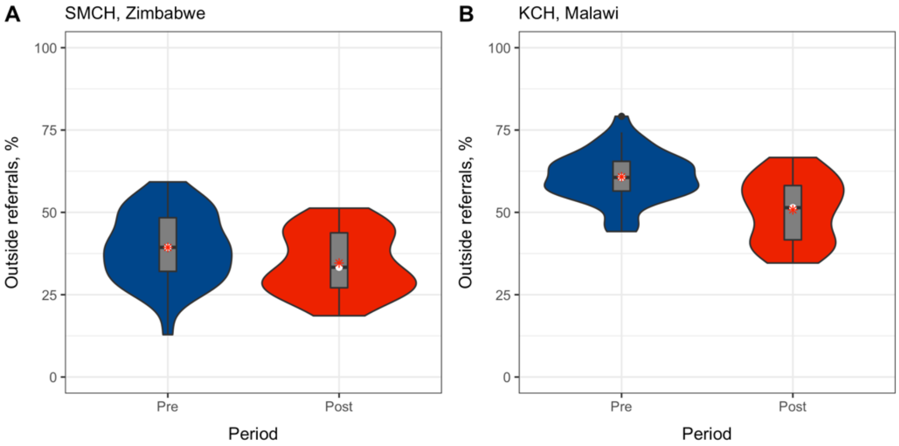

Distribution of outside referrals (%) by pre/post-COVID-19 period

SMCH model: Level change model, adjusted for doctors' strike period

	Coef	SE	Exp	95% CI	*p*-value
Intercept	-1·15	0·06	0·32	0·28 – 0·36	< 0·001
Post- COVID-19 period, yes	-0·02	0·11	0·98	0·79 – 1·23	0·88
Study time elapsed, weeks	0·001	0·003	1·00	1·00 – 1·01	0·55
Doctors' strike period, yes	0·33	0·07	1·39	1·20 – 1·60	< 0·001

KCH model: Level change model, unadjusted

	Coef	SE	Exp	95% CI	*p*-value
Intercept	-0·59	0·04	0·55	0·51 – 0·59	< 0·001
Post- COVID-19 period, yes	-0·33	0·06	0·72	0·65 – 0·81	< 0·001
Study time elapsed, weeks	0·005	0·001	1·01	1·00 – 1·01	0·001

For peer review only - http://bmjopen.bmj.com/site/about/guidelines.xhtml

Outcome 4: Prevalence of neonatal encephalopathy

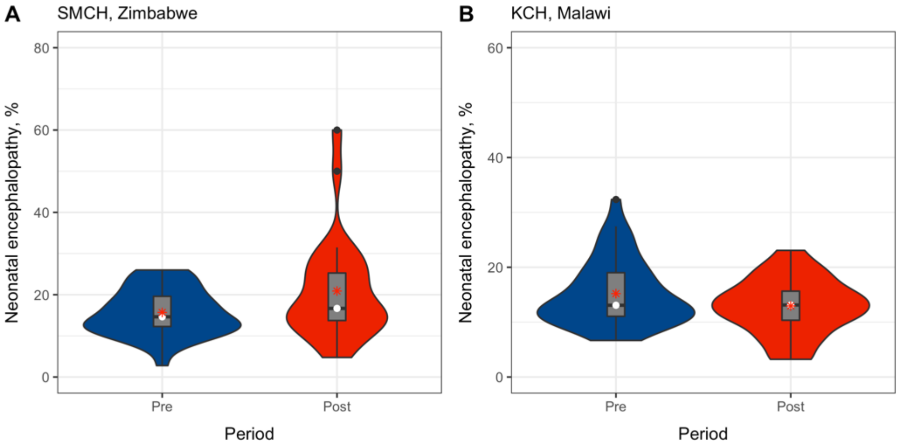

Distribution of neonatal encephalopathy (%) by pre/post-COVID-19 period

SMCH model: Level change model, adjusted for doctors' strike period

	Coef	SE	Exp	95% CI	*p*-value
Intercept	-1·93	0·10	0·15	0·12 – 0·18	< 0·001
Post- COVID-19 period, yes	0·08	0·18	1·08	0·76 – 1·55	0·67
Study time elapsed, weeks	0·004	0·004	1·00	1·00 – 1·01	0·27
Doctors' strike period, yes	-0·02	0·13	0·98	0·76 – 1·26	0·87

KCH model: Level change model, unadjusted

	Coef	SE	Exp	95% CI	*p*-value
Intercept	-1·66	0·09	0·19	0·16 – 0·23	< 0·001
Post- COVID-19 period, yes	0·27	0·16	1·30	0·95 – 1·80	0·11
Study time elapsed, weeks	-0·01	0·004	0·99	0·98 – 1·00	0·001

For peer review only - http://bmjopen.bmj.com/site/about/guidelines.xhtml

Outcome 5: Overall mortality

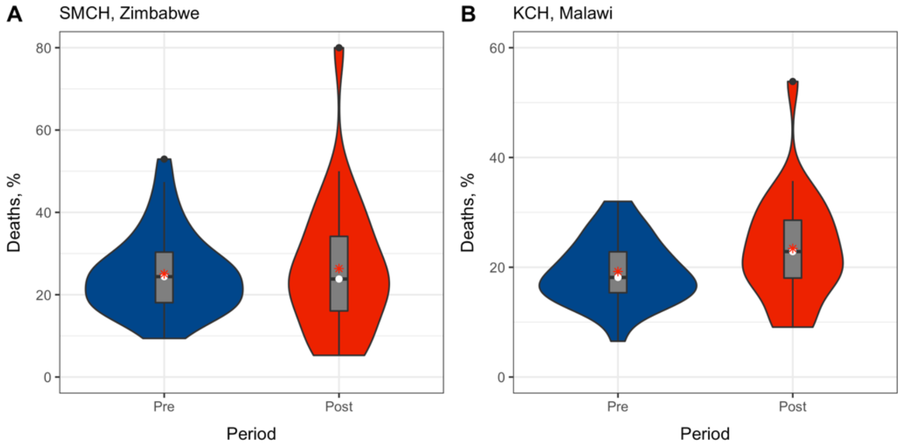

Distribution of overall mortality (%) by pre/post-COVID-19 period

SMCH model 1: Level change model, adjusted for doctors' strike period

	Coef	SE	Exp	95% CI	*p*-value
Intercept	-1·65	0·08	0·19	0·16 – 0·23	< 0·001
Post- COVID-19 period, yes	-0·22	0·16	0·80	0·56 – 1·15	0·23
Study time elapsed, weeks	0·007	0·003	1·01	1·00 – 1·02	0·09
Doctors' strike period, yes	0·17	0·10	1·19	0·94 – 1·50	0·16

SMCH model 2: Level change model, additionally adjusted for nurses' strike period

	Coef	SE	Exp	95% CI	*p*-value
Intercept	-1·60	0·09	0·20	0·17 – 0·24	< 0·001
Post- COVID-19 period, yes	-0·33	0·18	0·72	0·51 – 1·03	0·07
Study time elapsed, weeks	0·004	0·004	1·00	1·00 – 1·01	0·30
Doctors' strike period, yes	0·19	0·11	1·21	0·98 – 1·50	0·08
Nurses' strike period, yes	0·60	0·17	1·82	1·30 – 2·55	0·001

KCH model 1: Level change model, unadjusted

Mal – deaths (unadjusted)	Coef	SE	Exp	95% CI	*p*-value
Intercept	-1·56	0·09	0·21	0·18 – 0·25	< 0·001
Post- COVID-19 period, yes	0·27	0·14	1·31	0·98 – 1·73	0·07
Study time elapsed, weeks	-0·004	0·003	1·00	0·99 – 1·00	0·27

For peer review only - http://bmjopen.bmj.com/site/about/guidelines.xhtml

KCH model 2: Slope change model, unadjusted

**Mal – deaths (sensitivity)**	**Coef**	**SE**	**Exp**	**95% CI**	***p*-value**
Intercept	-1·55	0·09	0·21	0·18 – 0·25	< 0·001
Study time elapsed, weeks	-0·004	0·003	1·00	0·99 – 1·00	0·25
Time since first COVID-19 case, weeks * post- COVID-19 period, yes	0·02	0·009	1·02	1·00 – 1·04	0·04

For peer review only

APPENDIX 6: ADDITIONAL ANALYSES

Mode of delivery of admitted neonates

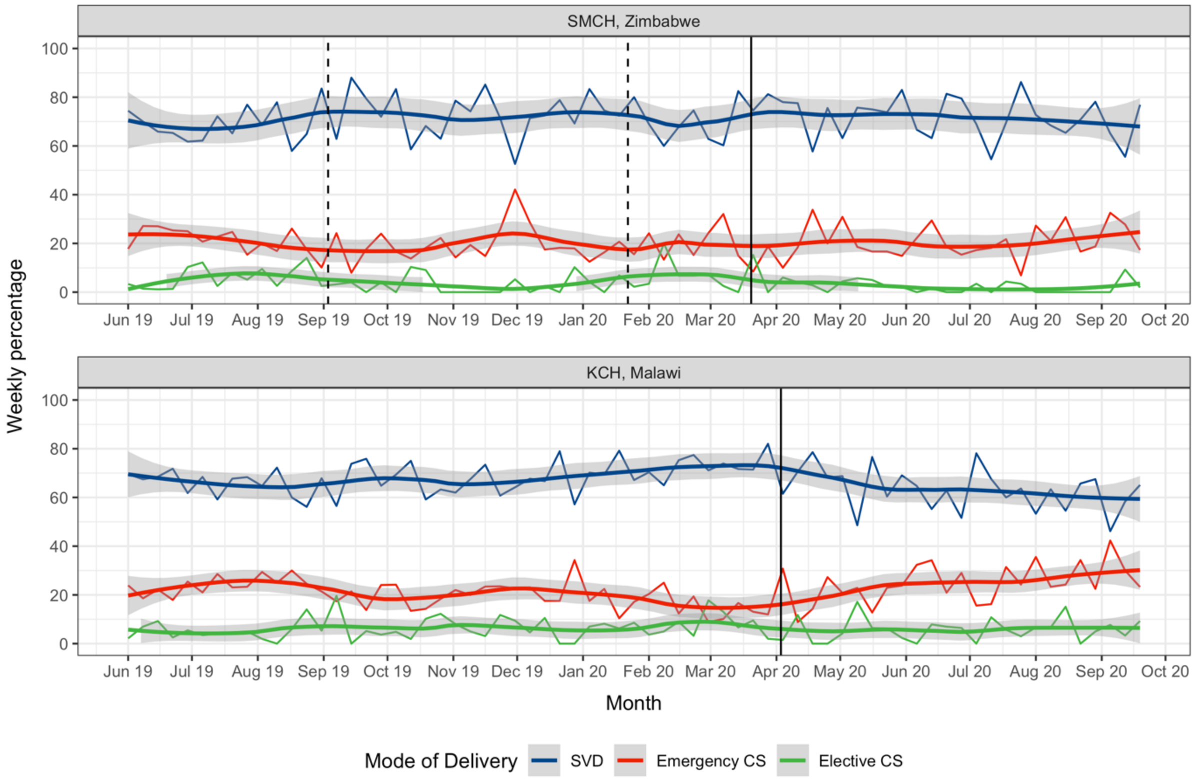

Trend in mode of delivery of admitted neonates per week

- Only SVD, emergency CS and elective CS displayed here to avoid overplotting.
- Smoothed line: local regression (LOESS) model; shaded region: 95% confidence interval.
- Solid vertical line: first confirmed case of COVID-19 in each country.
- Period between dashed vertical lines: industrial action by doctors in Zimbabwe.
- Counts based on all admission forms completed, irrespective of match status.
- *SMCH: Sally Mugabe Central Hospital; KCH: Kamuzu Central Hospital; SVD: spontaneous vaginal delivery; CS: caesarean section*

For peer review only - http://bmjopen.bmj.com/site/about/guidelines.xhtml

APPENDIX 1: STROBE CHECKLIST

	Item No.	Recommendation	Page No.
**Title and abstract**	1	(*a*) Indicate the study's design with a commonly used term in the title or the abstract	1-2
		(*b*) Provide in the abstract an informative and balanced summary of what was done and what was found	2
**Introduction**			
Background/rationale	2	Explain the scientific background and rationale for the investigation being reported	6-7
Objectives	3	State specific objectives, including any prespecified hypotheses	7
**Methods**			
Study design	4	Present key elements of study design early in the paper	7-8
Setting	5	Describe the setting, locations, and relevant dates, including periods of recruitment, exposure, follow-up, and data collection	8
Participants	6	(*a*) *Cohort study*—Give the eligibility criteria, and the sources and methods of selection of participants. Describe methods of follow-up<br>*Case-control study*—Give the eligibility criteria, and the sources and methods of case ascertainment and control selection. Give the rationale for the choice of cases and controls<br>*Cross-sectional study*—Give the eligibility criteria, and the sources and methods of selection of participants	8
		(*b*) *Cohort study*—For matched studies, give matching criteria and number of exposed and unexposed<br>*Case-control study*—For matched studies, give matching criteria and the number of controls per case	
Variables	7	Clearly define all outcomes, exposures, predictors, potential confounders, and effect modifiers. Give diagnostic criteria, if applicable	9
Data sources/ measurement	8*	For each variable of interest, give sources of data and details of methods of assessment (measurement). Describe comparability of assessment methods if there is more than one group	9
Bias	9	Describe any efforts to address potential sources of bias	9-10
Study size	10	Explain how the study size was arrived at	10
Quantitative variables	11	Explain how quantitative variables were handled in the analyses. If applicable, describe which groupings were chosen and why	9-10
Statistical methods	12	(*a*) Describe all statistical methods, including those used to control for confounding	10-11
		(*b*) Describe any methods used to examine subgroups and interactions	10-11, Appendix 5, Appendix 6
		(*c*) Explain how missing data were addressed	10-11, Appendix 4

For peer review only

		(*d*) *Cohort study*—If applicable, explain how loss to follow-up was addressed<br>*Case-control study*—If applicable, explain how matching of cases and controls was addressed<br>*Cross-sectional study*—If applicable, describe analytical methods taking account of sampling strategy	10-11
		(*e*) Describe any sensitivity analyses	10-11, Appendices 5-6
**Results**			
Participants	13*	(a) Report numbers of individuals at each stage of study—eg numbers potentially eligible, examined for eligibility, confirmed eligible, included in the study, completing follow-up, and analysed	10,12, Appendix 3
		(b) Give reasons for non-participation at each stage	10, 12, Appendix 3
		(c) Consider use of a flow diagram	Appendix 3
Descriptive data	14*	(a) Give characteristics of study participants (eg demographic, clinical, social) and information on exposures and potential confounders	12-15, Appendix 5
		(b) Indicate number of participants with missing data for each variable of interest	Appendix 4
		(c) *Cohort study*—Summarise follow-up time (eg, average and total amount)	12-15
Outcome data	15*	*Cohort study*—Report numbers of outcome events or summary measures over time	12-15
		Case-control study—Report numbers in each exposure category, or summary measures of exposure	
		Cross-sectional study—Report numbers of outcome events or summary measures	
Main results	16	(*a*) Give unadjusted estimates and, if applicable, confounder-adjusted estimates and their precision (eg, 95% confidence interval). Make clear which confounders were adjusted for and why they were included	12-15
		(*b*) Report category boundaries when continuous variables were categorized	12-15
		(*c*) If relevant, consider translating estimates of relative risk into absolute risk for a meaningful time period	12-15
Other analyses	17	Report other analyses done—eg analyses of subgroups and interactions, and sensitivity analyses	Appendix 5, Appendix 6
**Discussion**			
Key results	18	Summarise key results with reference to study objectives	15
Limitations	19	Discuss limitations of the study, taking into account sources of potential bias or imprecision. Discuss both direction and magnitude of any potential bias	17
Interpretation	20	Give a cautious overall interpretation of results considering objectives, limitations, multiplicity of analyses, results from similar studies, and other relevant evidence	15-17
Generalisability	21	Discuss the generalisability (external validity) of the study results	15-18

For peer review only

**Other information**			
Funding	22	Give the source of funding and the role of the funders for the present study and, if applicable, for the original study on which the present article is based	24

Adapted from: von Elm E, Altman DG, Egger M, Pocock SJ, Gøtzsche PC, et al. (2007) The Strengthening the Reporting of Observational Studies in Epidemiology (STROBE) Statement: Guidelines for Reporting Observational Studies. PLOS Medicine 4(10): e296. https://doi.org/10.1371/journal.pmed.004029

For peer review only

For peer review only

BMJ Open

Indirect impacts of the COVID-19 pandemic at two tertiary neonatal units in Zimbabwe and Malawi: an interrupted time series analysis

Journal:	*BMJ Open*
Manuscript ID	bmjopen-2021-048955.R1
Article Type:	Original research
Date Submitted by the Author:	02-Jul-2021
Complete List of Authors:	Chimhuya, Simbarashe; University of Zimbabwe<br>Neal, Samuel; University College London, Institute of Child Health<br>Chimhini, Gwen; University of Zimbabwe<br>Gannon, Hannah; UCL<br>Cortina Borja, Mario; University College London, Institute of Child Health<br>Crehan, Caroline; University College London, Institute of Child Health<br>Nkhoma, Deliwe<br>Chiyaka, Tarisai; Biomedical Research and Training Institute<br>Wilson, Emma; University College London, Institute of Child Health<br>Hull-Bailey, Tim; University College London, Institute of Child Health<br>Fitzgerald, Felicity<br>Chiume, Msandeni; Kamuzu Central Hospital, Department of Pediatrics<br>Heys, Michelle; University College London Institute of Child Health, University College London Institute of Child Health
**Primary Subject Heading**:	Global health
Secondary Subject Heading:	Paediatrics, Public health
Keywords:	COVID-19, PAEDIATRICS, Neonatal intensive & critical care < INTENSIVE & CRITICAL CARE, Quality in health care < HEALTH SERVICES ADMINISTRATION & MANAGEMENT, International health services < HEALTH SERVICES ADMINISTRATION & MANAGEMENT

Note: The following files were submitted by the author for peer review, but cannot be converted to PDF. You must view these files (e.g. movies) online.

figure2.eps
figure3.eps
figure4.eps

SCHOLARONE™
Manuscripts

I, the Submitting Author has the right to grant and does grant on behalf of all authors of the Work (as defined in the below author licence), an exclusive licence and/or a non-exclusive licence for contributions from authors who are: i) UK Crown employees; ii) where BMJ has agreed a CC-BY licence shall apply, and/or iii) in accordance with the terms applicable for US Federal Government officers or employees acting as part of their official duties; on a worldwide, perpetual, irrevocable, royalty-free basis to BMJ Publishing Group Ltd ("BMJ") its licensees and where the relevant Journal is co-owned by BMJ to the co-owners of the Journal, to publish the Work in this journal and any other BMJ products and to exploit all rights, as set out in our licence.

The Submitting Author accepts and understands that any supply made under these terms is made by BMJ to the Submitting Author unless you are acting as an employee on behalf of your employer or a postgraduate student of an affiliated institution which is paying any applicable article publishing charge ("APC") for Open Access articles. Where the Submitting Author wishes to make the Work available on an Open Access basis (and intends to pay the relevant APC), the terms of reuse of such Open Access shall be governed by a Creative Commons licence – details of these licences and which Creative Commons licence will apply to this Work are set out in our licence referred to above.

Other than as permitted in any relevant BMJ Author's Self Archiving Policies, I confirm this Work has not been accepted for publication elsewhere, is not being considered for publication elsewhere and does not duplicate material already published. I confirm all authors consent to publication of this Work and authorise the granting of this licence.

For peer review only

Indirect impacts of the COVID-19 pandemic at two tertiary neonatal units in Zimbabwe and Malawi: an interrupted time series analysis

Simbarashe Chimhuya, MMED*[1], Samuel R. Neal, MRes*[2], Gwendoline Chimhini, MMED[1], Hannah Gannon, MBChB[2], Mario Cortina-Borja, PhD[2], Caroline Crehan, MSc[2], Deliwe Nkhoma, MSc[3], Tarisai Chiyaka, MSc[4], Emma Wilson, PhD[2], Tim Hull-Bailey, MPhil[2], Felicity Fitzgerald, PhD[5], Msandeni Chiume, MBBS ‡[6], and Michelle Heys, MD(Res)‡ † [2]

1. Child and Adolescent Health Unit, Faculty of Medicine and Health Sciences, University of Zimbabwe, Harare, Zimbabwe
2. Population, Policy and Practice Research and Teaching Department, UCL Great Ormond Street Institute of Child Health, University College London, UK
3. Parent and Child Health Initiative Trust, Lilongwe, Malawi
4. Biomedical Research and Training Institute, Harare, Zimbabwe
5. Infection, Immunity and Inflammation, UCL Great Ormond Street Institute of Child Health, University College London, UK
6. Department of Paediatrics, Kamuzu Central Hospital, Lilongwe, Malawi

*Contributed equally as first author
†Corresponding author
‡ Contributed equally as last author

Correspondence to:
Dr Michelle Heys
Population, Policy and Practice Department,
UCL Great Ormond Street Institute of Child Health,
30 Guilford Street,
London, WC1N 1EH
Email: m.heys@ucl.ac.uk
Telephone: +44 (0)20 7905 2212

For peer review only

Word count: 3432

Keywords: COVID-19, neonatal, NICU, low-resource settings, global health

Contributors' statement

Concept and study design by SC, SRN, GC, FF, MCB, CC, MC and MH with input from other authors. Data collected by HG, DN, TC, CC and THB. Analysis performed by SRN and MCB with contributions from FF, SC, EW & MH. Manuscript drafted by SC and SRN with input from GC, FF, MCB, MC & MH. All authors proof-read and approved final draft. Underlying data accessed and verified by SRN, MCB, HG, FF & MH.

ABSTRACT

Objectives: To examine indirect impacts of the COVID-19 pandemic on neonatal care in low-income and middle-income countries.

Design: Interrupted time series analysis.

Setting: Two tertiary neonatal units in Harare, Zimbabwe and Lilongwe, Malawi.

Participants: We included a total of 6,800 neonates who were admitted to either neonatal unit from 1 June 2019 to 25 September 2020 (Zimbabwe: 3,450; Malawi: 3,350). We applied no specific exclusion criteria.

Interventions: The first cases of COVID-19 in each country (Zimbabwe: 20 March 2020; Malawi: 3 April 2020).

Primary outcome measures: Changes in the number of admissions, gestational age and birth weight, source of admission referrals, prevalence of neonatal encephalopathy, and overall mortality before and after the first cases of COVID-19.

Results: Admission numbers in Zimbabwe did not initially change after the first case of COVID-19 but fell by 48% during a nurses' strike (relative risk (RR) 0·52, 95% CI 0·40-0·68, *p* < 0·001). In Malawi, admissions dropped by 42% soon after the first case of COVID-19 (RR 0·58, 95% CI 0·48-0·70, *p* < 0·001). In Malawi, gestational age and birth weight decreased slightly by around 1 week (beta -1·14, 95% CI -1·62-[-]0·65, *p* < 0·001) and 300 grams (beta -299·9, 95% CI -412·3-[-]187·5, *p* < 0·001), outside referrals dropped by 28% (RR 0·72, 95% CI 0·65-0·81, *p* < 0·001), and there was a slight weekly increase in mortality (RR 1·02 per week, 95% CI 1·00-1·04, *p* = 0·04). No changes in these outcomes were found in Zimbabwe and no changes in the prevalence of neonatal encephalopathy were found at either site (*p* > 0.05).

Conclusions: The indirect impacts of COVID-19 are context-specific. While our study provides vital evidence to inform health providers and policy makers, national data are required to ascertain the true impacts of the pandemic on newborn health.

For peer review only - http://bmjopen.bmj.com/site/about/guidelines.xhtml

Strengths and limitations of this study

- We address the need for increased research into the indirect impacts of the COVID-19 pandemic on neonatal care in low-income and middle-income countries.
- We collected data digitally and in real time using the NeoTree application, which enabled a large sample size of 6800 neonates with minimal missing data.
- It is possible that unobserved events occurred close to the first case of COVID-19 in either country, which could have influenced our results
- We only collected data on neonates admitted to the neonatal unit and did not capture stillbirths or neonatal deaths that occurred in the community.

For peer review only

For peer review only - http://bmjopen.bmj.com/site/about/guidelines.xhtml

INTRODUCTION

The World Health Organization declared coronavirus disease (COVID-19) a Public Health Emergency of International Concern on 30 January 2020.[1] As of 21 October 2020, confirmed cases have exceeded 80 million globally with nearly 2 million deaths.[2] Zimbabwe recorded its first case on 20 March and, up to 21 October 2020,has reported over 17000 cases with more than 400 deaths.[2] Malawi confirmed its first three cases on 3 April and has reported more than 7000 cases and around 200 deaths over this same period.[2]

Before the COVID-19 pandemic, considerable improvements were made in global child health: the global neonatal mortality rate fell from 31 to 18 deaths per 1,000 live births between 2000 and 2018.[3] Yet there were disparities in the rates of decline with the sub-Saharan Africa region facing highest neonatal mortality rates[3]. Now, there is a danger that health outcomes in low-income and middle-income countries (LMICs) will fall further behind high-income countries. While countries worldwide face challenges related to the COVID-19 pandemic, LMICs are particularly struggling with financial constraints, limited testing capacity, lack of personal protective equipment, and staff shortages.[4,5] As children are at low-risk of infection or severe disease from COVID-19,[6-10] any impacts on their health outcomes will likely be attributable to the indirect effects of the pandemic on health systems, as in previous disease outbreaks.[11,12] These include increased rates of parental unemployment, food and housing insecurity, and reduced access to routine care, including antenatal and perinatal care, with potentially damaging downstream impacts on neonatal outcomes.[13,14]

For peer review only

The NeoTree application (app) is an Android tablet-based quality improvement platform that aims to reduce neonatal mortality in low-resource settings.[15] Developed in collaboration with local stakeholders, it is embedded in routine practice at two neonatal units (NNUs) in Zimbabwe and Malawi, providing real-time clinical decision support, neonatal care education, and digital data capture.[16 17]

We hypothesised that the COVID-19 pandemic would negatively impact care seeking behaviours, neonatal care provision and, ultimately, neonatal outcomes in LMICs. To test this hypothesis, we aimed to examine trends in markers of neonatal care before and during the COVID-19 pandemic at Sally Mugabe Central Hospital (SMCH), Zimbabwe, and Kamuzu Central Hospital (KCH), Malawi. Specifically, we compared the:

1. number of admissions to the NNU,
2. gestational age and birth weight of admitted neonates,
3. source of admission referrals,
4. prevalence of neonatal encephalopathy (NE), and
5. overall mortality rate

before and after the first reported cases of COVID-19.

For peer review only - http://bmjopen.bmj.com/site/about/guidelines.xhtml

METHODS

This study is reported in accordance with the Strengthening the Reporting of Observational Studies in Epidemiology (STROBE) statement (Appendix 1).

Setting

SMCH is a public referral hospital in Harare, Zimbabwe. It has the largest of three tertiary NNUs nationwide with 100 cots. KCH, Lilongwe, is one of four regional referral hospitals in Malawi and the NNU has 75 cots. Neonatal care at SMCH is predominantly doctor led while neonatal care at KCH is mostly nurse led. Both units accept local and national referrals for specialist surgical care.

In response to the COVID-19 pandemic, Zimbabwe and Malawi have both implemented response measures in an attempt to control the outbreak. In Zimbabwe, the Government closed borders to non-essential travel within days of the first in-country confirmed case of COVID-19 and imposed a full national lockdown that lasted from 30 March 2020 to 11 June 2020, which was followed by phased relaxations of the restrictions.[18] In Malawi, public events were banned and public gatherings restricted to fewer than 100 people on 20 March 2020, with all educational institutions closed several days later.[19] Borders were closed to non-essential travel on 1 April 2020 and a full national lockdown was announced to last for 21 days from 18 April 2020; however, a High Court injunction prevented this. Further restrictions were announced on 9 August 2020, mandating the wearing of face masks in public, closing places of worship, restaurants, and bars, and restricting public gatherings to less than 10 people initially, although these were revised within days to reallow gatherings up to 100 people.[20] Schools in Malawi reopened on 7 September 2020.[19]

For peer review only - http://bmjopen.bmj.com/site/about/guidelines.xhtml

Participants

All neonates admitted to each NNU over a 16-month period from 1 June 2019 to 25 September 2020 (69 complete weeks) were eligible for inclusion. We applied no specific exclusion criteria.

Data collection

Data were collected prospectively using the NeoTree app. Health workers complete a digital form when a neonate is admitted to the unit (admission form) and when they are discharged or die (outcome form). The app guides assessment of the neonate and collects data on patient demographics, examination findings, diagnoses, and interventions. Pseudonymised forms are uploaded monthly to University College London servers (Zimbabwe data) and Amazon Web Services (Malawi data). Admission and outcome forms are linked by a unique identifier generated by the app at admission.

Outcomes

We evaluated five outcomes:

1. Number of admissions: determined from the admission date of each completed admission form.
2. Gestational age at birth (weeks) and birth weight (grams): as entered into the admission form from obstetric records.
3. Source of admission: defined as 'within' (labour ward, postnatal ward, antenatal ward, obstetric theatre, or fee-paying ward [KCH only]) or 'outside' (referral from another health facility or postnatal self-referral from home).

For peer review only - http://bmjopen.bmj.com/site/about/guidelines.xhtml

4. Diagnosis of NE: defined as "hypoxic ischaemic encephalopathy" or "birth asphyxia" recorded as a diagnosis, cause of death or contributory cause of death on the outcome form.
5. Mortality: defined as an outcome of "neonatal death" on the outcome form. All other neonates, including those discharged, transferred to another facility or who left on parental request, were considered alive.

Ethical approval

Research ethics approval was granted by the UCL Research Ethics Committee (17123/001) and ethics committees in Malawi (P.01/20/2909) and Zimbabwe (MRCZ/A/2570) (Appendix 2). The need to obtain informed consent was waived as we collected only pseudonymised data routinely documented for clinical care.

Statistical analysis

Analyses were performed in R version 3.6.3,[21] running on RStudio version 1.2.5033.[22] First, admission forms were matched with their corresponding outcome form based on the unique identifier generated at admission. Lack of completed outcome forms (SMCH: *n*=316[9.1%]; KCH: *n*=243[7.2%]) or errors in entry of the unique identifier at discharge (SMCH: *n*=318[9.2%]; KCH: *n*=182[5.4%]) meant we were unable to match some admission forms with outcome forms (SMCH: *n*=634[18.3%]; KCH: *n*=425[12.6%]). For outcomes 1-3, we based analyses on data from all admission forms, regardless of match status. For outcomes 4 and 5, we based analyses on matched records only. Matched records implying a negative admission duration (i.e. outcome date prior to admission date) were excluded (SMCH: *n*=58[2%]; KCH: *n*=25[1%]). See Appendix 3 for a flow diagram of record inclusion. Missing data were

For peer review only

excluded using pairwise deletion for each analysis as frequencies of missing values were minimal (Appendix 4).

This study used an interrupted time series design with weekly data windows. We considered the first confirmed case of COVID-19 in each country as the intervention (Zimbabwe: 20 March 2020; Malawi: 3 April 2020).[2] For all outcomes, we hypothesised a level change impact model without a lag, and this was tested using interrupted time series regression models (see Bernal et al.[23]). Gestational age and birth weight were modelled with linear regression. All other outcomes were modelled using quasi-Poisson regression to account for overdispersion,[24] with the logarithm of the number of admissions in each weekly window included as an offset. All modles for SMCH were adjusted for a period of doctors' strikes from 3 September 2019 to 22 January 2020.[25] Models for KCH were unadjusted. Additional models were constructed to explore the effects of a nurses' strike in Zimbabwe (17 June to 9 September 2020)[26] and alternative impact models. Goodness-of-fit for nested models was compared with the *F*-test. See Appendix 5 for model details.

Patient and Public Involvement

Although patients and the public were not directly involved in this study, within the broader NeoTree co-development project we are carrying out a series of workshops and focus group discussions with healthcare workers and parents of admitted babies to ensure local ownership and relevance of this digital quality involvement tool aimed at improving healthcare outcomes for vulnerable neonates.

For peer review only

Role of the funding source

The funders had no role in study design, data collection, data analysis, data interpretation, or preparation of this manuscript.

For peer review only

For peer review only - http://bmjopen.bmj.com/site/about/guidelines.xhtml

RESULTS

Outcome 1: Admissions to the neonatal unit

We included 3,450 neonates at SMCH and 3,350 neonates at KCH. Figure 1 shows the seven-day moving average of admissions to the NNU.

At SMCH, the mean (SD) number of weekly admissions was 54·6 (23·5) before the first case of COVID-19 (pre-COVID-19) and 42·8 (19·9) afterwards (post-COVID-19). The level change regression model, adjusted for the doctors' strike, showed no evidence of a change in admissions after the first case of COVID-19 (relative risk [RR] 0·83; 95% confidence interval [CI] 0·60-1·14; p = 0·25) but the scatterplot indicated this model fit the data poorly (model 1, Figure 2A). An alternative model, additionally adjusted for the nurses' strike, again showed no change in the overall post-COVID-19 period (RR 0·90; 95%CI 0·69-1·17; p = 0·43) (model 2, Figure 2B). However, this model suggested that admissions fell by 48% during the nurses' strike period (RR 0·52, 95%CI 0·40-0·68, p < 0·001) and fit the data better (F[1, 64] = 24·66, p < 0·001).

At KCH, the mean (SD) number of weekly admissions was 54·5 (10·8) in the pre-COVID-19 period and 38·0 (10·9) in the post-COVID-19 period. The level change model suggested a 42% reduction in admissions after the first case of COVID-19 (RR 0·58; 95%CI 0·48-0·70; p < 0·001) (Figure 2C).

For peer review only - http://bmjopen.bmj.com/site/about/guidelines.xhtml

Outcome 2: Gestational age and birth weight

At SMCH, the mean (SD) gestational age at birth was 36·1 (4·4) weeks in the pre-COVID-19 period and 36·0 (4·2) weeks in the post-COVID-19 period. The mean (SD) birth weight was 2500 (908) grams in the pre-COVID-19 period and 2487 (896) grams in the post-COVID-19 period. Regression analysis indicated no change in gestational age at birth nor birth weight after the first case of COVID-19 (gestational age: beta 0·04; 95%CI -0·53-0·61; p = 0·89, birth weight: beta -7·2; 95%CI -127·1-112·6; p = 0·91) (Supplementary Figure 1A, Supplementary Figure 1C,). Adjusting for the nurses' strike did not improve model fit (data not shown).

At KCH, the mean (SD) gestational age was 35·0 (3·9) weeks in the pre-COVID-19 period and 34·8 (3·9) weeks in the post-COVID-19 period. The mean (SD) birth weight was 2402 (883) grams in the pre-COVID-19 period and 2299 (870) grams in the post-COVID-19 period. Gestational age decreased by one week in the post-COVID-19 period (beta -1·14; 95%CI -1·62-[-]0·65; p < 0·001) (Supplementary Figure 1B) and birth weight decreased by 300 grams (beta -299·9; 95%CI -412·3-[-]187·5; p < 0·001) (Supplementary Figure 1D).

Outcome 3: Source of admission referral

At SMCH, the mean (SD) percentage of outside referrals to the NNU was 39(11)% in the pre-COVID-19 period and 35(9)% in the post-COVID-19 period. The regression model showed no evidence of a change in the percentage of outside referrals after the first case of COVID-19 (RR 0·98; 95%CI 0·79-1·23; p = 0·88) (Figure 3A). Adjusting for the nurses' strike did not improve model fit (data not shown).

For peer review only - http://bmjopen.bmj.com/site/about/guidelines.xhtml

At KCH, the mean (SD) percentage of outside referrals was 61(8)% in the pre-COVID-19 period and 51(10)% in the post-COVID-19 period. Regression analysis suggested a 28% relative reduction in outside referrals after the first case of COVID-19 (RR 0·72; 95%CI 0·65-0·81; p < 0·001) (Figure 3B).

Outcome 4: Prevalence of neonatal encephalopathy

At SMCH, the mean (SD) percentage of admitted neonates diagnosed with NE was 16(6)% in the pre-COVID-19 period and 21(12)% in the post-COVID-19 period suggesting a possible increase. Regression analysis showed no statistically significant change in the percentage of neonates diagnosed with NE post-COVID-19 (RR 1·08; 95%CI 0·76-1·55; p = 0·67) (Supplementary Figure 2A). Adjusting for the nurses' strike did not improve model fit (data not shown).

At KCH, the mean (SD) percentage of admitted neonates diagnosed with NE was 15(6)% in the pre-COVID-19 period and 13(5)% in the post-COVID-19 period. The regression model suggested a possible increase in diagnoses of NE after the first case of COVID-19, but this was not statistically significant (RR 1·30; 95%CI 0·95-1·80; p = 0·11) (Supplementary Figure 2B).

Outcome 5: Overall mortality

For SMCH, the mean (SD) percentage of deaths per week of admission was 25(10)% in the pre-COVID-19 period and 26(16)% in the post-COVID-19 period. The level change regression model, adjusted for the doctors' strike, showed no evidence of a

For peer review only - http://bmjopen.bmj.com/site/about/guidelines.xhtml

change in mortality after the first case of COVID-19 (RR 0·80; 95%CI 0·56-1·15; *p* = 0·23) but the scatterplot indicated this model fit the data poorly (model 1, Figure 4A). An alternative model, additionally adjusted for the nurses' strike, again showed no change in overall mortality (RR 0·72; 95%CI 0·51-1·03; *p* = 0·07) but fit the data better (F[1, 64] = 11·61, *p* = 0·001) (model 2, Figure 4B).

For KCH, the mean (SD) percentage of deaths per week of admission was 19(6)% in the pre-COVID-19 period and 23(9)% in the post-COVID-19 period. The level change regression model suggested a possible increase in mortality after the first case of COVID-19, but this was not statistically significant (RR 1·31; 95%CI 0·98-1·73; *p* = 0·07) (Figure 4C). However, fitting a slope change impact model suggested a small relative increase in mortality by 2% per week in the post-COVID-19 period (RR 1·02 per week; 95%CI 1·00-1·04, *p* = 0·04) (Figure 4D).

For peer review only

For peer review only - http://bmjopen.bmj.com/site/about/guidelines.xhtml

DISCUSSION

Summary

We performed an interrupted time series analysis to examine changes in neonatal care provision at two tertiary NNUs in Zimbabwe and Malawi after the first cases of COVID-19 in each country. We found that admissions at SMCH did not change significantly after the first case of COVID-19 when considering this period as a whole, but there was a considerable decrease (around 50%) in the number admissions in June to August 2020, coinciding with a nurses' strike. We did not find significant changes in gestational age or birth weight, source of admission referrals, prevalence of NE or mortality at SMCH. Conversely, we found several changes in markers of neonatal care at KCH after the first case of COVID-19 in Malawi. The number of admissions fell by 42% and we noted a decrease in the gestational age and birth weight of admitted neonates (by around 1 week and 300 grams, respectively), a 28% relative decrease in outside referrals, and a small but statistically significant weekly increase in mortality by 2% after the first case of COVID-19. Although this study is descriptive, we can speculate about explanations for our results based on existing literature and discussions with local health workers.

Interpretation

The number of admissions at SMCH fell by around 50% between June to August 2020, but we noted no change outside this strike period, suggesting some resilience to the impact of the pandemic. However, nurses went on strike over pay and availability of personal protective equipment,[26] so the strike is itself an indirect consequence of COVID-19. A similar reduction in admissions was seen at KCH, but, unlike at SMCH, this 42% decrease was noted within a week of the first case of COVID-19. In Figure

For peer review only - http://bmjopen.bmj.com/site/about/guidelines.xhtml

5, we propose several interlinked factors that might explain reduced admissions to the NNU. Several of these factors, such as fear of using health services, disrupted transport networks and staff shortages have been directly reported by local sources in low-resource settings and were highlighted in a recent report by Graham et al.[27]

We found a slight decrease in gestational age and birth weight of neonates at KCH, but not SMCH. Studies have reported increased rates of preterm birth in pregnant women with COVID-19 compared to those without the disease, mostly from medically-induced preterm birth; although none of these studies were conducted in LMICs.[28] Preliminary analysis suggests rates of emergency caesarean section increased at SMCH and KCH, with a more marked increase at KCH (Appendix 6). This is one potential explanation for our findings. However, we noted that the number of outside referrals decreased by 28% at KCH, and neonates referred from outside KCH are more likely to be from lower-risk pregnancies that delivered in a health centre with higher gestational ages and birth weights. Further analysis should stratify by source of admission referral to clarify this finding, but is supported by the fact that referrals were rigorously triaged by the on-call paediatrician during the pandemic, and that referrals from some areas were diverted away from KCH.

We hypothesised that rates of NE would increase during the pandemic. NE is the clinical manifestation of disordered brain function and can have multiple aetiologies.[29] The term 'hypoxic-ischaemic encephalopathy' is reserved for cases where there is evidence of intrapartum asphyxia.[29] In LMICs, obstructed labour is a major cause of maternal mortality and can lead to intrapartum asphyxia with subsequent neonatal morbidity and mortality, including NE.[30] Therefore, the prevalence of NE might be

For peer review only - http://bmjopen.bmj.com/site/about/guidelines.xhtml

expected to increase as a marker of delayed presentation to a health facility. It is reassuring that we did not find increased rates of NE at SMCH or KCH. However, these findings should be interpreted cautiously as some neonates with NE may not have presented to a health facility at all.

Finally, we observed a slight increase in overall mortality at KCH (a relative increase of 2% per week after the first case of COVID-19), although not at SMCH. In KCH, the increase in mortality may be due to decreased gestational age and birthweight, but also due to a reduced rota of nursing staff implemented to protect healthcare workers. In fact, there was a suggestion that mortality decreased after the first case of COVID-19 in Zimbabwe, but this was not statistically significant. The reasons for this are unclear but could include factors such as increased stillbirth rates or improved care for the smaller number of neonates on the NNU. More complete analysis of facility-based and community-based neonatal mortality is greatly needed.

Limitations and future work

Some limitations should be noted. A limitation intrinsic to interrupted time series analysis is the possibility that another event occurred close to the first case of COVID-19 in either country causing spurious observations. Another potential threat to validity is changing data collection practices. For example, overstretched clinicians might not input data into the NeoTree app for all admitted neonates. However, this is unlikely as the NeoTree app is embedded into routine practice at SMCH and KCH and discussions with local collaborators suggest use of the app has continued without issue. At present, there is limited guidance on power and sample size calculations for interrupted time series analyses.[31] Therefore, we did not perform specific power

For peer review only

calculations and relied on the data available at the time of analysis. Our results suggest that our study has relatively low power to detect true changes in some outcomes, particularly NE, so these results should be interpreted cautiously in the absence of further data. Finally, the presence of seasonality is an important consideration in time series analyses. Unfortunately, prior to 2019, robust data for our outcomes are not available at either hospital due to a reliance on paper records, which could be lost or destroyed. Therefore, we could not adequately analyse seasonal patterns. However, for some outcomes, the scatterplots presented in our paper demonstrate a sudden shift in the trend at a defined time point in the series (around the first confirmed cases of COVID-19 or around time points coinciding with periods of industrial action). As similarly pronounced changes are not seen at other time points in the series, this would indicate the impact of the intervention despite any potential underlying seasonality.

The NeoTree app only collects data on neonates admitted to the NNU. Therefore, our analysis does not capture stillbirths or neonatal deaths that occur in the community. It is troubling to see a dramatic fall in admissions in both sites, raising the possibility that many unwell neonates did not attend a health facility and died at home. A recent study found that facility births decreased by over 50% during the lockdown in Nepal, and facility stillbirth and neonatal mortality rates increased significantly.[32] The NeoTree research team is currently collecting data on stillbirths at SMCH and KCH, but these data will still only represent stillbirths that occurred in a health facility. Given the COVID-19 pandemic is not over, it will be important to repeat our analysis over the coming months to further examine longer-term trends in neonatal care provision.

For peer review only - http://bmjopen.bmj.com/site/about/guidelines.xhtml

Conclusion

The indirect impacts of COVID-19 are context-specific, with more significant and evident effects on neonatal care provision seen at KCH (Malawi) than SMCH (Zimbabwe). While this study provides vital evidence to inform health providers and policy makers, national data are required to ascertain the true impacts of the pandemic on newborn health.

For peer review only

For peer review only - http://bmjopen.bmj.com/site/about/guidelines.xhtml

Figure Legends

Figure 1: Trend in daily admissions to the neonatal unit

- The seven-day moving average of daily admission numbers has been plotted.
- Smoothed line: local regression (LOESS) model fitted on the seven-day moving average of daily admission numbers; shaded region: 95% confidence interval.
- Solid vertical line: first confirmed case of COVID-19 in each country.
- Period between dashed vertical lines: industrial action by doctors in Zimbabwe.
- Counts based on all admission forms completed, irrespective of match status.
- *SMCH: Sally Mugabe Central Hospital; KCH: Kamuzu Central Hospital*

Figure 2: Interrupted time series for weekly admissions to the neonatal unit

- White background: pre-COVID-19 period; grey background: post-COVID-19 period.
- Solid line: predicted trend from regression model; dashed line: counterfactual scenario.
- SMCH model 1 (panel A) adjusted for doctors' strike period; SMCH model 2 (panel B) additionally adjusted for nurses' strike period; KCH model (panel C) unadjusted.
- Counts based on all admission forms completed, irrespective of match status.
- *SMCH: Sally Mugabe Central Hospital; KCH: Kamuzu Central Hospital*

Figure 3: Interrupted time series for outside referrals to the neonatal unit

- White background: pre-COVID-19 period; grey background: post-COVID-19 period.
- Solid line: predicted trend from regression model; dashed line: counterfactual scenario.
- SMCH model (panel A) adjusted for doctors' strike period, KCH model (panel B) unadjusted.
- Data from all admission forms completed, irrespective of match status.
- *SMCH: Sally Mugabe Central Hospital; KCH: Kamuzu Central Hospital*

Figure 4: Interrupted time series for overall mortality

- White background: pre-COVID-19 period; grey background: post-COVID-19 period.
- Solid line: predicted trend from regression model; dashed line: counterfactual scenario.
- SMCH model 1 (panel A) adjusted for doctors' strike period; SMCH model 2 (panel B) additionally adjusted for nurses' strike period; KCH model 1 (panel C) unadjusted level change model; KCH model 2 (panel D) unadjusted slope change model.
- Data from matched admission and outcome forms only.
- *SMCH: Sally Mugabe Central Hospital; KCH: Kamuzu Central Hospital*

Figure 5: Possible factors influencing the decrease in admissions to the neonatal unit

For peer review only

For peer review only - http://bmjopen.bmj.com/site/about/guidelines.xhtml

- Delays (red boxes) derived from the "Three Delays" model of pregnancy-related mortality.[33]
- *COVID-19: coronavirus disease 2019; PPE: personal protective equipment*

For peer review only

For peer review only - http://bmjopen.bmj.com/site/about/guidelines.xhtml

REFERENCES

1. World Health Organization. Statement on the second meeting of the International Health Regulations (2005) Emergency Committee regarding the outbreak of novel coronavirus (2019-nCoV) 2020 [cited 2020 04 Sept]. Available from: https://bit.ly/3jihlAU accessed 04 Sept 2020.

2. World Health Organization. WHO Coronavirus Disease (COVID-19) Dashboard 2020 [updated 2020 21 Oct; cited 2020 21 Oct]. Available from: https://covid19.who.int accessed 21 Oct 2020.

3. United Nations Inter-agency Group for Child Mortality Estimation. Levels & Trends in Child Mortality: Report 2019. New York: United Nations Children's Fund, 2019.

4. Makoni M. COVID-19 worsens Zimbabwe's health crisis. *Lancet* 2020;396(10249):457. doi: 10.1016/S0140-6736(20)31751-7

5. Truscott R. Covid-19: Health worker strikes, limited testing, and clinic closures hamper Zimbabwe's response. *BMJ* 2020;370:m3267. doi: 10.1136/bmj.m3267

6. Castagnoli R, Votto M, Licari A, et al. Severe Acute Respiratory Syndrome Coronavirus 2 (SARS-CoV-2) Infection in Children and Adolescents: A Systematic Review. *JAMA Pediatr* 2020 doi: 10.1001/jamapediatrics.2020.1467 [published Online First: 2020/04/23]

7. Götzinger F, Santiago-García B, Noguera-Julián A, et al. COVID-19 in children and adolescents in Europe: a multinational, multicentre cohort study. *Lancet Child Adolesc Health* 2020;4(9):653-61. doi: 10.1016/S2352-4642(20)30177-2

8. Ludvigsson JF. Systematic review of COVID-19 in children shows milder cases and a better prognosis than adults. *Acta Paediatr* 2020;109(6):1088-95. doi: 10.1111/apa.15270

9. Mantovani A, Rinaldi E, Zusi C, et al. Coronavirus disease 2019 (COVID-19) in children and/or adolescents: a meta-analysis. *Pediatr Res* 2020 doi: 10.1038/s41390-020-1015-2

10. Viner RM, Mytton OT, Bonell C, et al. Susceptibility to and transmission of COVID-19 amongst children and adolescents compared with adults: a systematic review and meta-analysis. *medRxiv* 2020:2020.05.20.20108126. doi: 10.1101/2020.05.20.20108126

11. Sochas L, Channon AA, Nam S. Counting indirect crisis-related deaths in the context of a low-resilience health system: the case of maternal and neonatal health during the Ebola epidemic in Sierra Leone. *Health Policy Plan* 2017;32(suppl_3):iii32-iii39. doi: 10.1093/heapol/czx108

12. Yerger P, Jalloh M, Coltart CEM, et al. Barriers to maternal health services during the Ebola outbreak in three West African countries: a literature review. *BMJ Glob Health* 2020;5(9) doi: 10.1136/bmjgh-2020-002974 [published Online First: 2020/09/09]

For peer review only

13. Ahmed S, Mvalo T, Akech S, et al. Protecting children in low-income and middle-income countries from COVID-19. *BMJ Glob Health* 2020;5(5) doi: 10.1136/bmjgh-2020-002844 [published Online First: 2020/05/29]

14. Roberton T, Carter ED, Chou VB, et al. Early estimates of the indirect effects of the COVID-19 pandemic on maternal and child mortality in low-income and middle-income countries: a modelling study. *Lancet Glob Health* 2020;8(7):e901-e08. doi: 10.1016/s2214-109x(20)30229-1 [published Online First: 2020/05/15]

15. NeoTree. NeoTree [GitHub repository]. 2020 [cited 2020 03 Oct]. Available from: https://github.com/neotree/neotree accessed 03 Oct 2020.

16. Crehan C, Kesler E, Nambiar B, et al. The NeoTree application: developing an integrated mHealth solution to improve quality of newborn care and survival in a district hospital in Malawi. *BMJ Glob Health* 2019;4(1):e000860. doi: 10.1136/bmjgh-2018-000860 [published Online First: 2019/02/05]

17. Gannon H, Chimhuya S, Chimhini G, et al. An electronic application to improve management of infections in low-income neonatal units: pilot implementation of the NeoTree Beta App in a public sector hospital in Zimbabwe. *medRxiv* 2020:2020.09.25.20201467. doi: 10.1101/2020.09.25.20201467

18. Government of Zimbabwe. Public Health (COVID-19 Prevention, Containment and Treatment) (National Lockdown) Order, 2020 Zimbabwe: Veritas Zimbabwe; 2020 [updated 28 Mar 2020; cited 2021 22 Jun]. Available from: https://www.veritaszim.net/node/4046.

19. Mzumara GW, Chawani M, Sakala M, et al. The health policy response to COVID-19 in Malawi. *BMJ Global Health* 2021;6(5):e006035. doi: 10.1136/bmjgh-2021-006035

20. Kaponda C. No COVID-19 lockdown still threatens livelihoods and trade in Malawi [Blog]. London School of Economics; 2020 [updated 25 Sept 2020; cited 2021 22 Jun]. Available from: https://blogs.lse.ac.uk/africaatlse/2020/09/25/no-covid19-lockdown-threatens-livelihoods-trade-trust-malawi/ accessed 22 Jun 2021.

21. R: A language and environment for statistical computing [program]. 3.6.3 version. Vienna, Austria: R Foundation for Statistical Computing, 2020.

22. RStudio: Integrated Development Environment for R [program]. 1.2.5033 version. Boston, MA: RStudio, Inc., 2019.

23. Bernal JL, Cummins S, Gasparrini A. Interrupted time series regression for the evaluation of public health interventions: a tutorial. *Int J Epidemiol* 2017;46(1):348-55. doi: 10.1093/ije/dyw098

24. Wedderburn RWM. Quasi-likelihood functions, generalized linear models, and the Gauss—Newton method. *Biometrika* 1974;61(3):439-47. doi: 10.1093/biomet/61.3.439

For peer review only - http://bmjopen.bmj.com/site/about/guidelines.xhtml

25. BBC News. Zimbabwe doctors end strike after billionaire's offer 2020 [cited 2020 04 Aug]. Available from: https://www.bbc.co.uk/news/world-africa-51205619 accessed 04 Aug 2020.

26. Reuters. Zimbabwe nurses end three-month strike over pay 2020 [cited 2020 20 Oct]. Available from: https://www.reuters.com/article/us-health-corononavirus-zimbabwe-strike-idUSKBN26011R accessed 20 Oct 2020.

27. Graham WJ, Afolabi B, Benova L, et al. Protecting hard-won gains for mothers and newborns in low-income and middle-income countries in the face of COVID-19: call for a service safety net. *BMJ Glob Health* 2020;5(6) doi: 10.1136/bmjgh-2020-002754 [published Online First: 2020/06/06]

28. Khalil A, Kalafat E, Benlioglu C, et al. SARS-CoV-2 infection in pregnancy: A systematic review and meta-analysis of clinical features and pregnancy outcomes. *EClinicalMedicine* 2020;25 doi: 10.1016/j.eclinm.2020.100446

29. Robertson NJ, Groenendaal F. Hypoxic-ischaemic brain injury. In: Rennie JM, ed. Rennie and Roberton's Textbook of Neonatology. 5th ed. London: Churchill Livingstone 2012:1114-55.

30. Say L, Chou D, Gemmill A, et al. Global causes of maternal death: a WHO systematic analysis. *Lancet Glob Health* 2014;2(6):e323-e33. doi: 10.1016/S2214-109X(14)70227-X

31. Hawley S, Ali MS, Berencsi K, et al. Sample size and power considerations for ordinary least squares interrupted time series analysis: a simulation study. *Clinical epidemiology* 2019;11:197-205. doi: 10.2147/CLEP.S176723

32. Kc A, Gurung R, Kinney MV, et al. Effect of the COVID-19 pandemic response on intrapartum care, stillbirth, and neonatal mortality outcomes in Nepal: a prospective observational study. *Lancet Glob Health* 2020;8(10):e1273-e81. doi: 10.1016/S2214-109X(20)30345-4

33. Thaddeus S, Maine D. Too far to walk: maternal mortality in context. *Soc Sci Med* 1994;38(8):1091-110. doi: 10.1016/0277-9536(94)90226-7 [published Online First: 1994/04/01]

For peer review only

Declaration of interests

The authors have no conflicts of interest to declare.

Acknowledgements

We are very grateful to the families at SMCH and KCH, and the staff members at both hospitals for their enthusiasm and commitment to the NeoTree project, without which this work would not be possible.

Funders

We would like to thank the funders of this study. Dr S. R. Neal was awarded the International Child Health Group David Morley Elective Bursary for this elective project. Funders of the wider NeoTree project, past and present, include the Wellcome Trust Digital Innovation Award (215742/Z/19/Z: PI: Heys), RCPCH, Naughton-Cliffe Mathews, UCL Grand Challenges and Global Engagement Fund, and the Healthcare Infection Society (SRG 201802004). Dr F. Fitzgerald is supported by the Academy of Medical Sciences and the funders of the Starter Grants for Clinical Lecturers scheme. This study and Drs M. Heys and F. Fitzgerald are further supported by the National Institute for Health Research Great Ormond Street Hospital Biomedical Research Centre. The funders had no role in study design, data collection and analysis, or preparation of this report.

Data sharing statement

Data collected for the study cannot yet be made publicly available yet because primary analysis for the pilot implementation evaluation of the NeoTree, as well as secondary analysis are ongoing. A goal of our pilot implementation is the establishment of an open-source anonymised research database of data collected using the NeoTree in order to maximise the reach and utility for researchers aiming to improve outcomes for neonates in low income settings. This database is under development and subject to negotiation with relevant Ministries of Health.

For peer review only

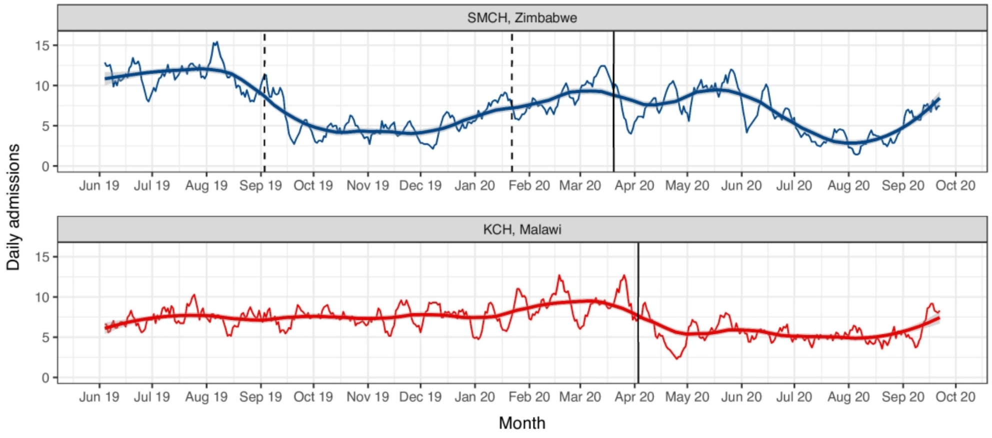

228x101mm (300 x 300 DPI)

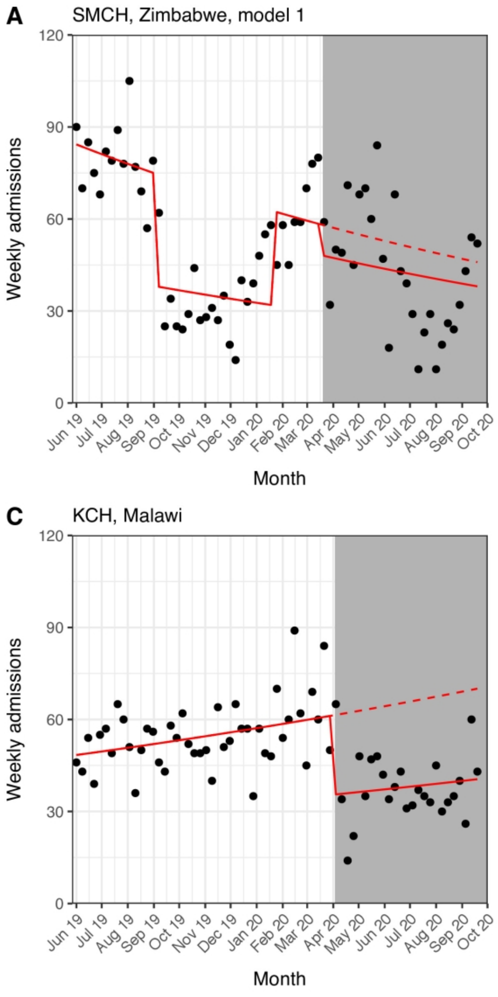

203x203mm (300 x 300 DPI)

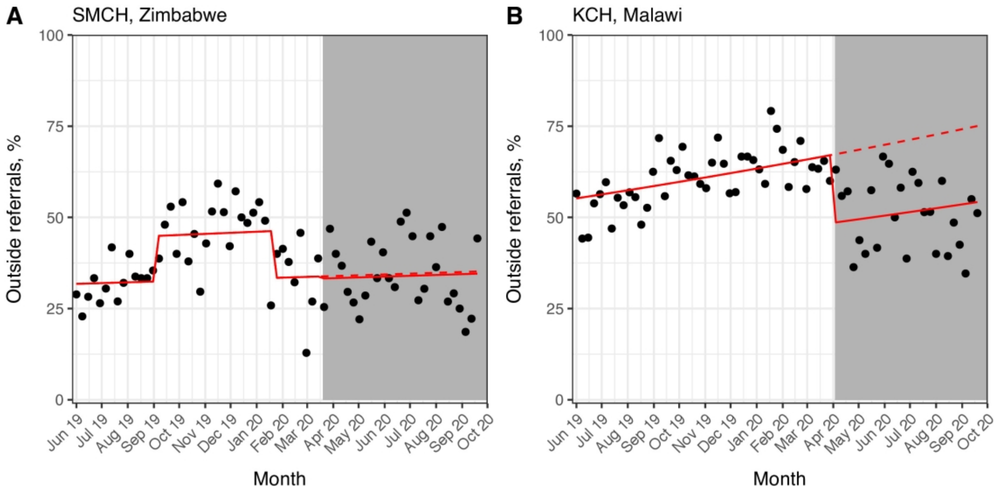

203x101mm (300 x 300 DPI)

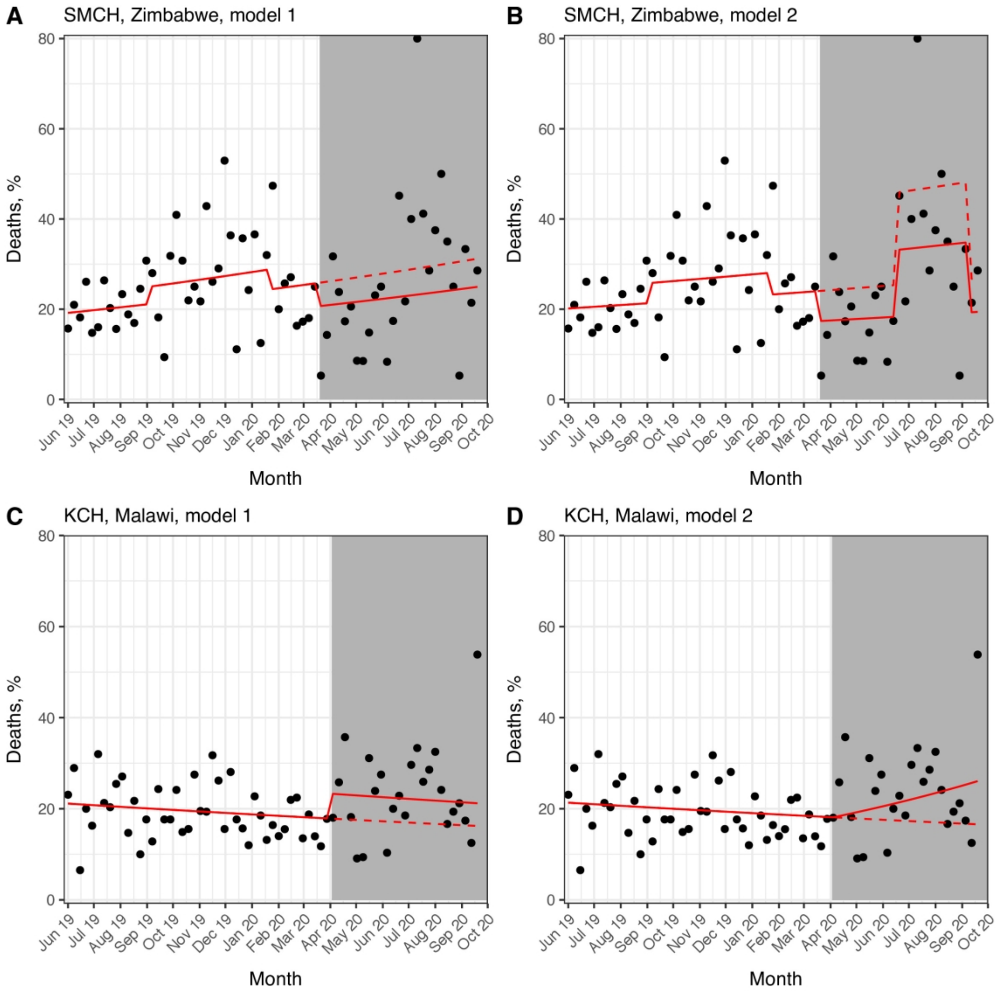

203x203mm (300 x 300 DPI)

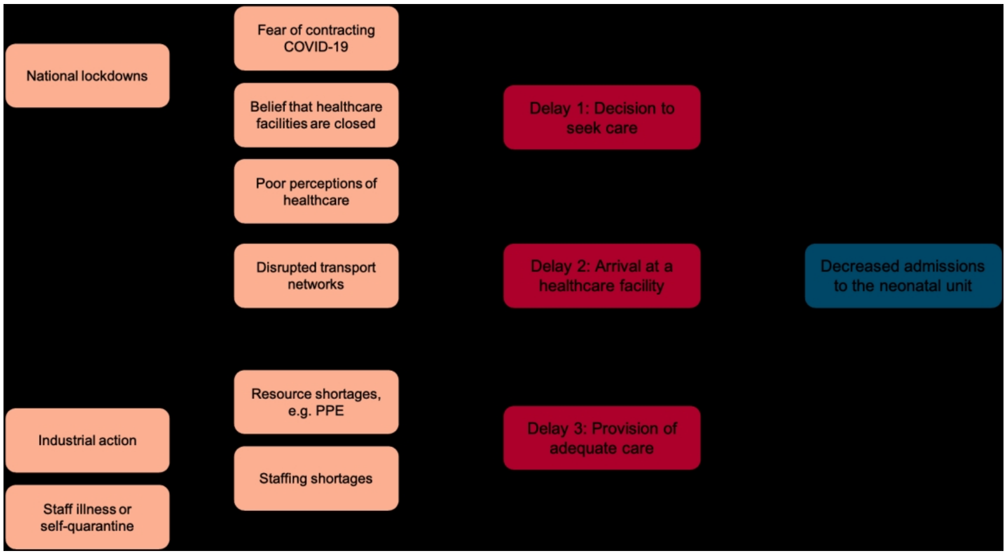

302x165mm (150 x 150 DPI)

SUPPLEMENTARY FIGURES

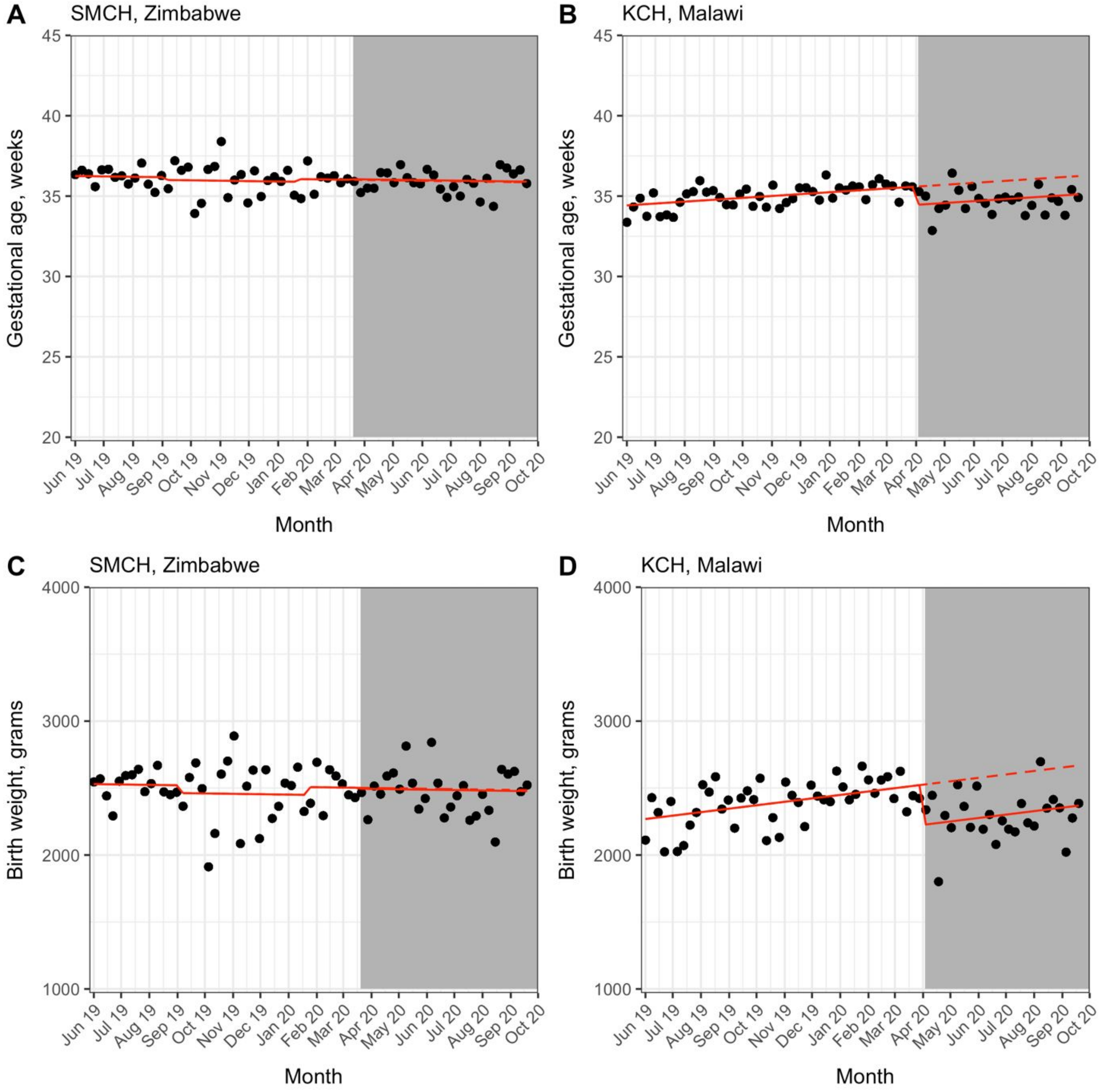

Supplementary Figure 1: Interrupted time series for gestational age and birth weight

- Data points represent weekly mean gestational age or birth weight to avoid overplotting.
- White background: pre-COVID-19 period; grey background: post-COVID-19 period.
- Solid line: predicted trend from regression model; dashed line: counterfactual scenario.
- SMCH models (panels A & C) adjusted for doctors' strike period, KCH models (panels B & D) unadjusted.
- Data from all admission forms completed, irrespective of match status.
- *SMCH: Sally Mugabe Central Hospital; KCH: Kamuzu Central Hospital*

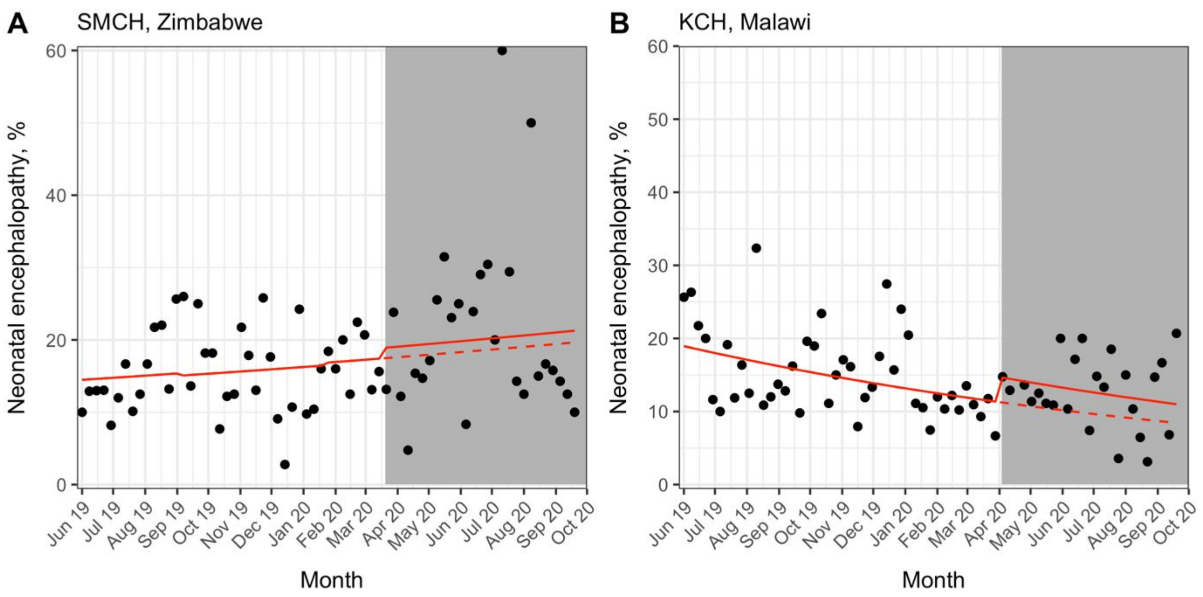

Supplementary Figure 2: Interrupted time series for prevalence of neonatal encephalopathy

- White background: pre-COVID-19 period; grey background: post-COVID-19 period.
- Solid line: predicted trend from regression model; dashed line: counterfactual scenario.
- SMCH model (panel A) adjusted for doctors' strike period, KCH model (panel B) unadjusted.
- Data from matched admission and outcome forms only.
- *SMCH: Sally Mugabe Central Hospital; KCH: Kamuzu Central Hospital*

For peer review only - http://bmjopen.bmj.com/site/about/guidelines.xhtml

APPENDIX 1: STROBE CHECKLIST

	Item No.	Recommendation	Page No.
**Title and abstract**	1	(*a*) Indicate the study's design with a commonly used term in the title or the abstract	1-2
		(*b*) Provide in the abstract an informative and balanced summary of what was done and what was found	2
**Introduction**			
Background/rationale	2	Explain the scientific background and rationale for the investigation being reported	4-5
Objectives	3	State specific objectives, including any prespecified hypotheses	5
**Methods**			
Study design	4	Present key elements of study design early in the paper	9
Setting	5	Describe the setting, locations, and relevant dates, including periods of recruitment, exposure, follow-up, and data collection	6
Participants	6	(*a*) *Cohort study*—Give the eligibility criteria, and the sources and methods of selection of participants. Describe methods of follow-up<br>*Case-control study*—Give the eligibility criteria, and the sources and methods of case ascertainment and control selection. Give the rationale for the choice of cases and controls<br>*Cross-sectional study*—Give the eligibility criteria, and the sources and methods of selection of participants	7
		(*b*) *Cohort study*—For matched studies, give matching criteria and number of exposed and unexposed<br>*Case-control study*—For matched studies, give matching criteria and the number of controls per case	
Variables	7	Clearly define all outcomes, exposures, predictors, potential confounders, and effect modifiers. Give diagnostic criteria, if applicable	7-8
Data sources/ measurement	8*	For each variable of interest, give sources of data and details of methods of assessment (measurement). Describe comparability of assessment methods if there is more than one group	7-8
Bias	9	Describe any efforts to address potential sources of bias	8-9
Study size	10	Explain how the study size was arrived at	7
Quantitative variables	11	Explain how quantitative variables were handled in the analyses. If applicable, describe which groupings were chosen and why	8-9
Statistical methods	12	(*a*) Describe all statistical methods, including those used to control for confounding	8-9
		(*b*) Describe any methods used to examine subgroups and interactions	8-9, Appendix 5, Appendix 6
		(*c*) Explain how missing data were addressed	8-9, Appendix 4
		(*d*) *Cohort study*—If applicable, explain how loss to follow-up was addressed	8-9

For peer review only - http://bmjopen.bmj.com/site/about/guidelines.xhtml

		Case-control study—If applicable, explain how matching of cases and controls was addressed<br>*Cross-sectional study*—If applicable, describe analytical methods taking account of sampling strategy	
		(*e*) Describe any sensitivity analyses	8-9, Appendix 5, Appendix 6
**Results**			
Participants	13*	(a) Report numbers of individuals at each stage of study—eg numbers potentially eligible, examined for eligibility, confirmed eligible, included in the study, completing follow-up, and analysed	11, Appendix 3
		(b) Give reasons for non-participation at each stage	9, 11, Appendix 3
		(c) Consider use of a flow diagram	Appendix 3
Descriptive data	14*	(a) Give characteristics of study participants (eg demographic, clinical, social) and information on exposures and potential confounders	11-14, Appendix 5
		(b) Indicate number of participants with missing data for each variable of interest	Appendix 4
		(c) *Cohort study*—Summarise follow-up time (eg, average and total amount)	11-14
Outcome data	15*	*Cohort study*—Report numbers of outcome events or summary measures over time	11-14
		Case-control study—Report numbers in each exposure category, or summary measures of exposure	
		Cross-sectional study—Report numbers of outcome events or summary measures	
Main results	16	(*a*) Give unadjusted estimates and, if applicable, confounder-adjusted estimates and their precision (eg, 95% confidence interval). Make clear which confounders were adjusted for and why they were included	11-14
		(*b*) Report category boundaries when continuous variables were categorized	11-14
		(*c*) If relevant, consider translating estimates of relative risk into absolute risk for a meaningful time period	11-14
Other analyses	17	Report other analyses done—eg analyses of subgroups and interactions, and sensitivity analyses	Appendix 5, Appendix 6
**Discussion**			
Key results	18	Summarise key results with reference to study objectives	15
Limitations	19	Discuss limitations of the study, taking into account sources of potential bias or imprecision. Discuss both direction and magnitude of any potential bias	17-18
Interpretation	20	Give a cautious overall interpretation of results considering objectives, limitations, multiplicity of analyses, results from similar studies, and other relevant evidence	15-17
Generalisability	21	Discuss the generalisability (external validity) of the study results	19
**Other information**			

For peer review only

For peer review only - http://bmjopen.bmj.com/site/about/guidelines.xhtml

Funding	22	Give the source of funding and the role of the funders for the present study and, if applicable, for the original study on which the present article is based	25

Adapted from: von Elm E, Altman DG, Egger M, Pocock SJ, Gøtzsche PC, et al. (2007) The Strengthening the Reporting of Observational Studies in Epidemiology (STROBE) Statement: Guidelines for Reporting Observational Studies. PLOS Medicine 4(10): e296. https://doi.org/10.1371/journal.pmed.0040296

For peer review only

For peer review only - http://bmjopen.bmj.com/site/about/guidelines.xhtml

APPENDIX 2: ETHICAL APPROVAL

Ethical approval for this study was granted by the following ethics committees.

Committee	Reference
United Kingdom	
University College London Research Ethics Committee	17123/001
Malawi	
College of Medicine Research and Ethics Committee	P.01/20/2909
Zimbabwe	
Medical Research Council of Zimbabwe	MRCZ/A/2570
Joint Research Ethics Committee for the University of Zimbabwe, College of Health Sciences and Parirenyatwa Group of Hospitals	JREC/327/19
Biomedical Research and Training Institute Institutional Review Board	AP155/2020
Sally Mugabe (Harare) Central Hospital Ethics Committee	071119/64

For peer review only

APPENDIX 3: FLOW DIAGRAMS OF RECORD INCLUSION

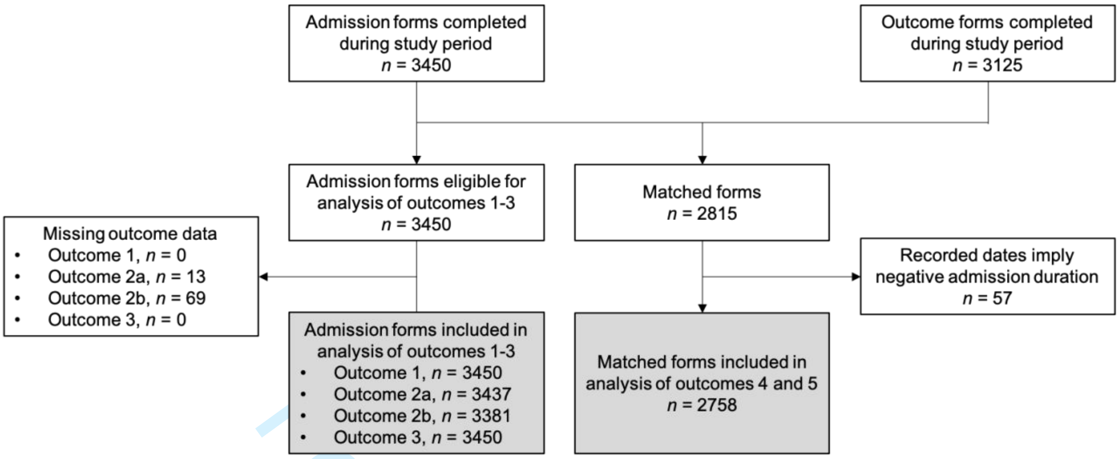

Flow diagram of record inclusion for analysis of data at Sally Mugabe Central Hospital, Zimbabwe

- Outcome 1: number of admissions
- Outcome 2a: gestational age
- Outcome 2b: birth weight
- Outcome 3: source of admission
- Outcome 4: prevalence of neonatal encephalopathy
- Outcome 5: overall mortality rate

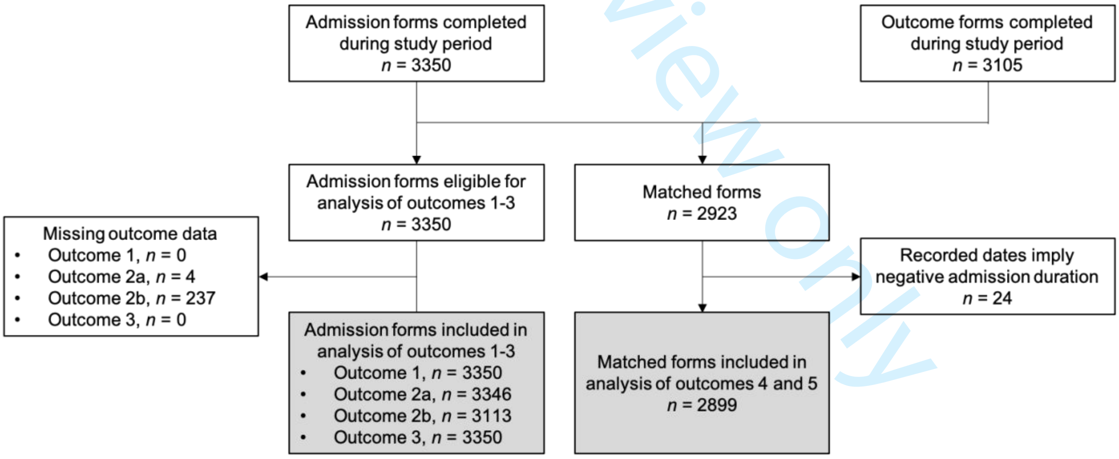

Flow diagram of record inclusion for analysis of data at Kamuzu Central Hospital, Malawi

- Outcome 1: number of admissions
- Outcome 2a: gestational age
- Outcome 2b: birth weight
- Outcome 3: source of admission
- Outcome 4: prevalence of neonatal encephalopathy
- Outcome 5: overall mortality rate

For peer review only

APPENDIX 4: MISSING DATA

The table below shows the number of participants with missing data for each outcome and the number of participants remaining for each analysis after pairwise deletion of missing values.

Characteristics	*n* missing (%)		*n* remaining*	
	SMCH	KCH	SMCH	KCH
Gestational age	13 (0·4)	4 (0·1)	3437 (99·6)	3346 (99·9)
Birth weight	69 (2·0)	237 (7·1)	3381 (98·0)	3113 (92·9)
Source of admission	0 (0·0)	0 (0·0)	3450 (100·0)	3350 (100·0)
Neonatal encephalopathy	0 (0·0)	0 (0·0)	2758 (100·0)†	2899 (100·0)†
Death	0 (0·0)	0 (0·0)	2758 (100·0)†	2899 (100·0)†

- * Remaining for analysis after pairwise deletion.
- † Only matched admission and outcome forms considered for analysis of neonatal encephalopathy and death.
- *SMCH: Sally Mugabe Central Hospital; KCH: Kamuzu Central Hospital, Malawi*

For peer review only

For peer review only - http://bmjopen.bmj.com/site/about/guidelines.xhtml

APPENDIX 5: FURTHER REGRESSION ANALYSIS RESULTS

Outcome 1: Admissions to the neonatal unit

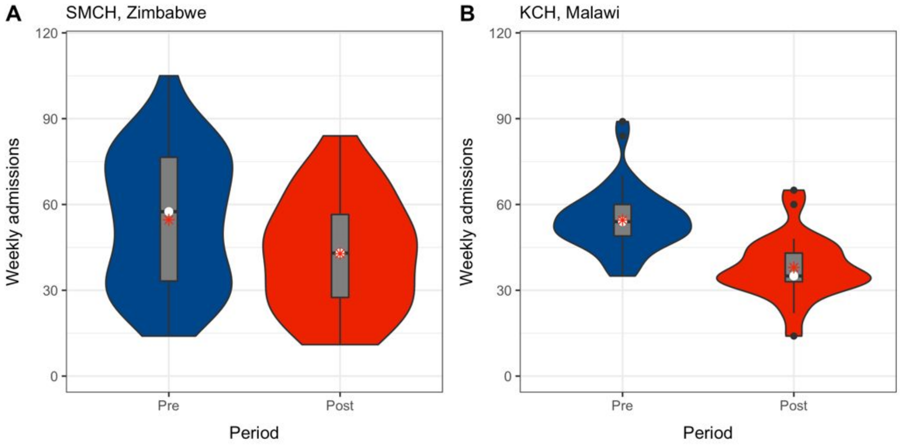

Distribution of weekly admissions by COVID-19 period

SMCH model 1: Level change model, adjusted for doctors' strike period

	Coef	SE	Exp	95% CI	*p*-value
Intercept	4·43	0·08	84·31	71·89 – 98·86	< 0·001
Post-COVID-19 period, yes	-0·19	0·17	0·83	0·60 – 1·14	0·25
Study time elapsed, weeks	-0·009	0·004	0·99	0·98 – 1·00	0·012
Doctors' strike period, yes	-0·68	0·11	0·51	0·41 – 0·63	< 0·001

SMCH model 2: Level change model, additionally adjusted for nurses' strike period

	Coef	SE	Exp	95% CI	*p*-value
Intercept	4·37	0·07	79·20	68·81 – 91·16	< 0·001
Post-COVID-19 period, yes	-0·11	0·14	0·90	0·69 – 1·17	0·90
Study time elapsed, weeks	-0·005	0·003	1·00	0·99 – 1·00	0·10
Doctors' strike period, yes	-0·70	0·09	0·50	0·41 – 0·60	< 0·001
Nurses' strike period, yes	-0·65	0·14	0·52	0·40 – 0·68	< 0·001

KCH model: Level change model, unadjusted

	Coef	SE	Exp	95% CI	*p*-value
Intercept	3·88	0·06	48·42	43·03 – 54·49	< 0·001
Post-COVID-19 period, yes	-0·55	0·10	0·58	0·48 – 0·70	< 0·001
Study time elapsed, weeks	0·005	0·002	1·01	1·00 – 1·01	0·019

For peer review only - http://bmjopen.bmj.com/site/about/guidelines.xhtml

Outcome 2: Gestational age at birth and birth weight

Gestational age at birth

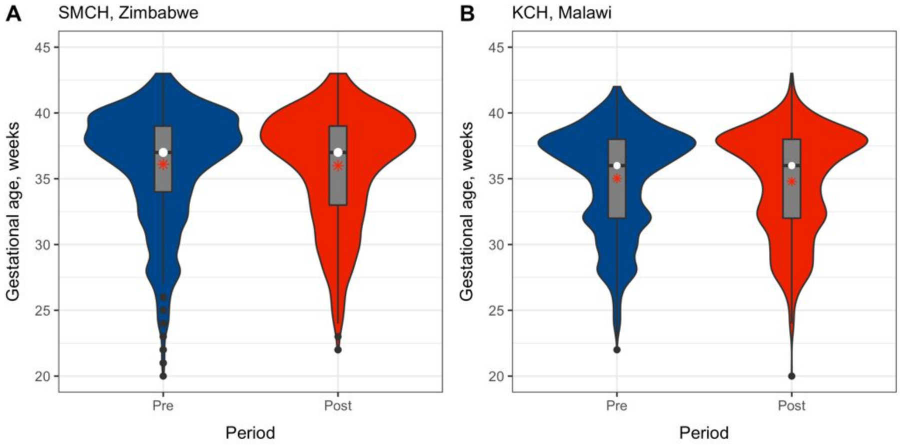

Distribution of gestational age at birth (weeks) by COVID-19 (pre/post-COVID19) period

SMCH model: Level change model, adjusted for doctors' strike period

	Coef	SE	95% CI	*p*-value
Intercept	36·25	0·15	35·96 – 36·54	< 0·001
Post-COVID-19 period, yes	0·04	0·29	-0·53 – 0·61	0·89
Study time elapsed, weeks	-0·006	0·006	-0·02 – 0·007	0·37
Doctors' strike period, yes	-0·17	0·20	-0·57 – 0·23	0·41

KCH model: Level change model, unadjusted

	Coef	SE	95% CI	*p*-value
Intercept	34·42	0·15	34·12 – 34·72	< 0·001
Post-COVID-19 period, yes	-1·14	0·25	-1·62 – -0·65	< 0·001
Study time elapsed, weeks	0·03	0·006	0·02 – 0·04	< 0·001

For peer review only - http://bmjopen.bmj.com/site/about/guidelines.xhtml

Birth weight

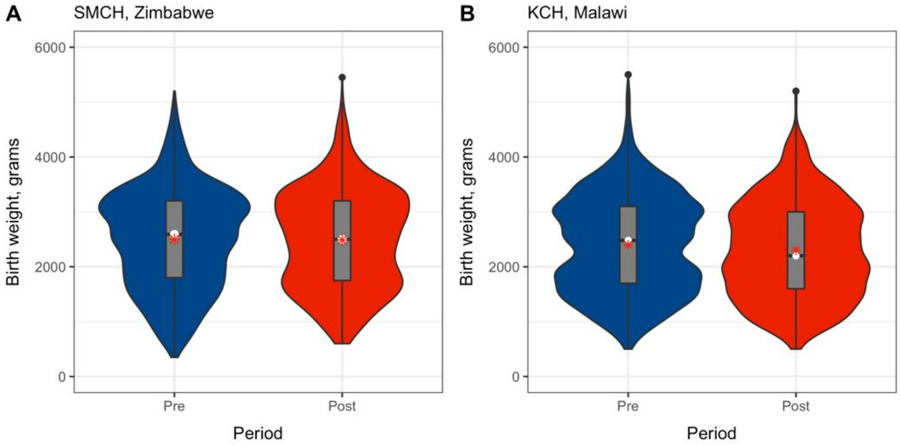

Distribution of birth weight (grams) by COVID-19 (pre/post-COVID19) period

SMCH model: Level change model, adjusted for doctors' strike period

	Coef	SE	95% CI	*p*-value
Intercept	2530·0	31·5	2468·0 – 2591·4	< 0·001
Post-COVID-19 period, yes	-7·2	61·1	-127·1 – 112·6	0·91
Study time elapsed, weeks	-0·7	1·3	-3·3 – 2·0	0·62
Doctors' strike period, yes	-58·1	42·9	-142·1 – 25·9	0·18

KCH model: Level change model, unadjusted

	Coef	SE	95% CI	*p*-value
Intercept	2269·0	36·0	2198·4 – 2339·6	< 0·001
Post-COVID-19 period, yes	-299·9	57·3	-412·3 – -187·5	< 0·001
Study time elapsed, weeks	5·9	1·4	3·2 – 8·6	< 0·001

For peer review only - http://bmjopen.bmj.com/site/about/guidelines.xhtml

Outcome 3: Source of admission referral

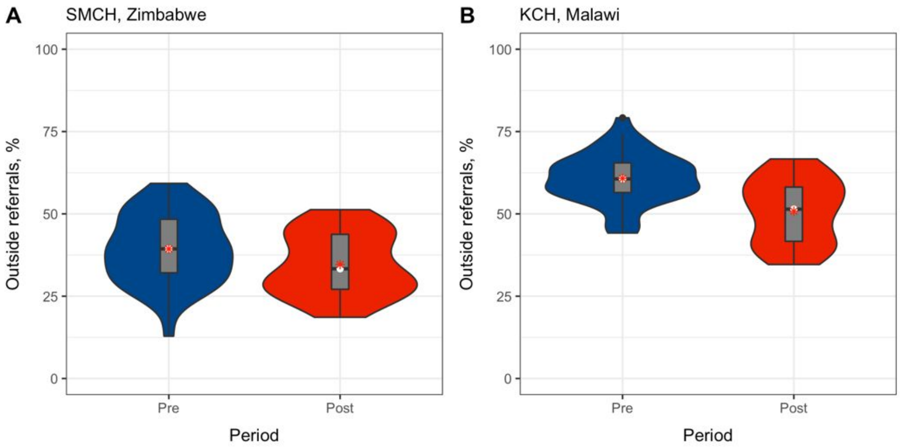

Distribution of outside referrals (%) by pre/post-COVID-19 period

SMCH model: Level change model, adjusted for doctors' strike period

	Coef	SE	Exp	95% CI	*p*-value
Intercept	-1·15	0·06	0·32	0·28 – 0·36	< 0·001
Post-COVID-19 period, yes	-0·02	0·11	0·98	0·79 – 1·23	0·88
Study time elapsed, weeks	0·001	0·003	1·00	1·00 – 1·01	0·55
Doctors' strike period, yes	0·33	0·07	1·39	1·20 – 1·60	< 0·001

KCH model: Level change model, unadjusted

	Coef	SE	Exp	95% CI	*p*-value
Intercept	-0·59	0·04	0·55	0·51 – 0·59	< 0·001
Post-COVID-19 period, yes	-0·33	0·06	0·72	0·65 – 0·81	< 0·001
Study time elapsed, weeks	0·005	0·001	1·01	1·00 – 1·01	0·001

For peer review only - http://bmjopen.bmj.com/site/about/guidelines.xhtml

Outcome 4: Prevalence of neonatal encephalopathy

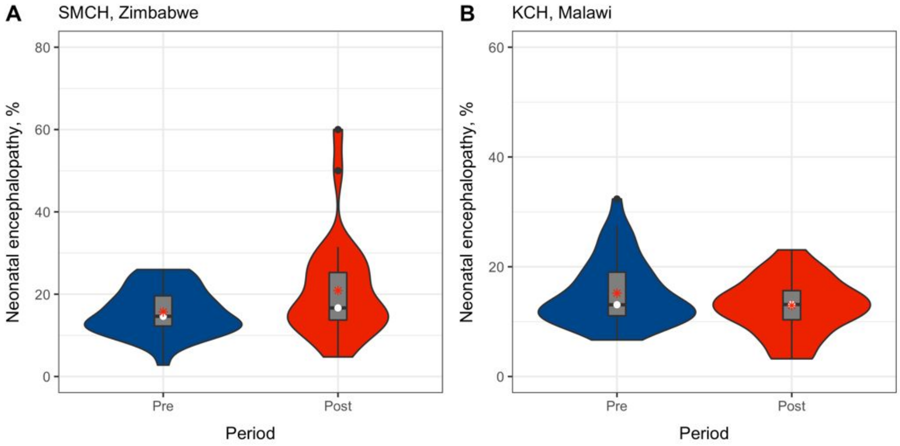

Distribution of neonatal encephalopathy (%) by pre/post-COVID-19 period

SMCH model: Level change model, adjusted for doctors' strike period

	Coef	SE	Exp	95% CI	*p*-value
Intercept	-1·93	0·10	0·15	0·12 – 0·18	< 0·001
Post-COVID-19 period, yes	0·08	0·18	1·08	0·76 – 1·55	0·67
Study time elapsed, weeks	0·004	0·004	1·00	1·00 – 1·01	0·27
Doctors' strike period, yes	-0·02	0·13	0·98	0·76 – 1·26	0·87

KCH model: Level change model, unadjusted

	Coef	SE	Exp	95% CI	*p*-value
Intercept	-1·66	0·09	0·19	0·16 – 0·23	< 0·001
Post-COVID-19 period, yes	0·27	0·16	1·30	0·95 – 1·80	0·11
Study time elapsed, weeks	-0·01	0·004	0·99	0·98 – 1·00	0·001

For peer review only - http://bmjopen.bmj.com/site/about/guidelines.xhtml

Outcome 5: Overall mortality

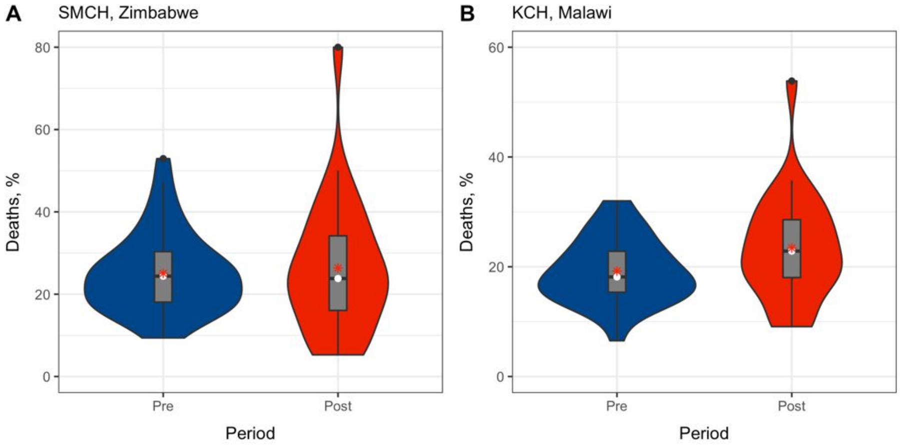

Distribution of overall mortality (%) by pre/post-COVID-19 period

SMCH model 1: Level change model, adjusted for doctors' strike period

	Coef	SE	Exp	95% CI	*p*-value
Intercept	-1·65	0·08	0·19	0·16 – 0·23	< 0·001
Post-COVID-19 period, yes	-0·22	0·16	0·80	0·56 – 1·15	0·23
Study time elapsed, weeks	0·007	0·003	1·01	1·00 – 1·02	0·09
Doctors' strike period, yes	0·17	0·10	1·19	0·94 – 1·50	0·16

SMCH model 2: Level change model, additionally adjusted for nurses' strike period

	Coef	SE	Exp	95% CI	*p*-value
Intercept	-1·60	0·09	0·20	0·17 – 0·24	< 0·001
Post-COVID-19 period, yes	-0·33	0·18	0·72	0·51 – 1·03	0·07
Study time elapsed, weeks	0·004	0·004	1·00	1·00 – 1·01	0·30
Doctors' strike period, yes	0·19	0·11	1·21	0·98 – 1·50	0·08
Nurses' strike period, yes	0·60	0·17	1·82	1·30 – 2·55	0·001

KCH model 1: Level change model, unadjusted

	Coef	SE	Exp	95% CI	*p*-value
Intercept	-1·56	0·09	0·21	0·18 – 0·25	< 0·001
Post-COVID-19 period, yes	0·27	0·14	1·31	0·98 – 1·73	0·07
Study time elapsed, weeks	-0·004	0·003	1·00	0·99 – 1·00	0·27

For peer review only - http://bmjopen.bmj.com/site/about/guidelines.xhtml

KCH model 2: Slope change model, unadjusted

	Coef	SE	Exp	95% CI	*p*-value
Intercept	-1·55	0·09	0·21	0·18 – 0·25	< 0·001
Study time elapsed, weeks	-0·004	0·003	1·00	0·99 – 1·00	0·25
Time since first COVID-19 case, weeks * post-COVID-19 period, yes	0·02	0·009	1·02	1·00 – 1·04	0·04

For peer review only

For peer review only - http://bmjopen.bmj.com/site/about/guidelines.xhtml

APPENDIX 6: ADDITIONAL ANALYSES

Mode of delivery of admitted neonates

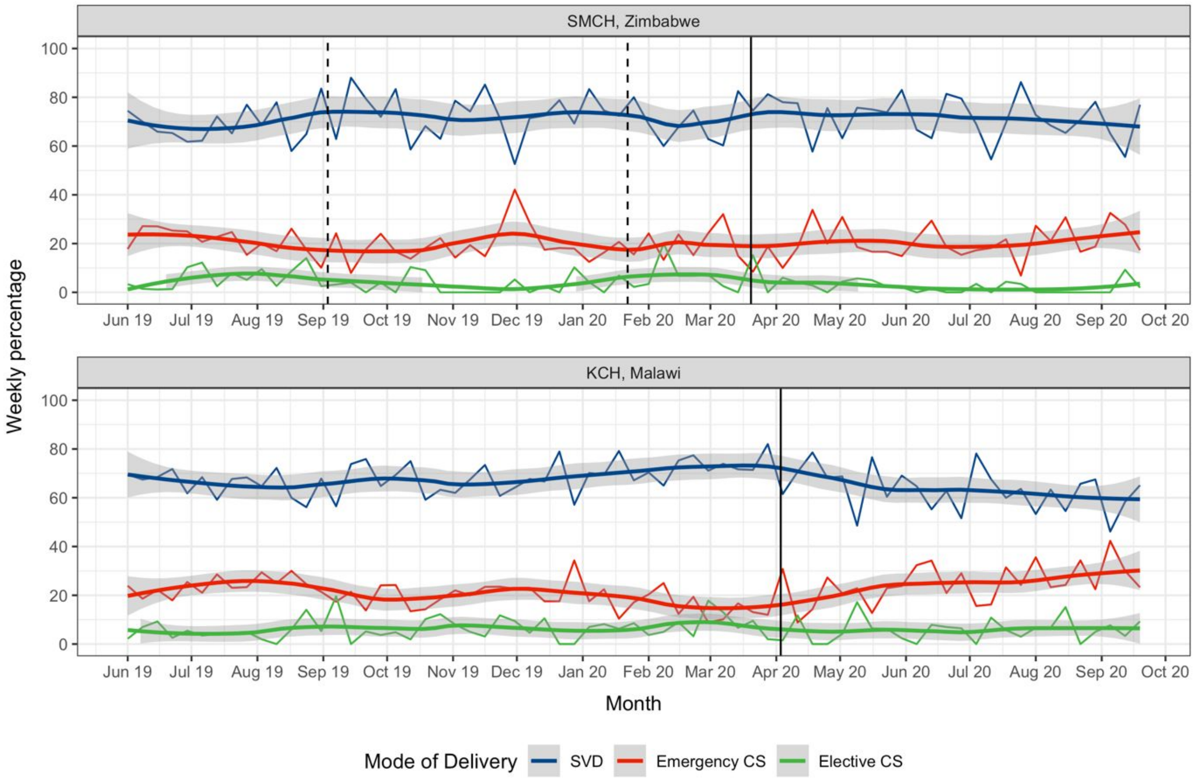

Trend in mode of delivery of admitted neonates per week

- Only SVD, emergency CS and elective CS displayed here to avoid overplotting.
- Smoothed line: local regression (LOESS) model; shaded region: 95% confidence interval.
- Solid vertical line: first confirmed case of COVID-19 in each country.
- Period between dashed vertical lines: industrial action by doctors in Zimbabwe.
- Counts based on all admission forms completed, irrespective of match status.
- *SMCH: Sally Mugabe Central Hospital; KCH: Kamuzu Central Hospital; SVD: spontaneous vaginal delivery; CS: caesarean section*

For peer review only - http://bmjopen.bmj.com/site/about/guidelines.xhtml

APPENDIX 1: STROBE CHECKLIST

	Item No.	Recommendation	Page No.
**Title and abstract**	1	(*a*) Indicate the study's design with a commonly used term in the title or the abstract	1-2
		(*b*) Provide in the abstract an informative and balanced summary of what was done and what was found	2
**Introduction**			
Background/rationale	2	Explain the scientific background and rationale for the investigation being reported	4-5
Objectives	3	State specific objectives, including any prespecified hypotheses	5
**Methods**			
Study design	4	Present key elements of study design early in the paper	9
Setting	5	Describe the setting, locations, and relevant dates, including periods of recruitment, exposure, follow-up, and data collection	6
Participants	6	(*a*) *Cohort study*—Give the eligibility criteria, and the sources and methods of selection of participants. Describe methods of follow-up<br>*Case-control study*—Give the eligibility criteria, and the sources and methods of case ascertainment and control selection. Give the rationale for the choice of cases and controls<br>*Cross-sectional study*—Give the eligibility criteria, and the sources and methods of selection of participants	7
		(*b*) *Cohort study*—For matched studies, give matching criteria and number of exposed and unexposed<br>*Case-control study*—For matched studies, give matching criteria and the number of controls per case	
Variables	7	Clearly define all outcomes, exposures, predictors, potential confounders, and effect modifiers. Give diagnostic criteria, if applicable	7-8
Data sources/ measurement	8*	For each variable of interest, give sources of data and details of methods of assessment (measurement). Describe comparability of assessment methods if there is more than one group	7-8
Bias	9	Describe any efforts to address potential sources of bias	8-9
Study size	10	Explain how the study size was arrived at	7
Quantitative variables	11	Explain how quantitative variables were handled in the analyses. If applicable, describe which groupings were chosen and why	8-9
Statistical methods	12	(*a*) Describe all statistical methods, including those used to control for confounding	8-9
		(*b*) Describe any methods used to examine subgroups and interactions	8-9, Appendix 5, Appendix 6
		(*c*) Explain how missing data were addressed	8-9, Appendix 4
		(*d*) *Cohort study*—If applicable, explain how loss to follow-up was addressed	8-9

For peer review only

		Case-control study—If applicable, explain how matching of cases and controls was addressed<br>*Cross-sectional study*—If applicable, describe analytical methods taking account of sampling strategy	
		(*e*) Describe any sensitivity analyses	8-9, Appendix 5, Appendix 6
**Results**			
Participants	13*	(a) Report numbers of individuals at each stage of study—eg numbers potentially eligible, examined for eligibility, confirmed eligible, included in the study, completing follow-up, and analysed	11, Appendix 3
		(b) Give reasons for non-participation at each stage	9, 11, Appendix 3
		(c) Consider use of a flow diagram	Appendix 3
Descriptive data	14*	(a) Give characteristics of study participants (eg demographic, clinical, social) and information on exposures and potential confounders	11-14, Appendix 5
		(b) Indicate number of participants with missing data for each variable of interest	Appendix 4
		(c) *Cohort study*—Summarise follow-up time (eg, average and total amount)	11-14
Outcome data	15*	*Cohort study*—Report numbers of outcome events or summary measures over time	11-14
		Case-control study—Report numbers in each exposure category, or summary measures of exposure	
		Cross-sectional study—Report numbers of outcome events or summary measures	
Main results	16	(*a*) Give unadjusted estimates and, if applicable, confounder-adjusted estimates and their precision (eg, 95% confidence interval). Make clear which confounders were adjusted for and why they were included	11-14
		(*b*) Report category boundaries when continuous variables were categorized	11-14
		(*c*) If relevant, consider translating estimates of relative risk into absolute risk for a meaningful time period	11-14
Other analyses	17	Report other analyses done—eg analyses of subgroups and interactions, and sensitivity analyses	Appendix 5, Appendix 6
**Discussion**			
Key results	18	Summarise key results with reference to study objectives	15
Limitations	19	Discuss limitations of the study, taking into account sources of potential bias or imprecision. Discuss both direction and magnitude of any potential bias	17-18
Interpretation	20	Give a cautious overall interpretation of results considering objectives, limitations, multiplicity of analyses, results from similar studies, and other relevant evidence	15-17
Generalisability	21	Discuss the generalisability (external validity) of the study results	19
**Other information**			

For peer review only

Funding	22	Give the source of funding and the role of the funders for the present study and, if applicable, for the original study on which the present article is based	25

Adapted from: von Elm E, Altman DG, Egger M, Pocock SJ, Gøtzsche PC, et al. (2007) The Strengthening the Reporting of Observational Studies in Epidemiology (STROBE) Statement: Guidelines for Reporting Observational Studies. PLOS Medicine 4(10): e296. https://doi.org/10.1371/journal.pmed.004029

For peer review only

For peer review only

BMJ Open

Indirect impacts of the COVID-19 pandemic at two tertiary neonatal units in Zimbabwe and Malawi: an interrupted time series analysis

Journal:	*BMJ Open*
Manuscript ID	bmjopen-2021-048955.R2
Article Type:	Original research
Date Submitted by the Author:	09-Feb-2022

Complete List of Authors:	Chimhuya, Simbarashe; University of Zimbabwe, Child and Adolescent Health Unit<br>Neal, Samuel; University College London, UCL Great Ormond Street Institute of Child Health<br>Chimhini, Gwen; University of Zimbabwe, Child and Adolescent Health Unit<br>Gannon, Hannah; University College London, UCL Great Ormond Street Institute of Child Health<br>Cortina Borja, Mario; University College London, UCL Great Ormond Street Institute of Child Health<br>Crehan, Caroline; University College London, UCL Great Ormond Street Institute of Child Health<br>Nkhoma, Deliwe; Parent and Child Health Initiative<br>Chiyaka, Tarisai; Biomedical Research and Training Institute<br>Wilson, Emma; University College London, UCL Great Ormond Street Institute of Child Health<br>Hull-Bailey, Tim; University College London, UCL Great Ormond Street Institute of Child Health<br>Fitzgerald, Felicity; University College London, UCL Great Ormond Street Institute of Child Health<br>Chiume, Msandeni; Kamuzu Central Hospital, Department of Paediatrics<br>Heys, Michelle; University College London Institute of Child Health, UCL Great Ormond Street Institute of Child Health

**Primary Subject Heading**:	Global health
Secondary Subject Heading:	Paediatrics, Public health, Infectious diseases, Epidemiology
Keywords:	COVID-19, PAEDIATRICS, Neonatal intensive & critical care < INTENSIVE & CRITICAL CARE, Quality in health care < HEALTH SERVICES ADMINISTRATION & MANAGEMENT, International health services < HEALTH SERVICES ADMINISTRATION & MANAGEMENT

For peer review only - http://bmjopen.bmj.com/site/about/guidelines.xhtml

I, the Submitting Author has the right to grant and does grant on behalf of all authors of the Work (as defined in the below author licence), an exclusive licence and/or a non-exclusive licence for contributions from authors who are: i) UK Crown employees; ii) where BMJ has agreed a CC-BY licence shall apply, and/or iii) in accordance with the terms applicable for US Federal Government officers or employees acting as part of their official duties; on a worldwide, perpetual, irrevocable, royalty-free basis to BMJ Publishing Group Ltd ("BMJ") its licensees and where the relevant Journal is co-owned by BMJ to the co-owners of the Journal, to publish the Work in this journal and any other BMJ products and to exploit all rights, as set out in our licence.

The Submitting Author accepts and understands that any supply made under these terms is made by BMJ to the Submitting Author unless you are acting as an employee on behalf of your employer or a postgraduate student of an affiliated institution which is paying any applicable article publishing charge ("APC") for Open Access articles. Where the Submitting Author wishes to make the Work available on an Open Access basis (and intends to pay the relevant APC), the terms of reuse of such Open Access shall be governed by a Creative Commons licence – details of these licences and which Creative Commons licence will apply to this Work are set out in our licence referred to above.

Other than as permitted in any relevant BMJ Author's Self Archiving Policies, I confirm this Work has not been accepted for publication elsewhere, is not being considered for publication elsewhere and does not duplicate material already published. I confirm all authors consent to publication of this Work and authorise the granting of this licence.

For peer review only

Indirect impacts of the COVID-19 pandemic at two tertiary neonatal units in Zimbabwe and Malawi: an interrupted time series analysis

Simbarashe Chimhuya, MMED*[1], Samuel R. Neal, MRes*[2], Gwendoline Chimhini, MMED[1], Hannah Gannon, MBChB[2], Mario Cortina-Borja, PhD[2], Caroline Crehan, MSc[2], Deliwe Nkhoma, MSc[3], Tarisai Chiyaka, MSc[4], Emma Wilson, PhD[2], Tim Hull-Bailey, MPhil[2], Felicity Fitzgerald, PhD[5], Msandeni Chiume, MBBS ‡[6], and Michelle Heys, MD(Res)‡ †[2]

1. Child and Adolescent Health Unit, Faculty of Medicine and Health Sciences, University of Zimbabwe, Harare, Zimbabwe
2. Population, Policy and Practice Research and Teaching Department, UCL Great Ormond Street Institute of Child Health, University College London, UK
3. Parent and Child Health Initiative Trust, Lilongwe, Malawi
4. Biomedical Research and Training Institute, Harare, Zimbabwe
5. Infection, Immunity and Inflammation, UCL Great Ormond Street Institute of Child Health, University College London, UK
6. Department of Paediatrics, Kamuzu Central Hospital, Lilongwe, Malawi

*Contributed equally as first author
†Corresponding author
‡ Contributed equally as last author

Correspondence to:
Dr Michelle Heys
Population, Policy and Practice Department,
UCL Great Ormond Street Institute of Child Health,
30 Guilford Street,
London, WC1N 1EH
Email: m.heys@ucl.ac.uk
Telephone: +44 (0)20 7905 2212

For peer review only

Word count: 3581

Keywords: COVID-19, neonatal, NICU, low-resource settings, global health

Contributors' statement

Concept and study design by SC, SRN, GC, FF, MCB, CC, MC and MH with input from other authors. Data collected by HG, DN, TC, CC and THB. Analysis performed by SRN and MCB with contributions from FF, SC, EW & MH. Manuscript drafted by SC and SRN with input from GC, FF, MCB, MC & MH. All authors proof-read and approved final draft. Underlying data accessed and verified by SRN, MCB, HG, FF & MH.

For peer review only - http://bmjopen.bmj.com/site/about/guidelines.xhtml

ABSTRACT

Objectives: To examine indirect impacts of the COVID-19 pandemic on neonatal care in low-income and middle-income countries.

Design: Interrupted time series analysis.

Setting: Two tertiary neonatal units in Harare, Zimbabwe and Lilongwe, Malawi.

Participants: We included a total of 6,800 neonates who were admitted to either neonatal unit from 1 June 2019 to 25 September 2020 (Zimbabwe: 3,450; Malawi: 3,350). We applied no specific exclusion criteria.

Interventions: The first cases of COVID-19 in each country (Zimbabwe: 20 March 2020; Malawi: 3 April 2020).

Primary outcome measures: Changes in the number of admissions, gestational age and birth weight, source of admission referrals, prevalence of neonatal encephalopathy, and overall mortality before and after the first cases of COVID-19.

Results: Admission numbers in Zimbabwe did not initially change after the first case of COVID-19 but fell by 48% during a nurses' strike (relative risk (RR) 0·52, 95% CI 0·41-0·66, *p* < 0·001). In Malawi, admissions dropped by 42% soon after the first case of COVID-19 (RR 0·58, 95% CI 0·48-0·70, *p* < 0·001). In Malawi, gestational age and birth weight decreased slightly by around one week (beta -1·14, 95% CI -1·62-(-)0·65, *p* < 0·001) and 300 grams (beta -299·9, 95% CI -412·3-(-)187·5, *p* < 0·001), and outside referrals dropped by 28% (RR 0·72, 95% CI 0·61-0·85, *p* < 0·001). No changes in these outcomes were found in Zimbabwe and no significant changes in the prevalence of neonatal encephalopathy or mortality were found at either site (*p* > 0·05).

Conclusions: The indirect impacts of COVID-19 are context-specific. While our study provides vital evidence to inform health providers and policy makers, national data are required to ascertain the true impacts of the pandemic on newborn health.

For peer review only - http://bmjopen.bmj.com/site/about/guidelines.xhtml

Strengths and limitations of this study

- We address the need for increased research into the indirect impacts of the COVID-19 pandemic on neonatal care in low-income and middle-income countries.
- We collected data digitally and in real time using the Neotree application, which enabled a large sample size of 6800 neonates with minimal missing data.
- It is possible that unobserved events occurred close to the first case of COVID-19 in either country, which could have influenced our results.
- We only collected data on neonates admitted to the neonatal unit and did not capture stillbirths or neonatal deaths that occurred in the community.

For peer review only

INTRODUCTION

The World Health Organization declared coronavirus disease (COVID-19) a Public Health Emergency of International Concern on 30 January 2020.[1] Almost two years later, confirmed cases have exceeded 281 million globally with over 5·4 million deaths to the end of 2021.[2] Zimbabwe recorded its first case on 20 March 2020 and, to date, has reported over 200,000 cases with nearly 5,000 deaths.[2] Malawi confirmed its first three cases on 3 April 2020 and has reported more than 72,000 cases and over 2,000 deaths in this same period.[2]

Before the COVID-19 pandemic, considerable improvements were made in global child health: the global neonatal mortality rate fell from 31 to 18 deaths per 1,000 live births between 2000 and 2018.[3] Yet there were disparities in the rates of decline with the sub-Saharan Africa region facing highest neonatal mortality rates.[3] Now, there is a danger that health outcomes in low-income and middle-income countries (LMICs) will fall further behind high-income countries. While countries worldwide face challenges related to the COVID-19 pandemic, LMICs are particularly struggling with financial constraints, limited testing capacity, lack of personal protective equipment, staff shortages,[4 5] and limited access to vaccines.[6] As children are at low risk of infection or severe disease from COVID-19,[7-11] any impacts on their health outcomes will likely be attributable to the indirect effects of the pandemic on health systems, as in previous disease outbreaks.[12 13] These include increased rates of parental unemployment, food and housing insecurity, and reduced access to routine care, including antenatal and perinatal care, with potentially damaging downstream impacts on neonatal outcomes.[14 15]

For peer review only - http://bmjopen.bmj.com/site/about/guidelines.xhtml

We hypothesised that the COVID-19 pandemic would negatively impact care seeking behaviours, neonatal care provision and, ultimately, neonatal outcomes in LMICs. To test this hypothesis, we aimed to examine trends in markers of neonatal care before and during the initial months of the COVID-19 pandemic at Sally Mugabe Central Hospital (SMCH), Zimbabwe, and Kamuzu Central Hospital (KCH), Malawi. Specifically, we compared the:

1. number of admissions to the neonatal unit (NNU),
2. gestational age and birth weight of admitted neonates,
3. source of admission referrals,
4. prevalence of neonatal encephalopathy (NE), and
5. overall mortality rate

before and after the first reported cases of COVID-19.

For peer review only

For peer review only - http://bmjopen.bmj.com/site/about/guidelines.xhtml

METHODS

This study is reported in accordance with the Strengthening the Reporting of Observational Studies in Epidemiology (STROBE) statement (Appendix 1).

Setting

Health facilities

SMCH is a public referral hospital in Harare, Zimbabwe. It has the largest of three tertiary NNUs nationwide with 100 cots. KCH, Lilongwe, is one of four regional referral hospitals in Malawi and the NNU has 75 cots. Neonatal care at SMCH is predominantly doctor led while neonatal care at KCH is mostly nurse led. Both units accept local and national referrals for specialist surgical care.

Government response to the pandemic

In response to the COVID-19 pandemic, Zimbabwe and Malawi both implemented response measures in an attempt to control the outbreak. In Zimbabwe, the Government closed borders to non-essential travel within days of the first in-country confirmed case of COVID-19 and imposed a full national lockdown that lasted from 30 March to 11 June 2020, which was followed by phased relaxations of the restrictions.[16] In Malawi, public events were banned and public gatherings restricted to fewer than 100 people on 20 March 2020, with all educational institutions closed several days later.[17] Borders were closed to non-essential travel on 1 April 2020 and a full national lockdown was announced to last for 21 days from 18 April 2020; however, a High Court injunction prevented this. Further restrictions were announced on 9 August 2020, mandating the wearing of face masks in public, closing places of worship,

For peer review only - http://bmjopen.bmj.com/site/about/guidelines.xhtml

restaurants, and bars, and restricting public gatherings to less than 10 people initially, although these were revised within days to reallow gatherings up to 100 people.[18]

Industrial action by health workers in Zimbabwe

Two periods of national industrial action occurred in Zimbabwe during our study. Doctors went on strike from 3 September 2019 to 22 January 2020 (pre-COVID-19 period) citing insufficient pay and poor working conditions, which put significant pressure on the public health system.[19] Additionally, there was a period of strikes by nurses from 17 June to 9 September 2020 (post-COVID-19 period) over pay and availability of personal protective equipment during the pandemic.[20]

Participants

All neonates admitted to each NNU over a 16-month period from 1 June 2019 to 25 September 2020 (69 complete weeks) were eligible for inclusion. We applied no specific exclusion criteria.

Data collection

Data were collected prospectively using the Neotree application (app), an Android tablet-based quality improvement platform that aims to reduce neonatal mortality in low-resource settings.[21] Developed in collaboration with local stakeholders, it is embedded in routine practice at two NNUs in Zimbabwe and Malawi, providing real-time clinical decision support, neonatal care education, and digital data capture.[22 23]

Health workers complete a digital form when a neonate is admitted to the unit (admission form) and when they are discharged or die (outcome form). The app guides

For peer review only - http://bmjopen.bmj.com/site/about/guidelines.xhtml

assessment of the neonate and collects data on patient demographics, examination findings, diagnoses, and interventions. Pseudonymised forms are uploaded monthly to University College London servers (Zimbabwe data) and Amazon Web Services (Malawi data). Admission and outcome forms are linked by a unique identifier generated by the app at admission.

Outcomes

We evaluated five outcomes:

1. Number of admissions: determined from the admission date of each completed admission form.
2. Gestational age at birth (weeks) and birth weight (grams): as entered into the admission form from obstetric records.
3. Source of admission: defined as 'within' (labour ward, postnatal ward, antenatal ward, obstetric theatre, or fee-paying ward [KCH only]) or 'outside' (referral from another health facility or postnatal self-referral from home).
4. Diagnosis of NE: defined as "hypoxic ischaemic encephalopathy" or "birth asphyxia" recorded as a diagnosis, cause of death or contributory cause of death on the outcome form.
5. Mortality: defined as an outcome of "neonatal death" on the outcome form. All other neonates, including those discharged, transferred to another facility or who left on parental request, were considered alive.

Ethical approval

Research ethics approval was granted by the UCL Research Ethics Committee (17123/001) and ethics committees in Malawi (P.01/20/2909) and Zimbabwe

For peer review only

(MRCZ/A/2570) (Appendix 2). The need to obtain informed consent was waived as we collected only pseudonymised data routinely documented for clinical care.

Statistical analysis

Analyses were performed in R version 3.6.3,[24] running on RStudio version 1.2.5033.[25] First, admission forms were matched with their corresponding outcome form based on the unique identifier generated at admission. Lack of completed outcome forms (SMCH: *n* = 325 [9·4% of admission forms completed]; KCH: *n* = 245 [7·3%]) or errors in entry of the unique identifier at discharge (SMCH: *n* = 310 [9·9% of outcome forms completed]; KCH: *n* = 182 [5·9%]) meant we were unable to match some admission forms with outcome forms (SMCH: *n* = 635 [18·4% of admission forms completed]; KCH: *n* = 427 [12·7%]). For outcomes 1-3, we based analyses on data from all admission forms, regardless of match status. For outcomes 4 and 5, we based analyses on matched records only. Matched records implying a negative admission duration (i.e. outcome date prior to admission date) were excluded (SMCH: *n* = 57 [2·0% of matched records]; KCH: *n* = 24 [0·8%]). See Appendix 3 for a flow diagram of record inclusion. Missing data were excluded using pairwise deletion for each analysis as frequencies of missing values were minimal (Appendix 4).

This study used an interrupted time series design with weekly data windows. We considered the first confirmed case of COVID-19 in each country as the intervention (Zimbabwe: 20 March 2020; Malawi: 3 April 2020).[2] For all outcomes, we hypothesised a level change impact model without a lag, and this was tested using interrupted time series regression models.[26] Gestational age and birth weight were modelled with linear regression. Count data were modelled using generalised linear models with Poisson

For peer review only - http://bmjopen.bmj.com/site/about/guidelines.xhtml

or negative binomial responses and logarithmic link functions. We assessed for dispersion by dividing the residual deviance by the degrees of freedom for the Poisson model. Where this quotient was much greater than one (greater than approximately 1.10) we instead used a negative binomial model to account for overdispersion. Accordingly, source of admission referral, prevalence of NE and overall mortality at SMCH were modelled using Poisson models, while number of admissions and overall mortality at KCH were modelled using negative binomial models.

All models for SMCH were adjusted for the periods of doctors' strikes (3 September 2019 to 22 January 2020) and nurses' strikes (17 June to 9 September 2020). For count data, we adjusted for variation in the number of admissions over time by including the logarithm of the number of admissions in each weekly window as an offset term. Presence of autocorrelation was assessed using autocorrelation function (ACF) plots and by examining models' residuals. Seasonality was included in the interrupted time series models with cosine functions with variable amplitude and shift. We tested models fitting cosine functions on week of admission with 6-month and 12-month periods, and a model including these two harmonic terms. To achieve this, we transformed each cosine function into a sine term and cosine term, and included these terms in the regression models for each outcome (as described by Stolwijk et al.[27]). The final models presented were selected by minimising the Bayesian Information Criterion (BIC) and by comparing goodness-of-fit with the χ^2-test for nested models. Adjusting for seasonality did not improve the fit of any of the models tested and, thus, all presented models are unadjusted for seasonality. See Appendix 5 for model selection and estimates.

For peer review only - http://bmjopen.bmj.com/site/about/guidelines.xhtml

Patient and Public Involvement

Although patients and the public were not directly involved in this study, within the broader Neotree co-development project we are carrying out a series of workshops and focus group discussions with healthcare workers and parents of admitted babies to ensure local ownership and relevance of this digital quality involvement tool aimed at improving healthcare outcomes for vulnerable neonates.

Role of the funding source

The funders had no role in study design, data collection, data analysis, data interpretation, or preparation of this manuscript.

For peer review only

RESULTS

Outcome 1: Admissions to the neonatal unit

We included 3,450 neonates at SMCH and 3,350 neonates at KCH. Figure 1 shows the seven-day moving average of admissions to the NNU.

At SMCH, the mean (SD) number of weekly admissions was 54·6 (23·5) before the first case of COVID-19 (pre-COVID-19) and 42·8 (19·9) afterwards (post-COVID-19). The negative binomial regression model showed no evidence of a change in admissions after the first case of COVID-19 (relative risk [RR] 0·87; 95% confidence interval [CI] 0·65-1·17; *p* = 0·37) (Figure 2A). However, this model estimated that admissions fell by 48% during the nurses' strike period (RR 0·52, 95%CI 0·41-0·66; *p* < 0·001) and by 51% during the pre-COVID-19 doctors' strikes (RR 0·49, 95%CI 0·41-0·60; *p* < 0·001).

At KCH, the mean (SD) number of weekly admissions was 54·5 (10·8) in the pre-COVID-19 period and 38·0 (10·9) in the post-COVID-19 period. The negative binomial regression model yielded a 42% reduction in admissions after the first case of COVID-19 (RR 0·58; 95%CI 0·48-0·70; *p* < 0·001) (Figure 2B).

Outcome 2: Gestational age and birth weight

At SMCH, the mean (SD) gestational age at birth was 36·1 (4·4) weeks in the pre-COVID-19 period and 36·0 (4·2) weeks in the post-COVID-19 period. The mean (SD) birth weight was 2500 (908) grams in the pre-COVID-19 period and 2487 (896) grams in the post-COVID-19 period. Linear regression analysis indicated no significant change in gestational age at birth nor birth weight after the first case of COVID-19

For peer review only - http://bmjopen.bmj.com/site/about/guidelines.xhtml

(gestational age: beta 0·07; 95%CI -0·50-0·64; p = 0·81, birth weight: beta 3·4; 95%CI -117·0-123·8; p = 0·96) (Supplementary Figure 1A, Supplementary Figure 1C).

At KCH, the mean (SD) gestational age was 35·0 (3·9) weeks in the pre-COVID-19 period and 34·8 (3·9) weeks in the post-COVID-19 period. The mean (SD) birth weight was 2402 (883) grams in the pre-COVID-19 period and 2299 (870) grams in the post-COVID-19 period. Gestational age significantly decreased by one week in the post-COVID-19 period (beta -1·14; 95%CI -1·62-(-)0·65; p < 0·001) (Supplementary Figure 1B) and birth weight significantly decreased by 300 grams (beta -299·9; 95%CI -412·3-(-)187·5; p < 0·001) (Supplementary Figure 1D).

Outcome 3: Source of admission referral

At SMCH, the mean (SD) percentage of outside referrals to the NNU was 39 (11)% in the pre-COVID-19 period and 35 (9)% in the post-COVID-19 period. The Poisson regression model showed no evidence of a change in the percentage of outside referrals after the first case of COVID-19 (RR 0·97; 95%CI 0·77-1·22; p = 0·81) (Figure 3A). However, this model did imply a 39% relative increase in the percentage of outside referrals during the doctors' strikes in the pre-COVID-19 period (RR 1·39; 95%CI 1·20-1·61; p < 0·001).

At KCH, the mean (SD) percentage of outside referrals was 61 (8)% in the pre-COVID-19 period and 51 (10)% in the post-COVID-19 period. Poisson regression analysis resulted in a 28% relative reduction in outside referrals after the first case of COVID-19 (RR 0·72; 95%CI 0·61-0·85; p < 0·001) (Figure 3B).

For peer review only

Outcome 4: Prevalence of neonatal encephalopathy

At SMCH, the mean (SD) percentage of admitted neonates diagnosed with NE was 16 (6)% in the pre-COVID-19 period and 21 (12)% in the post-COVID-19 period suggesting a possible increase. Poisson regression analysis showed no statistically significant change in the percentage of neonates diagnosed with NE post-COVID-19 (RR 1·06; 95%CI 0·74-1·52; p = 0·74) (Supplementary Figure 2A).

At KCH, the mean (SD) percentage of admitted neonates diagnosed with NE was 15 (6)% in the pre-COVID-19 period and 13 (5)% in the post-COVID-19 period. The Poisson regression model implied a possible increase in diagnoses of NE after the first case of COVID-19, but this was not statistically significant (RR 1·31; 95%CI 0·91-1·88; p = 0·15) (Supplementary Figure 2B).

Outcome 5: Overall mortality

For SMCH, the mean (SD) percentage of deaths per week of admission was 25 (10)% in the pre-COVID-19 period and 26 (16)% in the post-COVID-19 period. The negative binomial regression model pointed towards a possible decrease in mortality after the first case of COVID-19, but this was not statistically significant (RR 0·72; 95%CI 0·52-1·00; p = 0·05) (Figure 4A). However, this model did show an 81% relative increase in mortality during the nurses' strike period (RR 1·81; 95%CI 1·31-2·49; p < 0·001).

For KCH, the mean (SD) percentage of deaths per week of admission was 19 (6)% in the pre-COVID-19 period and 23 (10)% in the post-COVID-19 period. The Poisson regression model implied a possible increase in mortality after the first case of COVID-

For peer review only - http://bmjopen.bmj.com/site/about/guidelines.xhtml

For peer review only

19, but this was not statistically significant (RR 1·31; 95%CI 0·97-1·76; p = 0·08) (Figure 4B).

For peer review only

For peer review only - http://bmjopen.bmj.com/site/about/guidelines.xhtml

DISCUSSION

Summary

We performed an interrupted time series analysis to examine changes in neonatal care provision at two tertiary NNUs in Zimbabwe and Malawi after the first cases of COVID-19 in each country. We found that admissions at SMCH did not change significantly after the first case of COVID-19 when considering this period as a whole, but there was a considerable decrease (around 50%) in the number admissions in June to August 2020, coinciding with a nurses' strike. We did not find significant changes in gestational age or birth weight, source of admission referrals, prevalence of NE or mortality at SMCH. Conversely, we found several changes in markers of neonatal care at KCH after the first case of COVID-19 in Malawi. The number of admissions fell by 42% and we noted a decrease in the gestational age and birth weight of admitted neonates (by around one week and 300 grams, respectively), and a 28% relative decrease in outside referrals after the first case of COVID-19. Although this study is descriptive, we can speculate about explanations for our results based on existing literature and discussions with local health workers.

Interpretation

The number of admissions at SMCH fell by around 50% between June to August 2020, but we noted no change outside this strike period, suggesting some resilience to the impact of the pandemic. However, nurses went on strike over pay and availability of personal protective equipment,[20] so the strike is itself an indirect consequence of COVID-19. A recently published audit of maternal health service provision at two tertiary hospitals in Harare, Zimbabwe (including SMCH) found a 25% reduction in hospital deliveries and an increased odds of stillbirth (OR 1·8; 95%CI 1·5-2·2) in March

For peer review only - http://bmjopen.bmj.com/site/about/guidelines.xhtml

to August 2020 compared to the same period in 2019,[28] which might partially explain the reduction in admissions to the NNU. A similar reduction in admissions was seen at KCH, but, unlike at SMCH, this 42% decrease was noted within a week of the first case of COVID-19. In Figure 5, we propose several interlinked factors that might explain reduced admissions to the NNU. Several of these factors, such as fear of using health services, disrupted transport networks and staff shortages have been directly reported by local sources in low-resource settings and were highlighted in a recent report by Graham et al.[29]

We found a slight decrease in gestational age and birth weight of neonates at KCH, but not SMCH. Studies have reported increased rates of preterm birth in pregnant women with COVID-19 compared to those without the disease, mostly from medically-induced preterm birth; although none of these studies were conducted in LMICs.[30] Preliminary analysis suggests rates of emergency caesarean section increased at SMCH and KCH, with a more marked increase at KCH (Appendix 6). This is one potential explanation for our findings. However, we noted that the number of outside referrals decreased by 28% at KCH, and neonates referred from outside KCH are more likely to be from lower-risk pregnancies that delivered in a health centre with higher gestational ages and birth weights. Further analysis should stratify by source of admission referral to clarify this finding, but the relative reduction in outside referrals is supported by the fact that referrals were rigorously triaged by the on-call paediatrician during the pandemic, and that referrals from some areas were diverted away from KCH to more appropriate centres for the level of care required.

For peer review only - http://bmjopen.bmj.com/site/about/guidelines.xhtml
For peer review only

We hypothesised that rates of NE would increase during the pandemic. NE is the clinical manifestation of disordered brain function and can have multiple aetiologies.[31] The term 'hypoxic-ischaemic encephalopathy' is reserved for cases where there is evidence of intrapartum asphyxia.[31] In LMICs, obstructed labour is a major cause of maternal mortality and can lead to intrapartum asphyxia with subsequent neonatal morbidity and mortality, including NE.[32] Therefore, the prevalence of NE might be expected to increase as a marker of delayed presentation to a health facility. It is reassuring that we did not find increased rates of NE at SMCH or KCH. However, these findings should be interpreted cautiously as some neonates with NE may not have presented to a health facility at all, for example, due to an increased number of home deliveries, as documented in other sub-Saharan countries.[33]

Finally, we did not observe a significant change in overall mortality at KCH nor SMCH, except during the nurses' strikes at SMCH. In fact, there was a suggestion that mortality decreased after the first case of COVID-19 in Zimbabwe when adjusted for the nurses' strike period, but this was not statistically significant. The reasons for this are unclear but could include factors such as increased stillbirth rates or improved care for the smaller number of neonates on the NNU. More complete analysis of facility-based and community-based neonatal mortality is greatly needed.

Limitations and future work

A limitation intrinsic to interrupted time series analysis is the possibility that another event occurred close to the first case of COVID-19 in either country causing spurious observations. Another potential threat to validity is changing data collection practices. For example, overstretched clinicians might not input data into the Neotree app for all

For peer review only - http://bmjopen.bmj.com/site/about/guidelines.xhtml

admitted neonates. However, this is unlikely as the Neotree app is embedded into routine practice at SMCH and KCH and discussions with local collaborators suggest use of the app has continued without issue. At present, there is limited guidance on power and sample size calculations for interrupted time series analyses.[34] Therefore, we did not perform specific power calculations and relied on the data available at the time of analysis. Also, our results suggest that our study has relatively low power to detect true changes in some outcomes, particularly NE, so these results should be interpreted cautiously in the absence of further data.

The Neotree app only collects data on neonates admitted to the NNU. Therefore, our analysis does not capture stillbirths or neonatal deaths that occur in the community. It is troubling to see a dramatic fall in admissions at both sites, raising the possibility that many unwell neonates did not attend a health facility and died at home. A recent study found that facility births decreased by over 50% during the lockdown in Nepal, and facility stillbirth and neonatal mortality rates increased significantly.[35] The Neotree research team is currently collecting data on stillbirths at SMCH and KCH, but these data will still only represent stillbirths that occurred in a health facility. Given the COVID-19 pandemic is not over, it will be important to repeat our analysis to further examine longer-term trends in neonatal care provision.

Conclusion

The indirect impacts of COVID-19 are context-specific, with more significant and evident effects on neonatal care provision seen at KCH (Malawi) than SMCH (Zimbabwe). While this study provides vital evidence to inform health providers and

For peer review only

policy makers, national data are required to ascertain the true impacts of the pandemic on newborn health.

For peer review only

For peer review only - http://bmjopen.bmj.com/site/about/guidelines.xhtml

Figure Legends

Figure 1: Trend in daily admissions to the neonatal unit

- The seven-day moving average of daily admission numbers has been plotted.
- Smoothed line: local regression (LOESS) model fitted on the seven-day moving average of daily admission numbers; shaded region: 95% confidence interval.
- Solid vertical line: first confirmed case of COVID-19 in each country.
- Shaded periods on SMCH, Zimbabwe panel: industrial action by doctors (3 September 2019 to 22 January 2020) and nurses (17 July 2020 to 9 September 2020).
- Counts based on all admission forms completed, irrespective of match status.
- *SMCH: Sally Mugabe Central Hospital; KCH: Kamuzu Central Hospital*

Figure 2: Interrupted time series for weekly admissions to the neonatal unit

- White background: pre-COVID-19 period; grey background: post-COVID-19 period.
- Solid line: predicted trend from negative binomial regression model; dashed line: counterfactual scenario.
- SMCH model (panel A) adjusted for doctors' and nurses' strike periods; KCH model (panel B) unadjusted.
- Counts based on all admission forms completed, irrespective of match status.
- *SMCH: Sally Mugabe Central Hospital; KCH: Kamuzu Central Hospital*

Figure 3: Interrupted time series for outside referrals to the neonatal unit

- White background: pre-COVID-19 period; grey background: post-COVID-19 period.
- Solid line: predicted trend from Poisson regression model; dashed line: counterfactual scenario.
- SMCH model (panel A) adjusted for doctors' and nurses' strike periods , KCH model (panel B) unadjusted.
- Data from all admission forms completed, irrespective of match status.
- *SMCH: Sally Mugabe Central Hospital; KCH: Kamuzu Central Hospital*

For peer review only

For peer review only - http://bmjopen.bmj.com/site/about/guidelines.xhtml

Figure 4: Interrupted time series for overall mortality

- White background: pre-COVID-19 period; grey background: post-COVID-19 period.
- Solid line: predicted trend from negative binomial regression model (SMCH, panel A) or Poisson regression model (KCH, panel B); dashed line: counterfactual scenario.
- SMCH model (panel A) adjusted for doctors' and nurses' strike periods; KCH model (panel B) unadjusted.
- Data from matched admission and outcome forms only.
- *SMCH: Sally Mugabe Central Hospital; KCH: Kamuzu Central Hospital*

Figure 5: Possible factors influencing the decrease in admissions to the neonatal unit

- Delays (red boxes) derived from the "Three Delays" model of pregnancy-related mortality.[36]
- *COVID-19: coronavirus disease 2019; PPE: personal protective equipment*

For peer review only

REFERENCES

1. World Health Organization. Statement on the second meeting of the International Health Regulations (2005) Emergency Committee regarding the outbreak of novel coronavirus (2019-nCoV) 2020 [cited 2020 04 Sept]. Available from: https://bit.ly/3jihlAU accessed 04 Sept 2020.

2. World Health Organization. WHO Coronavirus Disease (COVID-19) Dashboard 2021 [updated 2021 31 Dec; cited 2021 31 Dec]. Available from: https://covid19.who.int accessed 31 Dec 2021.

3. United Nations Inter-agency Group for Child Mortality Estimation. Levels & Trends in Child Mortality: Report 2019. New York: United Nations Children's Fund, 2019.

4. Makoni M. COVID-19 worsens Zimbabwe's health crisis. *Lancet* 2020;396(10249):457. doi: 10.1016/S0140-6736(20)31751-7

5. Truscott R. Covid-19: Health worker strikes, limited testing, and clinic closures hamper Zimbabwe's response. *BMJ* 2020;370:m3267. doi: 10.1136/bmj.m3267

6. World Health Organization. Less than 10% of African countries to hit key COVID-19 vaccination goal 2021 [cited 2021 31 Dec]. Available from: https://bit.ly/3EDj6mN accessed 31 Dec 2021.

7. Castagnoli R, Votto M, Licari A, et al. Severe Acute Respiratory Syndrome Coronavirus 2 (SARS-CoV-2) Infection in Children and Adolescents: A Systematic Review. *JAMA Pediatr* 2020 doi: 10.1001/jamapediatrics.2020.1467 [published Online First: 2020/04/23]

8. Götzinger F, Santiago-García B, Noguera-Julián A, et al. COVID-19 in children and adolescents in Europe: a multinational, multicentre cohort study. *Lancet Child Adolesc Health* 2020;4(9):653-61. doi: 10.1016/S2352-4642(20)30177-2

9. Ludvigsson JF. Systematic review of COVID-19 in children shows milder cases and a better prognosis than adults. *Acta Paediatr* 2020;109(6):1088-95. doi: 10.1111/apa.15270

10. Mantovani A, Rinaldi E, Zusi C, et al. Coronavirus disease 2019 (COVID-19) in children and/or adolescents: a meta-analysis. *Pediatr Res* 2020 doi: 10.1038/s41390-020-1015-2

11. Viner RM, Mytton OT, Bonell C, et al. Susceptibility to and transmission of COVID-19 amongst children and adolescents compared with adults: a systematic review and meta-analysis. *medRxiv* 2020:2020.05.20.20108126. doi: 10.1101/2020.05.20.20108126

12. Sochas L, Channon AA, Nam S. Counting indirect crisis-related deaths in the context of a low-resilience health system: the case of maternal and neonatal health during the Ebola epidemic in Sierra Leone. *Health Policy Plan* 2017;32(suppl_3):iii32-iii39. doi: 10.1093/heapol/czx108

For peer review only

For peer review only - http://bmjopen.bmj.com/site/about/guidelines.xhtml

13. Yerger P, Jalloh M, Coltart CEM, et al. Barriers to maternal health services during the Ebola outbreak in three West African countries: a literature review. *BMJ Glob Health* 2020;5(9) doi: 10.1136/bmjgh-2020-002974 [published Online First: 2020/09/09]

14. Ahmed S, Mvalo T, Akech S, et al. Protecting children in low-income and middle-income countries from COVID-19. *BMJ Glob Health* 2020;5(5) doi: 10.1136/bmjgh-2020-002844 [published Online First: 2020/05/29]

15. Roberton T, Carter ED, Chou VB, et al. Early estimates of the indirect effects of the COVID-19 pandemic on maternal and child mortality in low-income and middle-income countries: a modelling study. *Lancet Glob Health* 2020;8(7):e901-e08. doi: 10.1016/s2214-109x(20)30229-1 [published Online First: 2020/05/15]

16. Government of Zimbabwe. Public Health (COVID-19 Prevention, Containment and Treatment) (National Lockdown) Order, 2020 Zimbabwe: Veritas Zimbabwe; 2020 [updated 28 Mar 2020; cited 2021 22 Jun]. Available from: https://www.veritaszim.net/node/4046.

17. Mzumara GW, Chawani M, Sakala M, et al. The health policy response to COVID-19 in Malawi. *BMJ Global Health* 2021;6(5):e006035. doi: 10.1136/bmjgh-2021-006035

18. Kaponda C. No COVID-19 lockdown still threatens livelihoods and trade in Malawi [Blog]. London School of Economics; 2020 [updated 25 Sept 2020; cited 2021 22 Jun]. Available from: https://blogs.lse.ac.uk/africaatlse/2020/09/25/no-covid19-lockdown-threatens-livelihoods-trade-trust-malawi/.

19. BBC News. Zimbabwe doctors end strike after billionaire's offer 2020 [cited 2020 04 Aug]. Available from: https://www.bbc.co.uk/news/world-africa-51205619 accessed 04 Aug 2020.

20. Reuters. Zimbabwe nurses end three-month strike over pay 2020 [cited 2020 20 Oct]. Available from: https://www.reuters.com/article/us-health-corononavirus-zimbabwe-strike-idUSKBN26011R accessed 20 Oct 2020.

21. NeoTree. NeoTree [GitHub repository]. 2020 [cited 2020 03 Oct]. Available from: https://github.com/neotree/neotree accessed 03 Oct 2020.

22. Crehan C, Kesler E, Nambiar B, et al. The NeoTree application: developing an integrated mHealth solution to improve quality of newborn care and survival in a district hospital in Malawi. *BMJ Glob Health* 2019;4(1):e000860. doi: 10.1136/bmjgh-2018-000860 [published Online First: 2019/02/05]

23. Gannon H, Chimhuya S, Chimhini G, et al. Electronic application to improve management of infections in low-income neonatal units: pilot implementation of the NeoTree beta app in a public sector hospital in Zimbabwe. *BMJ Open Quality* 2021;10(1) doi: 10.1136/bmjoq-2020-001043

24. R: A language and environment for statistical computing [program]. 3.6.3 version. Vienna, Austria: R Foundation for Statistical Computing, 2020.

For peer review only - http://bmjopen.bmj.com/site/about/guidelines.xhtml

For peer review only

25. RStudio: Integrated Development Environment for R [program]. 1.2.5033 version. Boston, MA: RStudio, Inc., 2019.

26. Bernal JL, Cummins S, Gasparrini A. Interrupted time series regression for the evaluation of public health interventions: a tutorial. *Int J Epidemiol* 2017;46(1):348-55. doi: 10.1093/ije/dyw098

27. Stolwijk AM, Straatman H, Zielhuis GA. Studying seasonality by using sine and cosine functions in regression analysis. *J Epidemiol Community Health* 1999;53(4):235-8. doi: 10.1136/jech.53.4.235 [published Online First: 1999/07/09]

28. Bikwa Y, Murewanhema G, Kanyangarara M, et al. Impact of COVID-19 on maternal and perinatal outcomes in Harare, Zimbabwe: a comparative maternal audit. *Journal of Global Health Reports* 2021;5(e2021093) doi: doi:10.29392/001c.28995

29. Graham WJ, Afolabi B, Benova L, et al. Protecting hard-won gains for mothers and newborns in low-income and middle-income countries in the face of COVID-19: call for a service safety net. *BMJ Glob Health* 2020;5(6) doi: 10.1136/bmjgh-2020-002754 [published Online First: 2020/06/06]

30. Khalil A, Kalafat E, Benlioglu C, et al. SARS-CoV-2 infection in pregnancy: A systematic review and meta-analysis of clinical features and pregnancy outcomes. *EClinicalMedicine* 2020;25 doi: 10.1016/j.eclinm.2020.100446

31. Robertson NJ, Groenendaal F. Hypoxic-ischaemic brain injury. In: Rennie JM, ed. Rennie and Roberton's Textbook of Neonatology. 5th ed. London: Churchill Livingstone 2012:1114-55.

32. Say L, Chou D, Gemmill A, et al. Global causes of maternal death: a WHO systematic analysis. *Lancet Glob Health* 2014;2(6):e323-e33. doi: 10.1016/S2214-109X(14)70227-X

33. das Neves Martins Pires PH, Macaringue C, Abdirazak A, et al. Covid-19 pandemic impact on maternal and child health services access in Nampula, Mozambique: a mixed methods research. *BMC Health Serv Res* 2021;21(1):860. doi: 10.1186/s12913-021-06878-3

34. Hawley S, Ali MS, Berencsi K, et al. Sample size and power considerations for ordinary least squares interrupted time series analysis: a simulation study. *Clinical epidemiology* 2019;11:197-205. doi: 10.2147/CLEP.S176723

35. Kc A, Gurung R, Kinney MV, et al. Effect of the COVID-19 pandemic response on intrapartum care, stillbirth, and neonatal mortality outcomes in Nepal: a prospective observational study. *Lancet Glob Health* 2020;8(10):e1273-e81. doi: 10.1016/S2214-109X(20)30345-4

36. Thaddeus S, Maine D. Too far to walk: maternal mortality in context. *Soc Sci Med* 1994;38(8):1091-110. doi: 10.1016/0277-9536(94)90226-7 [published Online First: 1994/04/01]

For peer review only - http://bmjopen.bmj.com/site/about/guidelines.xhtml

Declaration of interests

The authors have no conflicts of interest to declare.

Acknowledgements

We are very grateful to the families at SMCH and KCH, and the staff members at both hospitals for their enthusiasm and commitment to the Neotree project, without which this work would not be possible.

Funders

We would like to thank the funders of this study. Dr S. R. Neal was awarded the International Child Health Group David Morley Elective Bursary for this elective project. Funders of the wider Neotree project, past and present, include the Wellcome Trust Digital Innovation Award (215742/Z/19/Z: PI: Heys), RCPCH, Naughton-Cliffe Mathews, UCL Grand Challenges and Global Engagement Fund, and the Healthcare Infection Society (SRG 201802004). Dr F. Fitzgerald is supported by the Academy of Medical Sciences and the funders of the Starter Grants for Clinical Lecturers scheme. This study and Drs M. Heys and F. Fitzgerald are further supported by the National Institute for Health Research Great Ormond Street Hospital Biomedical Research Centre. The funders had no role in study design, data collection and analysis, or preparation of this report. The views expressed are those of the authors and not necessarily those of the National Health Service (NHS), the NIHR or the UK Department of Health.

Data sharing statement

Data collected for the study cannot yet be made publicly available because primary analysis for the pilot implementation evaluation of the Neotree, as well as secondary analysis are ongoing. A goal of our pilot implementation is to establish an open-source anonymised research database of Neotree data to maximise the reach and utility for researchers aiming to improve outcomes for neonates in low-income settings. This database is under development and subject to negotiation with relevant Ministries of Health.

For peer review only

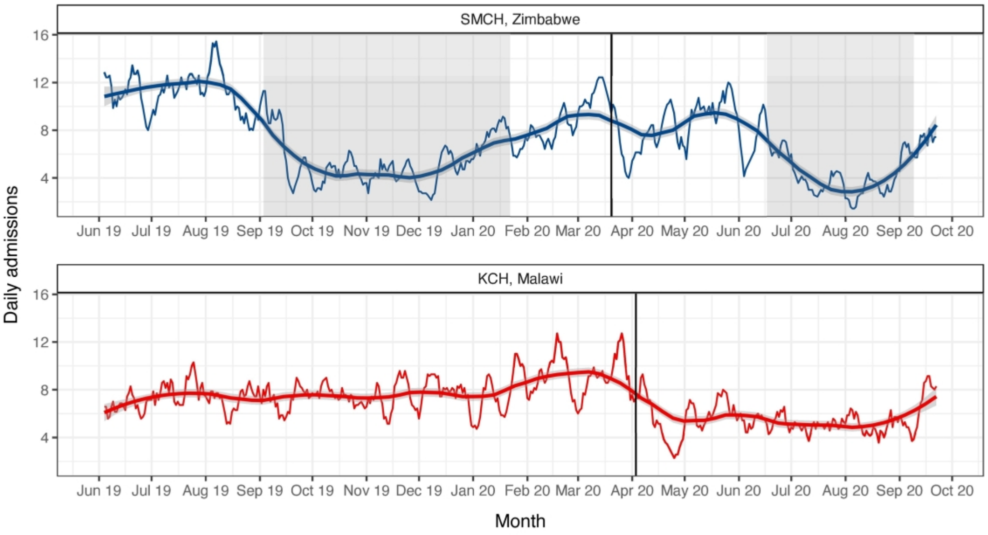

Figure 1: Trend in daily admissions to the neonatal unit
• The seven-day moving average of daily admission numbers has been plotted.
• Smoothed line: local regression (LOESS) model fitted on the seven-day moving average of daily admission numbers; shaded region: 95% confidence interval.
• Solid vertical line: first confirmed case of COVID-19 in each country.
• Shaded periods on SMCH, Zimbabwe panel: industrial action by doctors (3 September 2019 to 22 January 2020) and nurses (17 July 2020 to 9 September 2020).
• Counts based on all admission forms completed, irrespective of match status.
• SMCH: Sally Mugabe Central Hospital; KCH: Kamuzu Central Hospital

210x114mm (300 x 300 DPI)

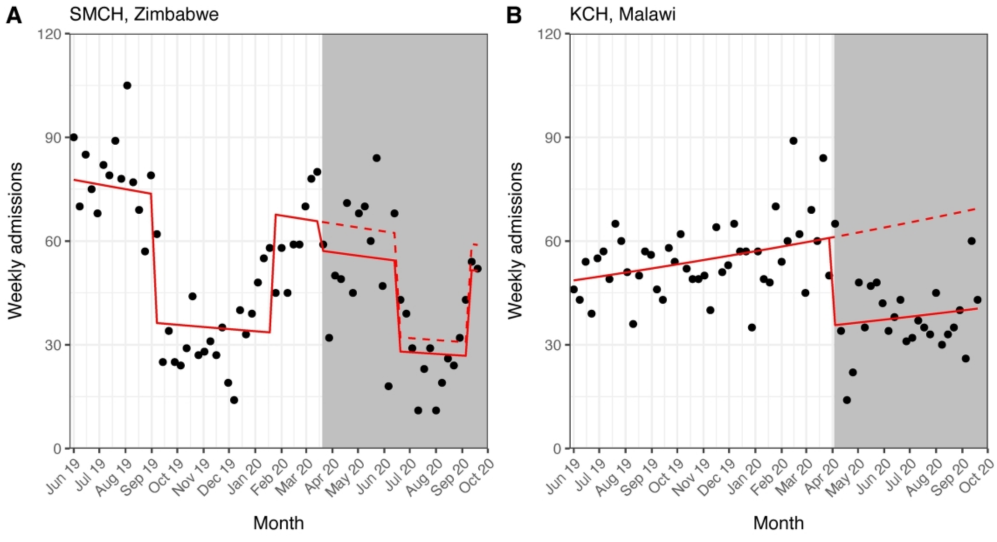

Figure 2: Interrupted time series for weekly admissions to the neonatal unit
• White background: pre-COVID-19 period; grey background: post-COVID-19 period.
• Solid line: predicted trend from negative binomial regression model; dashed line: counterfactual scenario.
• SMCH model (panel A) adjusted for doctors' and nurses' strike periods; KCH model (panel B) unadjusted.
• Counts based on all admission forms completed, irrespective of match status.
• SMCH: Sally Mugabe Central Hospital; KCH: Kamuzu Central Hospital

210x114mm (300 x 300 DPI)

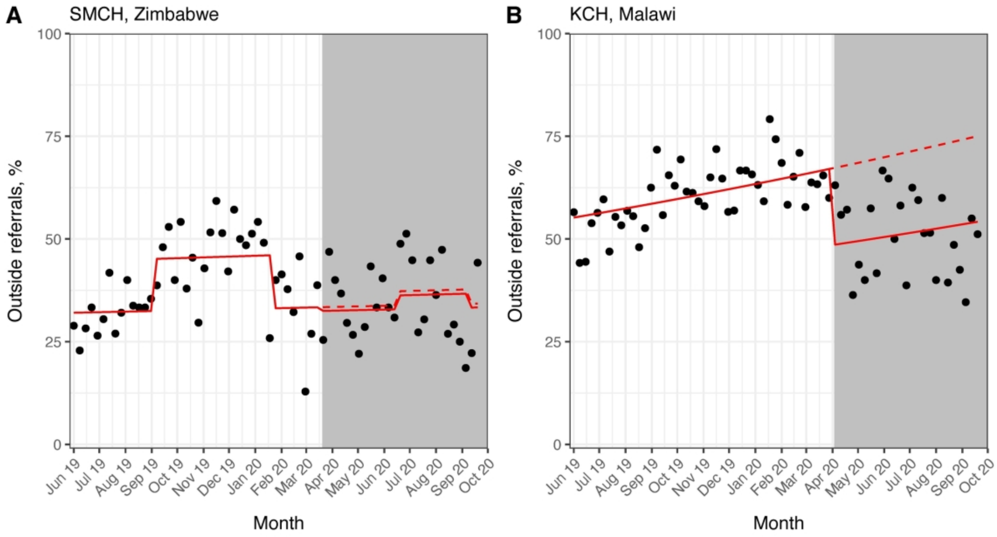

Figure 3: Interrupted time series for outside referrals to the neonatal unit
• White background: pre-COVID-19 period; grey background: post-COVID-19 period.
• Solid line: predicted trend from Poisson regression model; dashed line: counterfactual scenario.
• SMCH model (panel A) adjusted for doctors' and nurses' strike periods , KCH model (panel B) unadjusted.
• Data from all admission forms completed, irrespective of match status.
• SMCH: Sally Mugabe Central Hospital; KCH: Kamuzu Central Hospital

210x114mm (300 x 300 DPI)

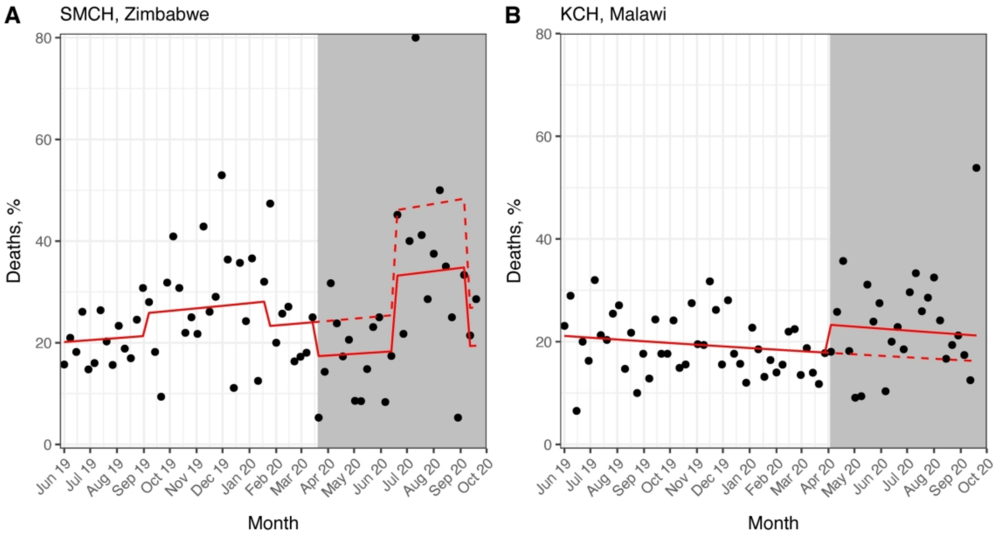

Figure 4: Interrupted time series for overall mortality
• White background: pre-COVID-19 period; grey background: post-COVID-19 period.
• Solid line: predicted trend from negative binomial regression model (SMCH, panel A) or Poisson regression model (KCH, panel B); dashed line: counterfactual scenario.
• SMCH model (panel A) adjusted for doctors' and nurses' strike periods; KCH model (panel B) unadjusted.
• Data from matched admission and outcome forms only.
• SMCH: Sally Mugabe Central Hospital; KCH: Kamuzu Central Hospital

210x114mm (300 x 300 DPI)

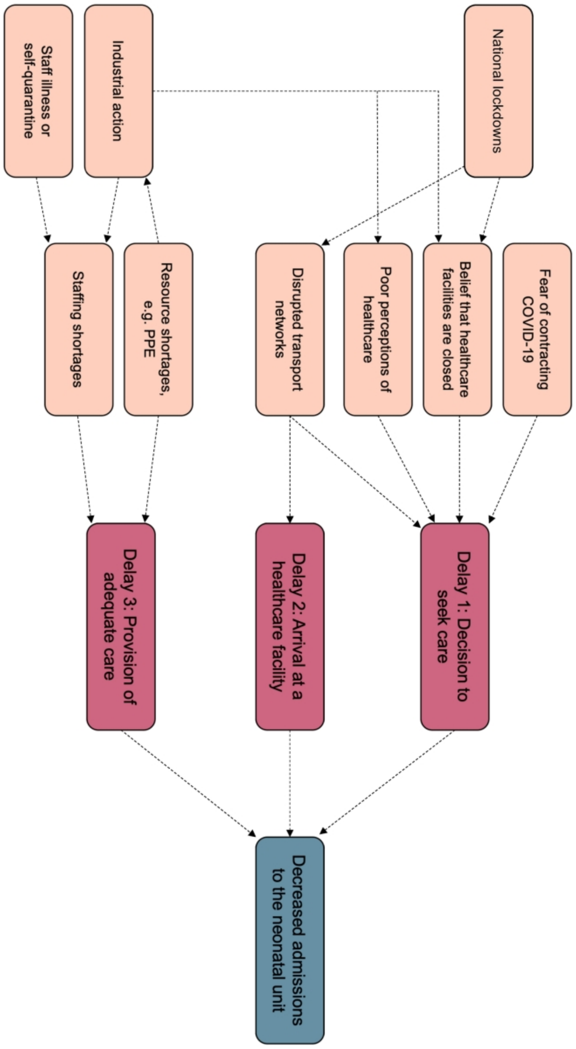

Figure 5: Possible factors influencing the decrease in admissions to the neonatal unit
• Delays (red boxes) derived from the “Three Delays” model of pregnancy-related mortality.36
• COVID-19: coronavirus disease 2019; PPE: personal protective equipment

209x297mm (300 x 300 DPI)

SUPPLEMENTARY FIGURES

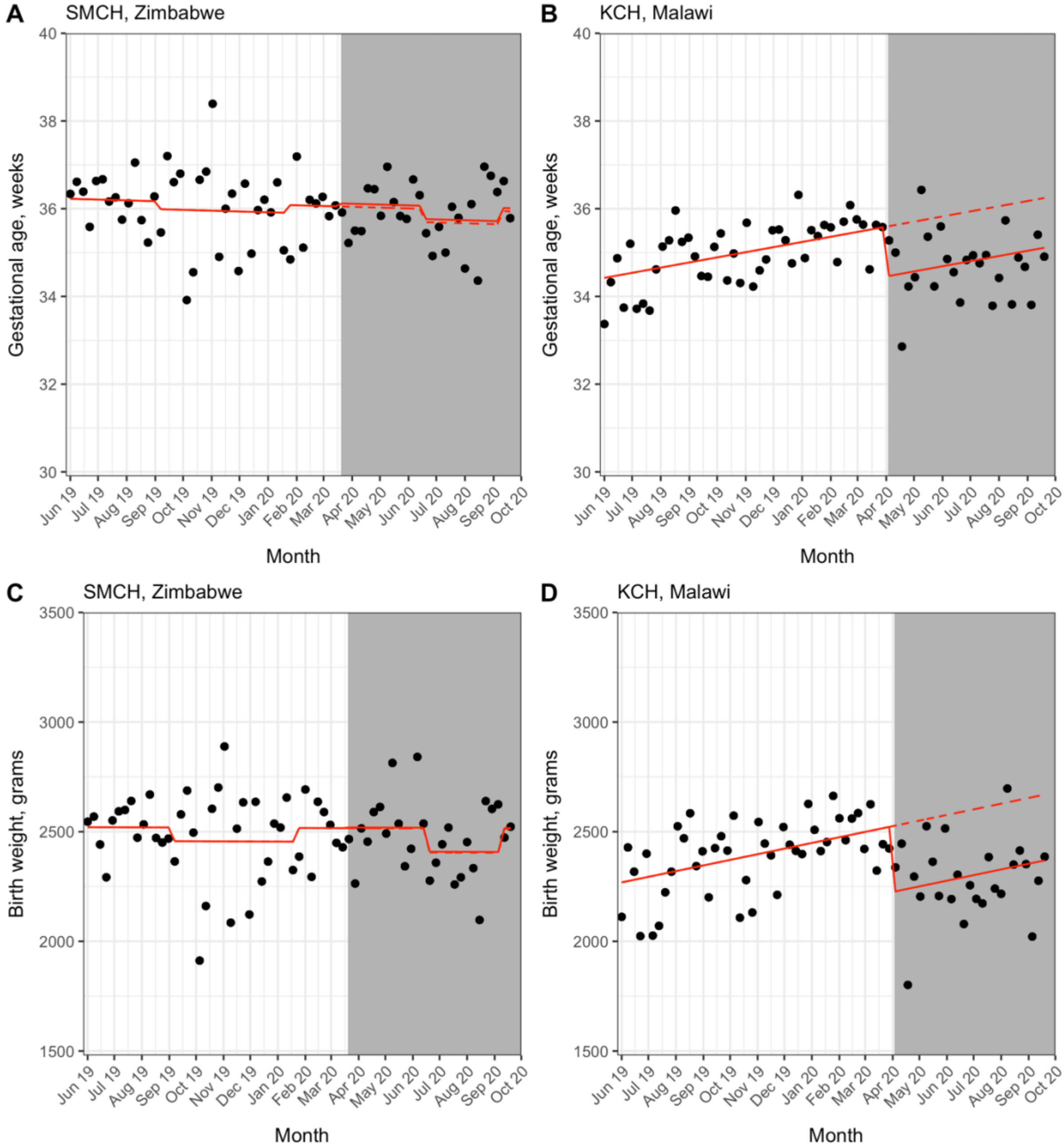

Supplementary Figure 1: Interrupted time series for gestational age and birth weight

- Data points represent weekly mean gestational age or birth weight to avoid overplotting.
- White background: pre-COVID-19 period; grey background: post-COVID-19 period.
- Solid line: predicted trend from linear regression model; dashed line: counterfactual scenario.
- SMCH models (panels A & C) adjusted for doctors' and nurses' strike periods, KCH models (panels B & D) unadjusted.
- Data from all admission forms completed, irrespective of match status.
- *SMCH: Sally Mugabe Central Hospital; KCH: Kamuzu Central Hospital*

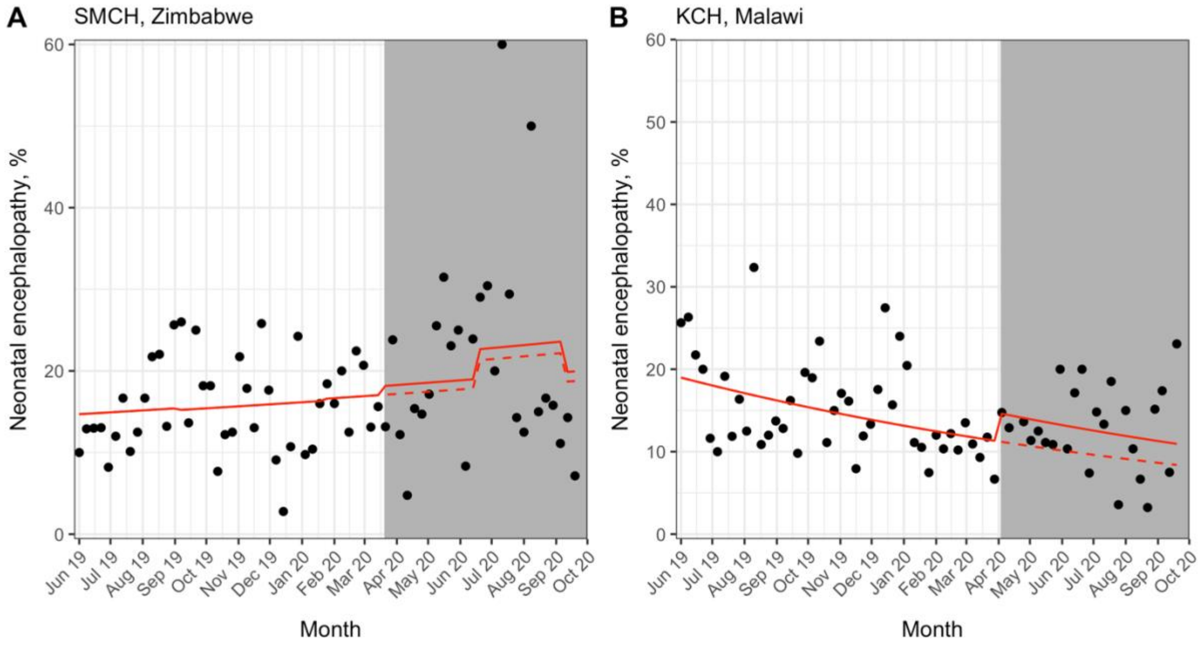

Supplementary Figure 2: Interrupted time series for prevalence of neonatal encephalopathy

- White background: pre-COVID-19 period; grey background: post-COVID-19 period.
- Solid line: predicted trend from Poisson regression model; dashed line: counterfactual scenario.
- SMCH model (panel A) adjusted for doctors' and nurses' strike periods, KCH model (panel B) unadjusted.
- Data from matched admission and outcome forms only.
- *SMCH: Sally Mugabe Central Hospital; KCH: Kamuzu Central Hospital*

For peer review only - http://bmjopen.bmj.com/site/about/guidelines.xhtml

APPENDIX 1: STROBE CHECKLIST

	Item No.	Recommendation	Page No.
**Title and abstract**	1	(*a*) Indicate the study's design with a commonly used term in the title or the abstract	1-2
		(*b*) Provide in the abstract an informative and balanced summary of what was done and what was found	2
**Introduction**			
Background/rationale	2	Explain the scientific background and rationale for the investigation being reported	4-5
Objectives	3	State specific objectives, including any prespecified hypotheses	5
**Methods**			
Study design	4	Present key elements of study design early in the paper	9-10
Setting	5	Describe the setting, locations, and relevant dates, including periods of recruitment, exposure, follow-up, and data collection	6-8
Participants	6	(*a*) *Cohort study*—Give the eligibility criteria, and the sources and methods of selection of participants. Describe methods of follow-up<br>*Case-control study*—Give the eligibility criteria, and the sources and methods of case ascertainment and control selection. Give the rationale for the choice of cases and controls<br>*Cross-sectional study*—Give the eligibility criteria, and the sources and methods of selection of participants	7
		(*b*) *Cohort study*—For matched studies, give matching criteria and number of exposed and unexposed<br>*Case-control study*—For matched studies, give matching criteria and the number of controls per case	n/a
Variables	7	Clearly define all outcomes, exposures, predictors, potential confounders, and effect modifiers. Give diagnostic criteria, if applicable	8
Data sources/ measurement	8*	For each variable of interest, give sources of data and details of methods of assessment (measurement). Describe comparability of assessment methods if there is more than one group	7-8
Bias	9	Describe any efforts to address potential sources of bias	9-10
Study size	10	Explain how the study size was arrived at	7
Quantitative variables	11	Explain how quantitative variables were handled in the analyses. If applicable, describe which groupings were chosen and why	9-10

For peer review only - http://bmjopen.bmj.com/site/about/guidelines.xhtml

Statistical methods	12	(*a*) Describe all statistical methods, including those used to control for confounding	9-10
		(*b*) Describe any methods used to examine subgroups and interactions	9-10, Appendix 5
		(*c*) Explain how missing data were addressed	9-10, Appendix 4
		(*d*) *Cohort study*—If applicable, explain how loss to follow-up was addressed<br>*Case-control study*—If applicable, explain how matching of cases and controls was addressed<br>*Cross-sectional study*—If applicable, describe analytical methods taking account of sampling strategy	9
		(*e*) Describe any sensitivity analyses	9-10, Appendix 5
**Results**			
Participants	13*	(a) Report numbers of individuals at each stage of study—eg numbers potentially eligible, examined for eligibility, confirmed eligible, included in the study, completing follow-up, and analysed	12, Appendix 3
		(b) Give reasons for non-participation at each stage	9, Appendix 3
		(c) Consider use of a flow diagram	Appendix 3
Descriptive data	14*	(a) Give characteristics of study participants (eg demographic, clinical, social) and information on exposures and potential confounders	12-13, Appendix 5
		(b) Indicate number of participants with missing data for each variable of interest	Appendix 4
		(c) *Cohort study*—Summarise follow-up time (eg, average and total amount)	7
Outcome data	15*	*Cohort study*—Report numbers of outcome events or summary measures over time	12-15
		Case-control study—Report numbers in each exposure category, or summary measures of exposure	n/a
		Cross-sectional study—Report numbers of outcome events or summary measures	n/a
Main results	16	(*a*) Give unadjusted estimates and, if applicable, confounder-adjusted estimates and their precision (eg, 95% confidence interval). Make clear which confounders were adjusted for and why they were included	12-15
		(*b*) Report category boundaries when continuous variables were categorized	12-15
		(*c*) If relevant, consider translating estimates of relative risk into absolute risk for a meaningful time period	12-15

For peer review only - http://bmjopen.bmj.com/site/about/guidelines.xhtml

For peer review only

Other analyses	17	Report other analyses done—eg analyses of subgroups and interactions, and sensitivity analyses	Appendix 5, Appendix 6
**Discussion**			
Key results	18	Summarise key results with reference to study objectives	16
Limitations	19	Discuss limitations of the study, taking into account sources of potential bias or imprecision. Discuss both direction and magnitude of any potential bias	18-19
Interpretation	20	Give a cautious overall interpretation of results considering objectives, limitations, multiplicity of analyses, results from similar studies, and other relevant evidence	16-19
Generalisability	21	Discuss the generalisability (external validity) of the study results	16-19
**Other information**			
Funding	22	Give the source of funding and the role of the funders for the present study and, if applicable, for the original study on which the present article is based	26

Adapted from: von Elm E, Altman DG, Egger M, Pocock SJ, Gøtzsche PC, et al. (2007) The Strengthening the Reporting of Observational Studies in Epidemiology (STROBE) Statement: Guidelines for Reporting Observational Studies. PLOS Medicine 4(10): e296. https://doi.org/10.1371/journal.pmed.0040296

For peer review only

For peer review only - http://bmjopen.bmj.com/site/about/guidelines.xhtml

APPENDIX 2: ETHICAL APPROVAL

Ethical approval for this study was granted by the following ethics committees.

Table A2.1: Ethical approval

Committee	Reference
United Kingdom	
University College London Research Ethics Committee	17123/001
Malawi	
College of Medicine Research and Ethics Committee	P.01/20/2909
Zimbabwe	
Medical Research Council of Zimbabwe	MRCZ/A/2570
Joint Research Ethics Committee for the University of Zimbabwe, College of Health Sciences and Parirenyatwa Group of Hospitals	JREC/327/19
Biomedical Research and Training Institute Institutional Review Board	AP155/2020
Sally Mugabe (Harare) Central Hospital Ethics Committee	071119/64

For peer review only

For peer review only - http://bmjopen.bmj.com/site/about/guidelines.xhtml

APPENDIX 3: FLOW DIAGRAMS OF RECORD INCLUSION

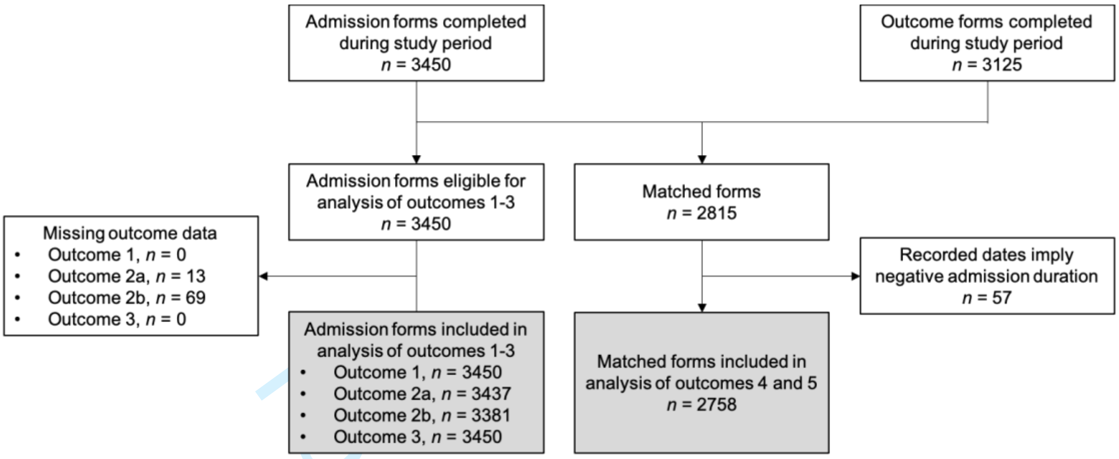

Figure A3.1: Flow diagram of record inclusion for Sally Mugabe Central Hospital, Zimbabwe

- Outcome 1: number of admissions; outcome 2a: gestational age; outcome 2b: birth weight; outcome 3: source of admission; outcome 4: prevalence of neonatal encephalopathy; outcome 5: overall mortality rate

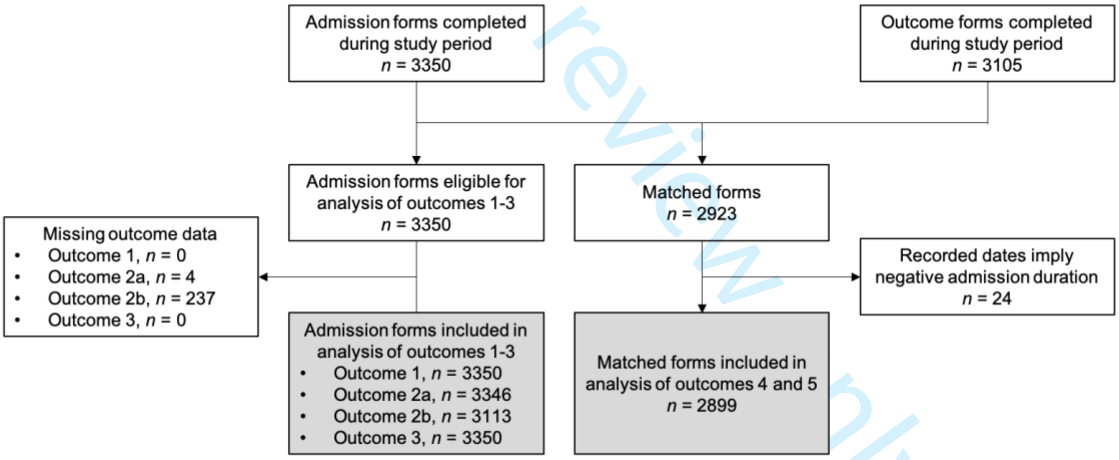

Figure A3.2: Flow diagram of record inclusion for Kamuzu Central Hospital, Malawi

- Outcome 1: number of admissions; outcome 2a: gestational age; outcome 2b: birth weight; outcome 3: source of admission; outcome 4: prevalence of neonatal encephalopathy; outcome 5: overall mortality rate

For peer review only - http://bmjopen.bmj.com/site/about/guidelines.xhtml

APPENDIX 4: MISSING DATA

The table below shows the number of participants with missing data for each outcome and the number of participants remaining for each analysis after pairwise deletion of missing values.

Table A4.1: Summary of missing data

Characteristics	*n* missing (%)		*n* remaining*	
	SMCH	KCH	SMCH	KCH
Gestational age	13 (0·4)	4 (0·1)	3437 (99·6)	3346 (99·9)
Birth weight	69 (2·0)	237 (7·1)	3381 (98·0)	3113 (92·9)
Source of admission	0 (0·0)	0 (0·0)	3450 (100·0)	3350 (100·0)
Neonatal encephalopathy	0 (0·0)	0 (0·0)	2758 (100·0)†	2899 (100·0)†
Death	0 (0·0)	0 (0·0)	2758 (100·0)†	2899 (100·0)†

- * Remaining for analysis after pairwise deletion.
- † Only matched admission and outcome forms considered for analysis of neonatal encephalopathy and death.
- *SMCH: Sally Mugabe Central Hospital; KCH: Kamuzu Central Hospital, Malawi*

For peer review only

APPENDIX 5: FURTHER REGRESSION ANALYSIS RESULTS

Outcome 1: Admissions to the neonatal unit

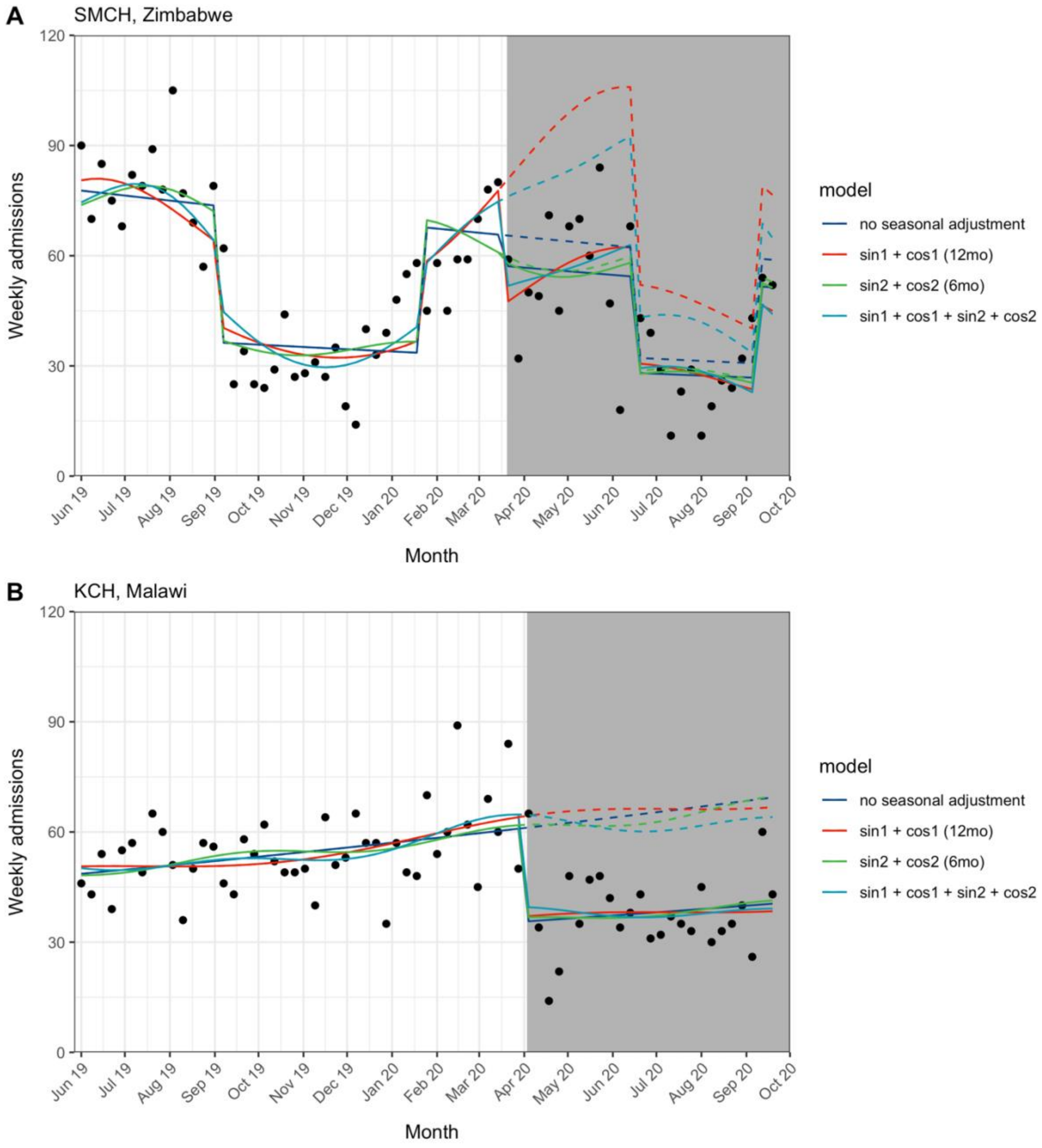

Figure A5.1.1: Interrupted time series for weekly admissions to the neonatal unit, negative binomial regression models with and without seasonal adjustment

Table A5.1.1: SMCH, Zimbabwe; Results of the models with and without adjustment for seasonality

Model*		BIC	LR statistic†	Df	*p*-value
0	Negative binomial, unadjusted for seasonality	585·6	ref		
1	Negative binomial, cosine function with 6-month period	588·9	5·23	2	0·07
2	Negative binomial, cosine function with 12-month period	592·9	1·22	2	0·54
3	Negative binomial, mixture of two cosine functions with 6-month and 12-month periods	595·6	6·96	4	0·13

- * All models adjusted for the doctors' and nurses' strike periods.
- † Likelihood ratio χ^2-test compared to Model 0.

For peer review only

Table A5.1.2: SMCH, Zimbabwe; Negative binomial model, unadjusted for seasonality (Model 0)

	Coef	SE	Exp	95% CI	*p*-value
Intercept	*4·35*	*0·09*			
Post-COVID-19 period, yes	-0·14	0·15	0·87	0·65 – 1·17	0·37
Study time elapsed, weeks	-0·00	0·00	1·00	0·99 – 1·00	0·25
Doctors' strike period, yes	-0·70	0·10	0·49	0·41 – 0·60	< 0·001
Nurses' strike period, yes	-0·66	0·13	0·52	0·41 – 0·66	< 0·001

Table A5.1.3: KCH, Malawi; Results of the models with and without adjustment for seasonality

Model		BIC	LR statistic†	Df	*p*-value
0	Negative binomial, unadjusted for seasonality	534·5	ref		
1	Negative binomial, cosine function with 6-month period	541·5	1·40	2	0·50
2	Negative binomial, cosine function with 12-month period	542·4	0·52	2	0·77
3	Negative binomial, mixture of two cosine functions with 6-month and 12-month periods	549·1	2·36	4	0·67

- † Likelihood ratio χ^2-test compared to Model 0.

Table A5.1.4: KCH, Malawi; Negative binomial model, unadjusted for seasonality (Model 0)

	Coef	SE	Exp	95% CI	*p*-value
Intercept	*3·88*	*0·06*			
Post-COVID-19 period, yes	-0·54	0·10	0·58	0·48 – 0·70	< 0·001
Study time elapsed, weeks	0·01	0·00	1·01	1·00 – 1·01	0·022

For peer review only - http://bmjopen.bmj.com/site/about/guidelines.xhtml

Outcome 2: Gestational age at birth and birth weight

a. Gestational age at birth

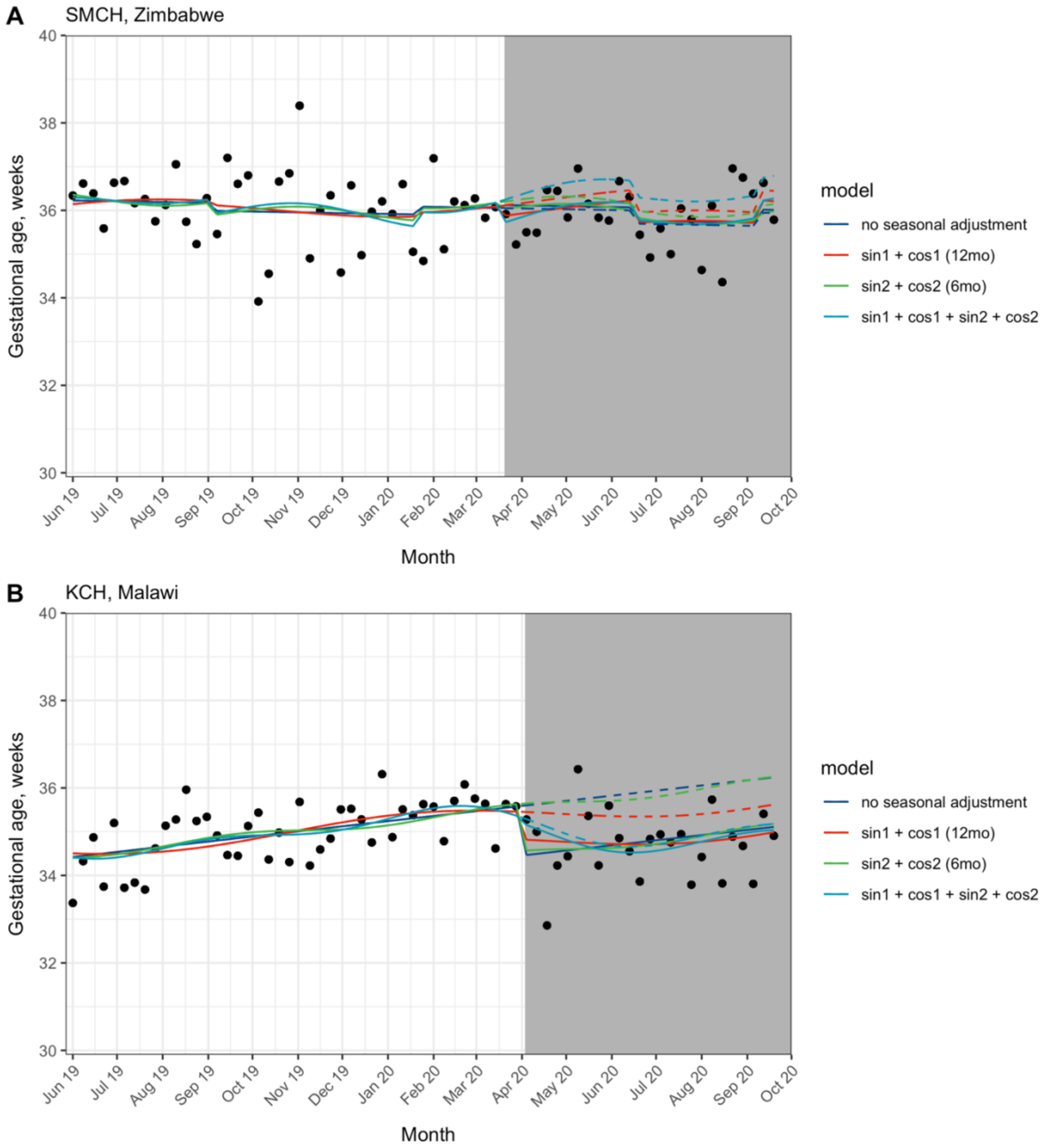

Figure A5.2.1: Interrupted time series for gestational age at birth, linear regression models with and without seasonal adjustment

Table A5.2.1: SMCH, Zimbabwe; Results of the models with and without adjustment for seasonality

	Model*	BIC	Deviance†	Df	*p*-value
0	Linear, unadjusted for seasonality	19851·6	ref		
1	Linear, cosine function with 6-month period	19866·6	24·0	2	0·53
2	Linear, cosine function with 12-month period	19867·0	15·8	2	0·65
3	Linear, mixture of two cosine functions with 6-month and 12-month periods	19881·4	50·9	4	0·60

- * All models adjusted for the doctors' and nurses' strike periods.
- † χ^2-test compared to Model 0.

Table A5.2.2: SMCH, Zimbabwe; Linear model, unadjusted for seasonality (Model 0)

	Coef	SE	95% CI	*p*-value
Intercept	*36·23*	*0·15*		
Post-COVID-19 period, yes	0·07	0·29	-0·50 – 0·64	0·81
Study time elapsed, weeks	-0·00	0·01	-0·02 – 0·01	0·52
Doctors' strike period, yes	-0·18	0·20	-0·58 – 0·22	0·38
Nurses' strike period, yes	-0·30	0·29	-0·87 – 0·27	0·30

Table A5.2.3: KCH, Malawi; Results of the models with and without adjustment for seasonality

	Model	BIC	Deviance†	Df	*p*-value
0	Linear, unadjusted for seasonality	18631·8	ref		
1	Linear, cosine function with 6-month period	18645·2	43·2	2	0·24
2	Linear, cosine function with 12-month period	18647·2	12·9	2	0·65
3	Linear, mixture of two cosine functions with 6-month and 12-month periods	18658·4	89·0	4	0·21

- † χ^2-test compared to Model 0.

Table A5.2.4: KCH, Malawi; Linear model, unadjusted for seasonality (Model 0)

	Coef	SE	95% CI	*p*-value
Intercept	*34·42*	*0·15*		
Post-COVID-19 period, yes	-1·14	0·25	-1·62 – -0·65	< 0·001
Study time elapsed, weeks	0·03	0·01	0·02 – 0·04	< 0·001

For peer review only

b. Birth weight

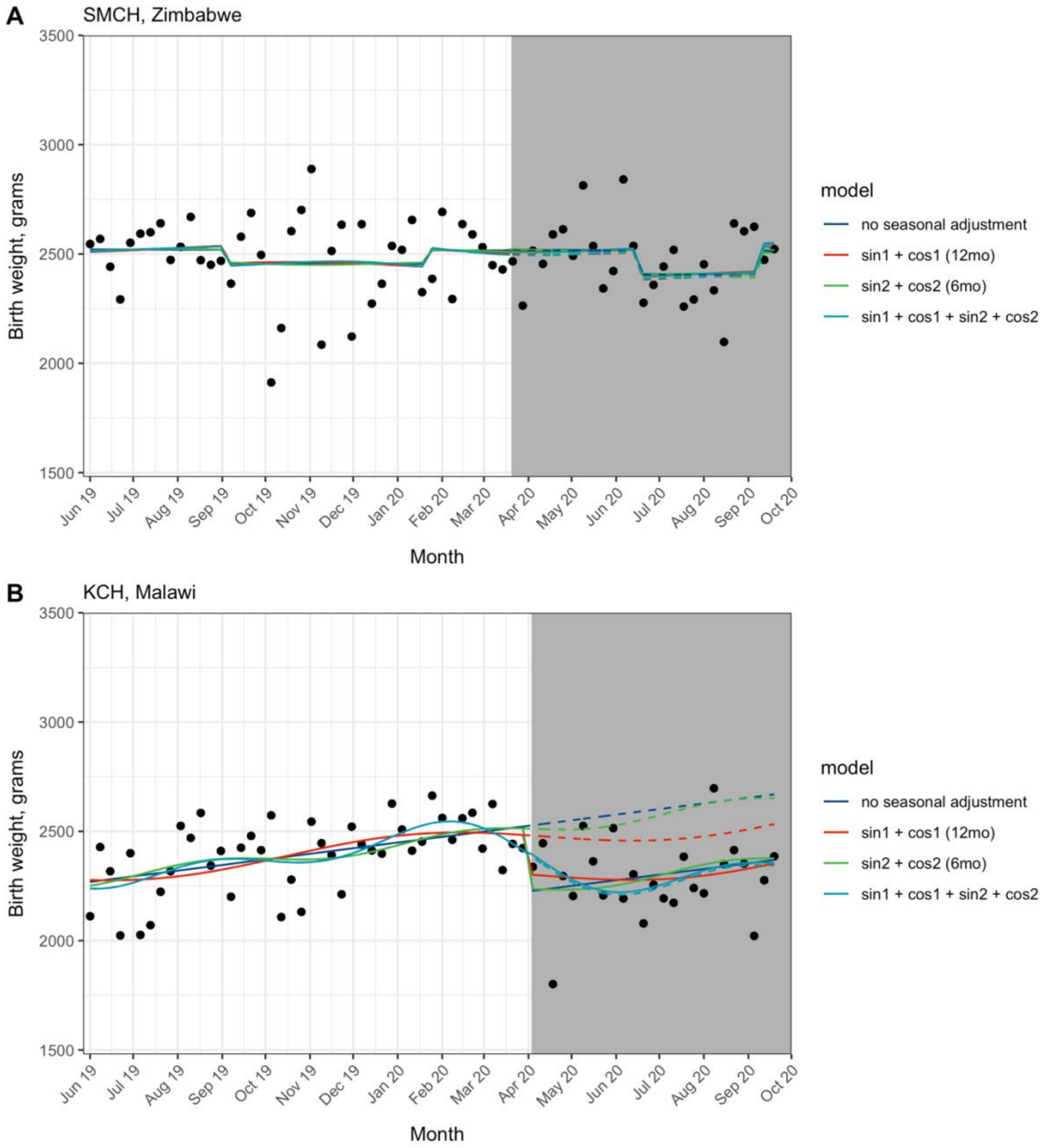

Figure A5.2.2: Interrupted time series for birth weight, linear regression models with and without seasonal adjustment

For peer review only - http://bmjopen.bmj.com/site/about/guidelines.xhtml

Table A5.2.5: SMCH, Zimbabwe; Results of the models with and without adjustment for seasonality

Model*		BIC	Deviance†	Df	*p*-value
0	Linear, unadjusted for seasonality	55660·9	ref		
1	Linear, cosine function with 6-month period	55676·8	289194	2	0·84
2	Linear, cosine function with 12-month period	55677·1	28641	2	0·98
3	Linear, mixture of two cosine functions with 6-month and 12-month periods	55693·0	351647	4	0·98

- * All models adjusted for the doctors' and nurses' strike periods.
- † χ^2-test compared to Model 0.

Table A5.2.6: SMCH, Zimbabwe; Linear model, unadjusted for seasonality (Model 0)

	Coef	SE	95% CI	*p*-value
Intercept	*2520·71*	*31·89*		
Post-COVID-19 period, yes	3·38	61·42	-117·0 – 123·8	0·96
Study time elapsed, weeks	-0·11	1·38	-2·8 – 2·6	0·94
Doctors' strike period, yes	-62·52	42·92	-146·6 – 21·6	0·15
Nurses' strike period, yes	-109·4	61·0	-229·0 – 10·2	0·07

Table A5.2.7: KCH, Malawi; Results of the models with and without adjustment for seasonality

Model		BIC	Deviance†	Df	*p*-value
0	Linear, unadjusted for seasonality	51050·5	ref		
1	Linear, cosine function with 6-month period	51064·1	1922568	2	0·29
2	Linear, cosine function with 12-month period	51065·2	1105739	2	0·49
3	Linear, mixture of two cosine functions with 6-month and 12-month periods	51073·9	6744491	4	0·07

- † χ^2-test compared to Model 0.

Table A5.2.8: KCH, Malawi; Linear model, unadjusted for seasonality (Model 0)

	Coef	SE	95% CI	*p*-value
Intercept	*2268·96*	*36·02*		
Post-COVID-19 period, yes	-299·89	57·34	-412·3 – -187·5	< 0·001
Study time elapsed, weeks	5·88	1·37	3·2 – 8·6	< 0·001

For peer review only

Outcome 3: Source of admission referral

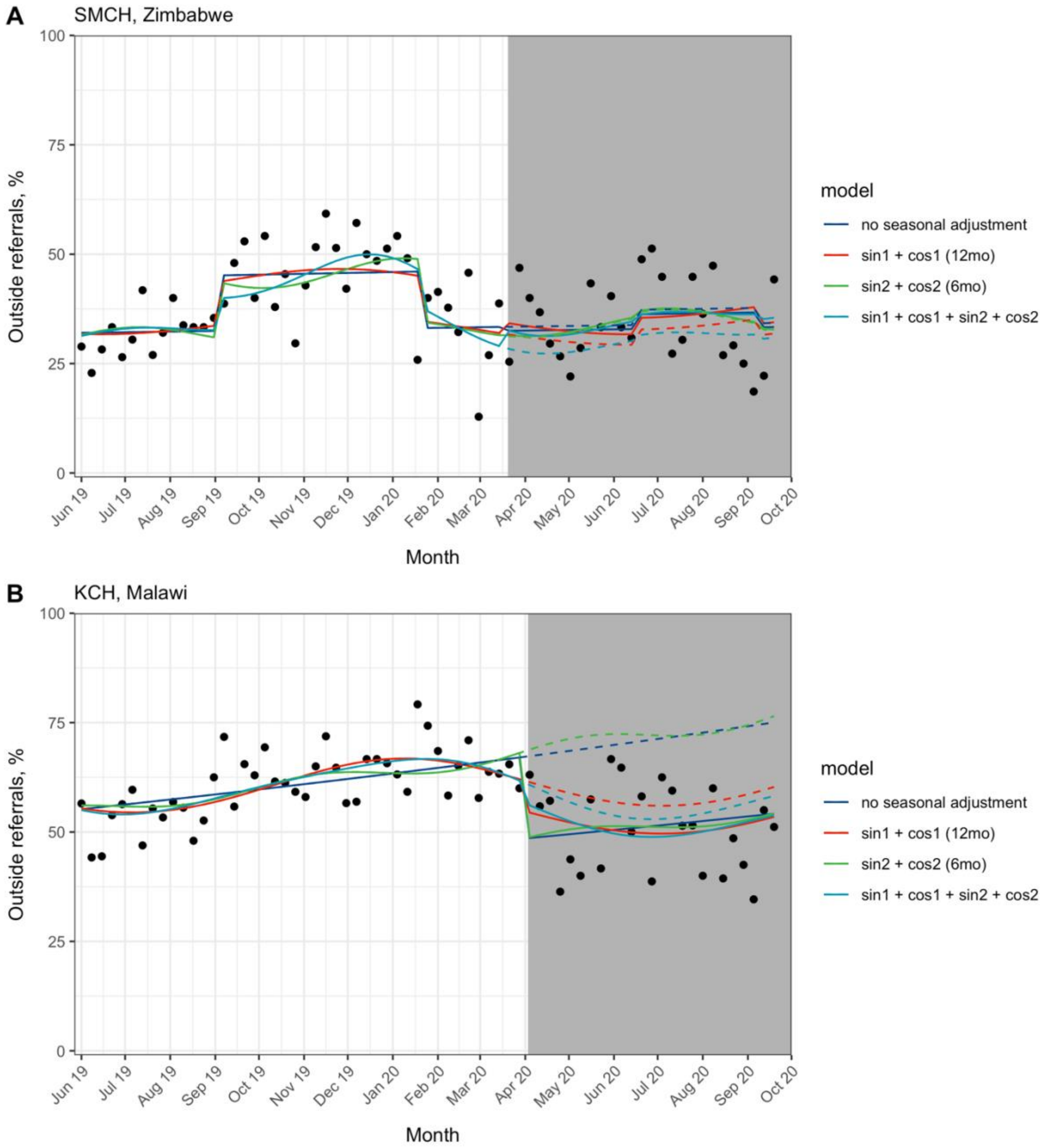

Figure A5.3.1: Interrupted time series for outside referrals to the neonatal unit, Poisson regression models with and without seasonal adjustment

Table A5.3.1: SMCH, Zimbabwe; Results of the models with and without adjustment for seasonality

Model*		BIC	Deviance†	Df	*p*-value
0	Poisson, unadjusted for seasonality	406·3	ref		
1	Poisson, cosine function with 6-month period	414·2	0·56	2	0·76
2	Poisson, cosine function with 12-month period	412·9	1·85	2	0·40
3	Poisson, mixture of two cosine functions with 6-month and 12-month periods	419·8	3·42	4	0·49

- * All models adjusted for the doctors' and nurses' strike periods.
- † χ^2-test compared to Model 0.

Table A5.3.2: SMCH, Zimbabwe; Poisson model, unadjusted for seasonality (Model 0)

	Coef	SE	Exp	95% CI	*p*-value
Intercept	*-1·14*	*0·06*			
Post-COVID-19 period, yes	-0·03	0·12	0·97	0·77 – 1·22	0·81
Study time elapsed, weeks	0·00	0·00	1·00	1·00 – 1·01	0·70
Doctors' strike period, yes	0·33	0·07	1·39	1·20 – 1·61	< 0·001
Nurses' strike period, yes	0·10	0·11	1·10	0·88 – 1·37	0·39

Table A5.3.3: KCH, Malawi; Results of the models with and without adjustment for seasonality

Model		BIC	Deviance†	Df	*p*-value
0	Poisson, unadjusted for seasonality	398·0	ref		
1	Poisson, cosine function with 6-month period	403·3	3·23	2	0·20
2	Poisson, cosine function with 12-month period	405·9	0·58	2	0·75
3	Poisson, mixture of two cosine functions with 6-month and 12-month periods	411·5	3·43	4	0·49

- † χ^2-test compared to Model 0.

Table A5.3.4: KCH, Malawi; Poisson model, unadjusted for seasonality (Model 0)

	Coef	SE	Exp	95% CI	*p*-value
Intercept	*-0·59*	*0·05*			
Post-COVID-19 period, yes	-0·33	0·08	0·72	0·61 – 0·85	< 0·001
Study time elapsed, weeks	0·01	0·00	1·01	1·00 – 1·01	0·020

For peer review only - http://bmjopen.bmj.com/site/about/guidelines.xhtml
For peer review only

Outcome 4: Prevalence of neonatal encephalopathy

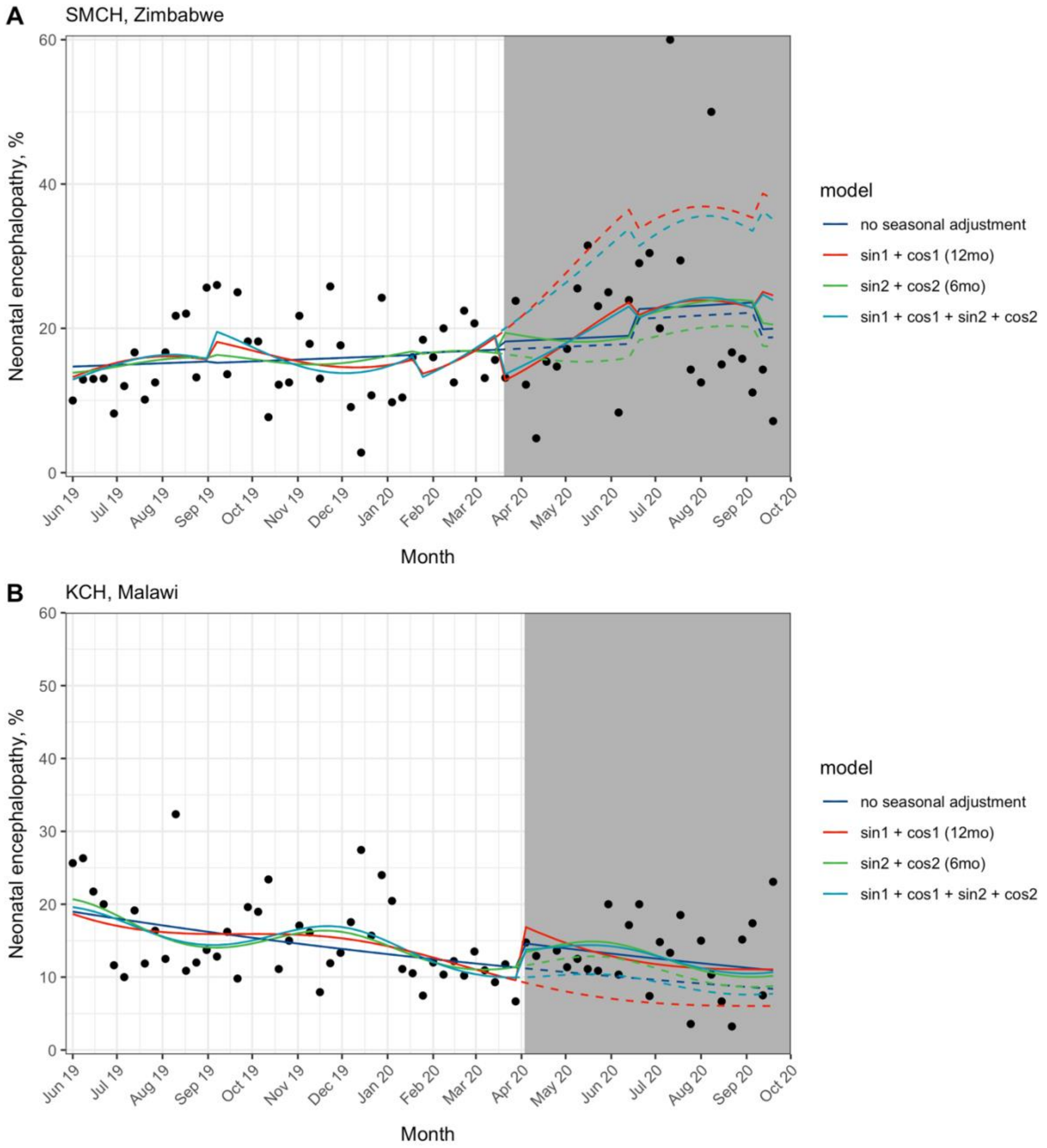

Figure A5.4.1: Interrupted time series for prevalence of neonatal encephalopathy, Poisson regression models with and without seasonal adjustment

Table A5.4.1: SMCH, Zimbabwe; Results of the models with and without adjustment for seasonality

Model*		BIC	Deviance†	Df	*p*-value
0	Poisson, unadjusted for seasonality	333·5	ref		
1	Poisson, cosine function with 6-month period	336·9	5·06	2	0·08
2	Poisson, cosine function with 12-month period	341·5	0·45	2	0·80
3	Poisson, mixture of two cosine functions with 6-month and 12-month periods	345·0	5·39	4	0·25

- * All models adjusted for the doctors' and nurses' strike periods.
- † χ^2-test compared to Model 0.

For peer review only

Table A5.4.2: SMCH, Zimbabwe; Poisson model, unadjusted for seasonality (Model 0)

	Coef	SE	Exp	95% CI	*p*-value
Intercept	*-1·92*	*0·10*			
Post-COVID-19 period, yes	0·06	0·18	1·06	0·74 – 1·52	0·74
Study time elapsed, weeks	0·00	0·00	1·00	1·00 – 1·01	0·39
Doctors' strike period, yes	-0·02	0·13	0·99	0·77 – 1·26	0·91
Nurses' strike period, yes	0·18	0·18	1·19	0·84 – 1·69	0·33

Table A5.4.3: KCH, Malawi; Results of the models with and without adjustment for seasonality

Model		BIC	Deviance†	Df	*p*-value
0	Poisson, unadjusted for seasonality	302·3	ref		
1	Poisson, cosine function with 6-month period	308·9	1·83	2	0·40
2	Poisson, cosine function with 12-month period	307·5	3·29	2	0·19
3	Poisson, mixture of two cosine functions with 6-month and 12-month periods	315·3	3·92	4	0·42

- † χ^2-test compared to Model 0.

Table A5.4.4: KCH, Malawi; Poisson model, unadjusted for seasonality (Model 0)

	Coef	SE	Exp	95% CI	*p*-value
Intercept	*-1·66*	*0·10*			
Post-COVID-19 period, yes	0·27	0·19	1·31	0·91 – 1·88	0·15
Study time elapsed, weeks	-0·01	0·00	0·99	0·99 – 1·00	0·005

For peer review only - http://bmjopen.bmj.com/site/about/guidelines.xhtml

Outcome 5: Overall mortality

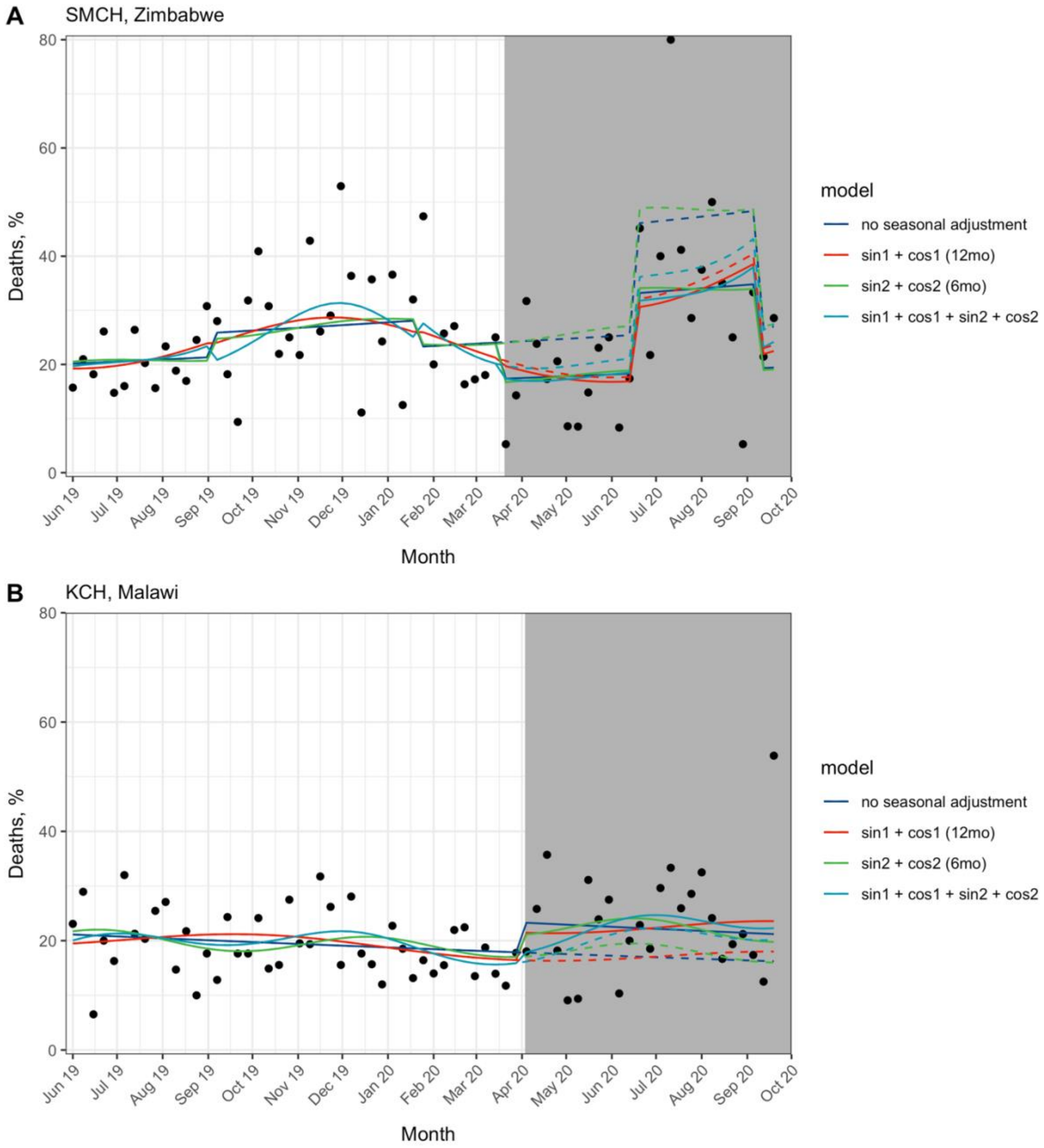

Figure A5.5.1: Interrupted time series for overall mortality, negative binomial regression models (SMCH, Zimbabwe) and Poisson regression models (KCH, Malawi) with and without seasonal adjustment

For peer review only - http://bmjopen.bmj.com/site/about/guidelines.xhtml

Table A5.5.1: SMCH, Zimbabwe; Results of the models with and without adjustment for seasonality

Model*		BIC	LR statistic†	Df	*p*-value
0	Negative binomial, unadjusted for seasonality	373·0	ref		
1	Negative binomial, cosine function with 6-month period	379·2	2·32	2	0·31
2	Negative binomial, cosine function with 12-month period	381·2	0·26	2	0·88
3	Negative binomial, mixture of two cosine functions with 6-month and 12-month periods	385·9	4·02	4	0·40

- * All models adjusted for the doctors' and nurses' strike periods.
- † Likelihood ratio χ^2-test compared to Model 0.

Table A5.5.2: SMCH, Zimbabwe; Negative binomial model, unadjusted for seasonality (Model 0)

	Coef	SE	Exp	95% CI	*p*-value
Intercept	*-1·60*	*0·09*			
Post-COVID-19 period, yes	-0·33	0·17	0·72	0·52 – 1·00	0·05
Study time elapsed, weeks	0·00	0·00	1·00	1·00 – 1·01	0·24
Doctors' strike period, yes	0·19	0·10	1·21	0·99 – 1·48	0·07
Nurses' strike period, yes	0·59	0·16	1·81	1·31 – 2·49	< 0·001

Table A5.5.3: KCH, Malawi; Results of the models with and without adjustment for seasonality

Model		BIC	Deviance†	Df	*p*-value
0	Poisson, unadjusted for seasonality	343·1	ref		
1	Poisson, cosine function with 6-month period	349·7	1·86	2	0·39
2	Poisson, cosine function with 12-month period	349·7	1·90	2	0·39
3	Poisson, mixture of two cosine functions with 6-month and 12-month periods	355·4	4·69	4	0·32

- † χ^2-test compared to Model 0.

Table A5.5.4: KCH, Malawi; Poisson model, unadjusted for seasonality (Model 0)

	Coef	SE	Exp	95% CI	*p*-value
Intercept	*-1·56*	*0·09*			
Post-COVID-19 period, yes	0·27	0·15	1·31	0·97 – 1·76	0·08
Study time elapsed, weeks	-0·00	0·00	1·00	0·99 – 1·00	0·29

For peer review only - http://bmjopen.bmj.com/site/about/guidelines.xhtml

APPENDIX 6: ADDITIONAL ANALYSES

Mode of delivery of admitted neonates

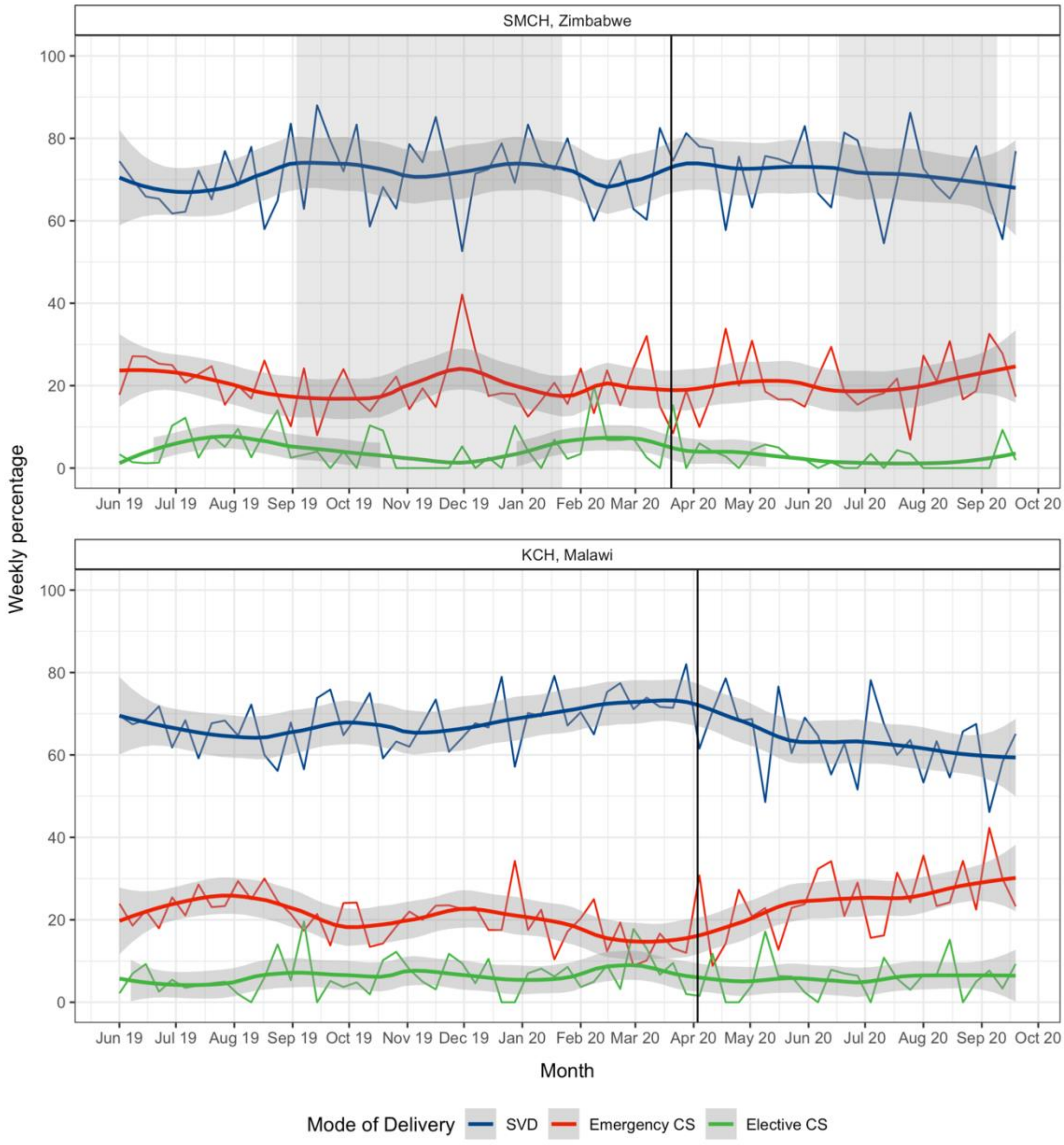

Figure A6.1.1: Trend in mode of delivery of admitted neonates per week

- Only SVD, emergency CS and elective CS displayed here to avoid overplotting.
- Smoothed line: local regression (LOESS) model; shaded region: 95% confidence interval.
- Solid vertical line: first confirmed case of COVID-19 in each country.
- Shaded periods on SMCH, Zimbabwe panel: industrial action by doctors (3 September 2019 to 22 January 2020) and nurses (17 July 2020 to 9 September 2020).
- Counts based on all admission forms completed, irrespective of match status.
- *SMCH: Sally Mugabe Central Hospital; KCH: Kamuzu Central Hospital; SVD: spontaneous vaginal delivery; CS: caesarean section*

For peer review only - http://bmjopen.bmj.com/site/about/guidelines.xhtml

Reason for elective caesarean section

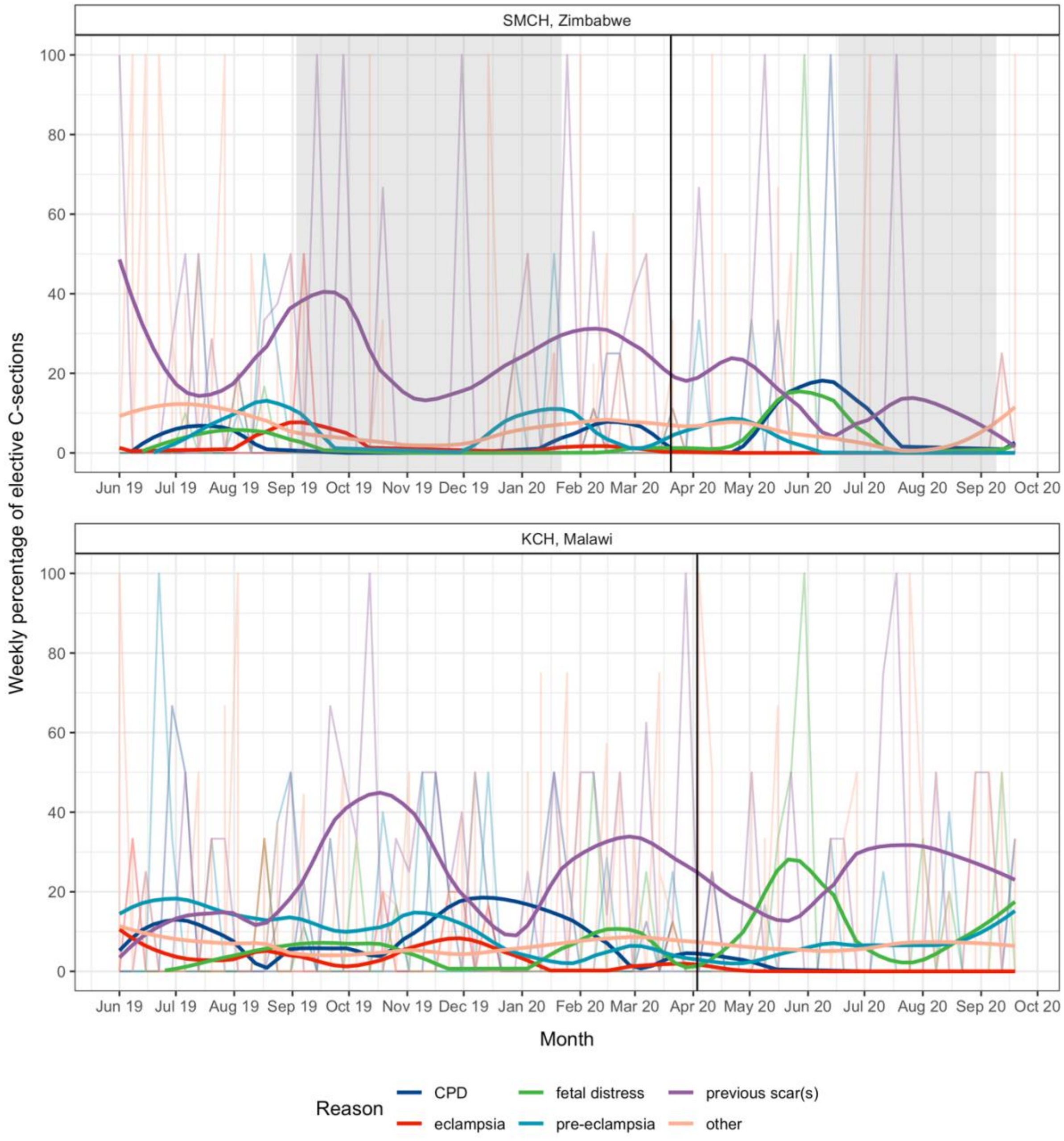

Figure A6.2.1: Trend in reason for elective caesarean section per week

- Smoothed line: local regression (LOESS) model; 95% confidence interval not presented to avoid overplotting.
- Solid vertical line: first confirmed case of COVID-19 in each country.
- Shaded periods on SMCH, Zimbabwe panel: industrial action by doctors (3 September 2019 to 22 January 2020) and nurses (17 July 2020 to 9 September 2020).
- Counts based on all admission forms completed, irrespective of match status.
- *SMCH: Sally Mugabe Central Hospital; KCH: Kamuzu Central Hospital; CPD: cephalopelvic disproportion*

Reason for emergency caesarean section

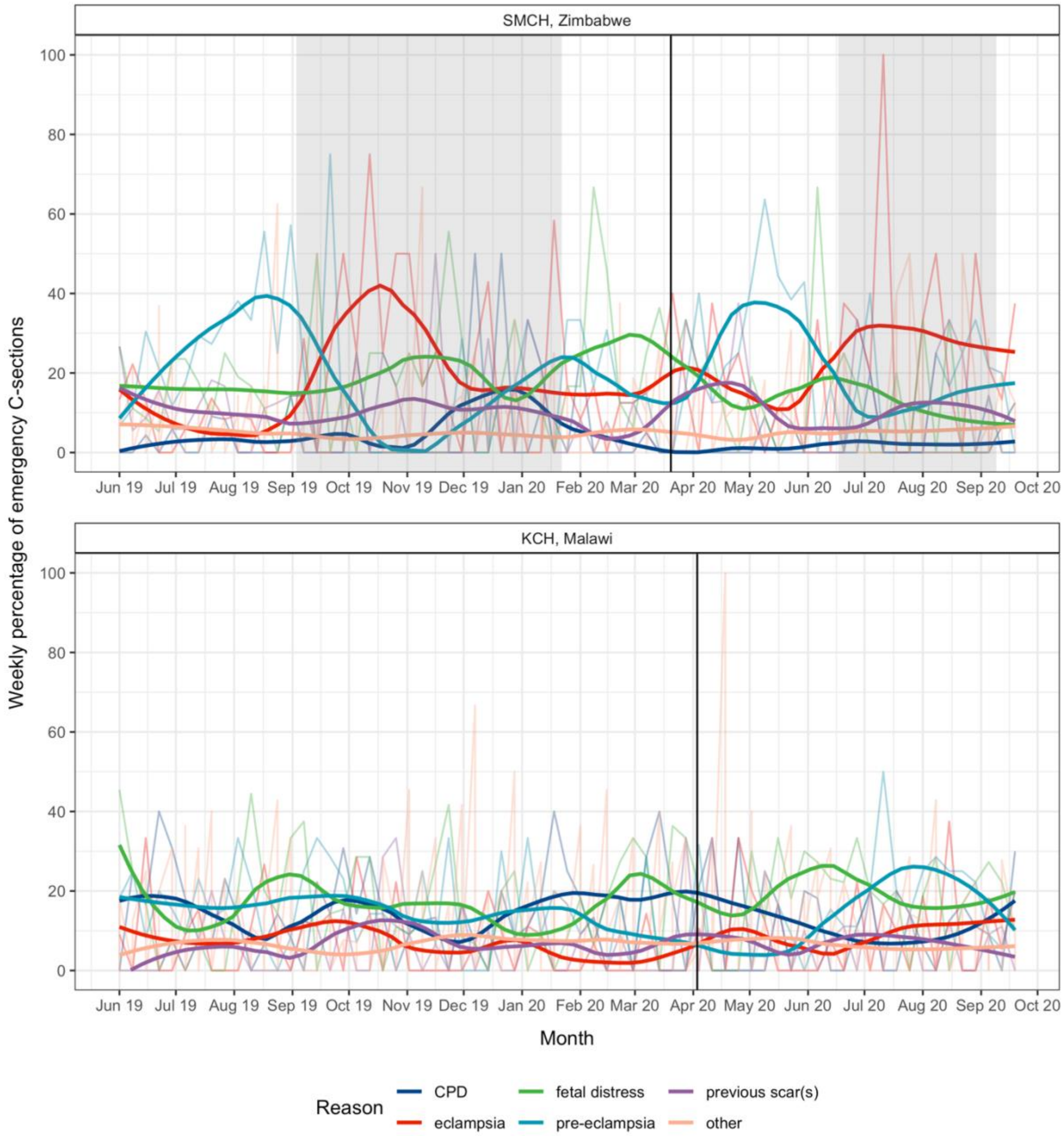

Figure A6.3.1: Trend in reason for emergency caesarean section per week

- Smoothed line: local regression (LOESS) model; 95% confidence interval not presented to avoid overplotting.
- Solid vertical line: first confirmed case of COVID-19 in each country.
- Shaded periods on SMCH, Zimbabwe panel: industrial action by doctors (3 September 2019 to 22 January 2020) and nurses (17 July 2020 to 9 September 2020).
- Counts based on all admission forms completed, irrespective of match status.
- *SMCH: Sally Mugabe Central Hospital; KCH: Kamuzu Central Hospital; CPD: cephalopelvic disproportion*

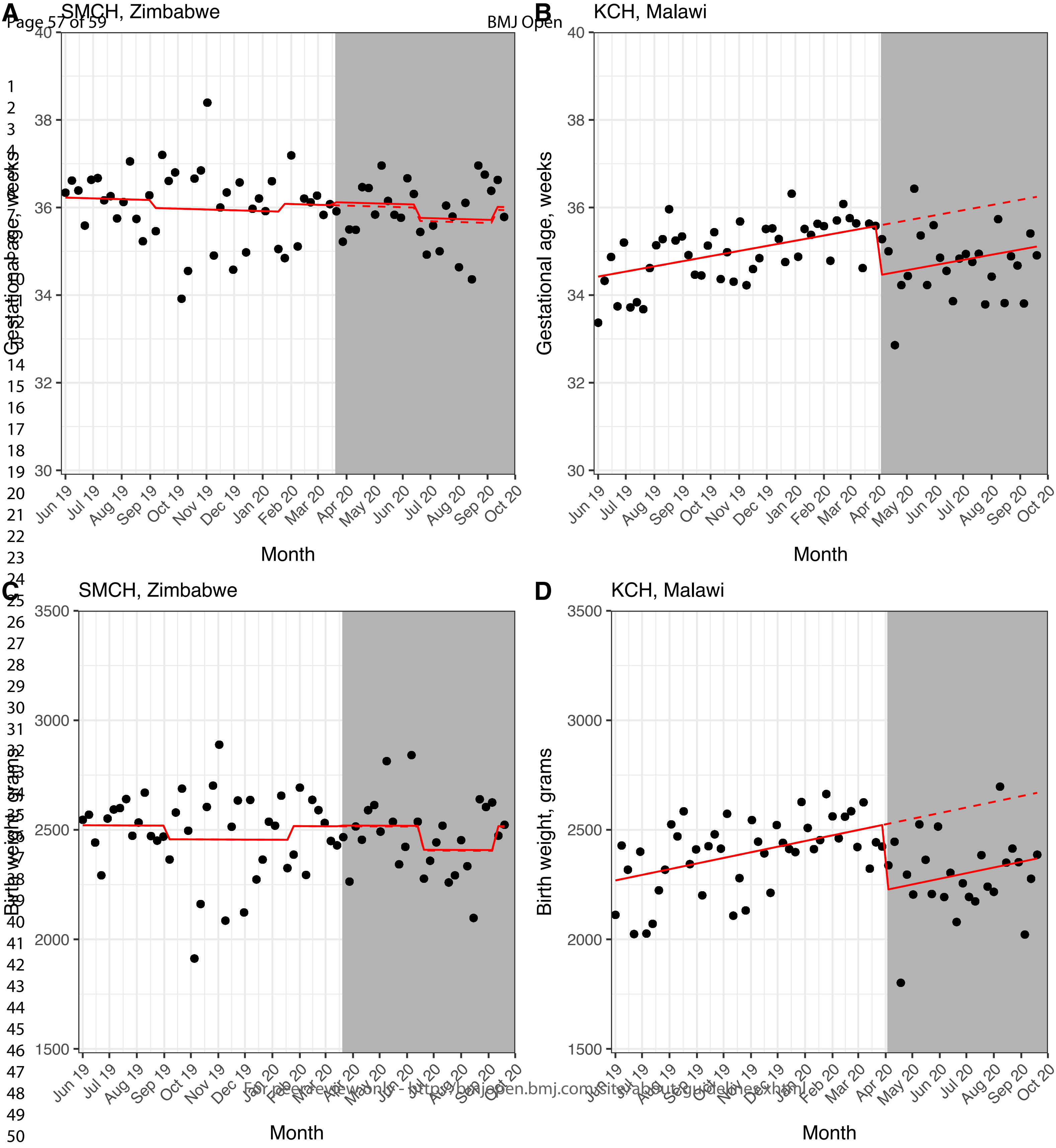
A
SMCH, Zimbabwe
Gestational age, weeks
Month
B
KCH, Malawi
Gestational age, weeks
Month
C
SMCH, Zimbabwe
Birth weight, grams
Month
D
KCH, Malawi
Birth weight, grams
Month
Jun 19
Jul 19
Aug 19
Sep 19
Oct 19
Nov 19
Dec 19
Jan 20
Feb 20
Mar 20
Apr 20
May 20
Jun 20
Jul 20
Aug 20
Sep 20
Oct 20

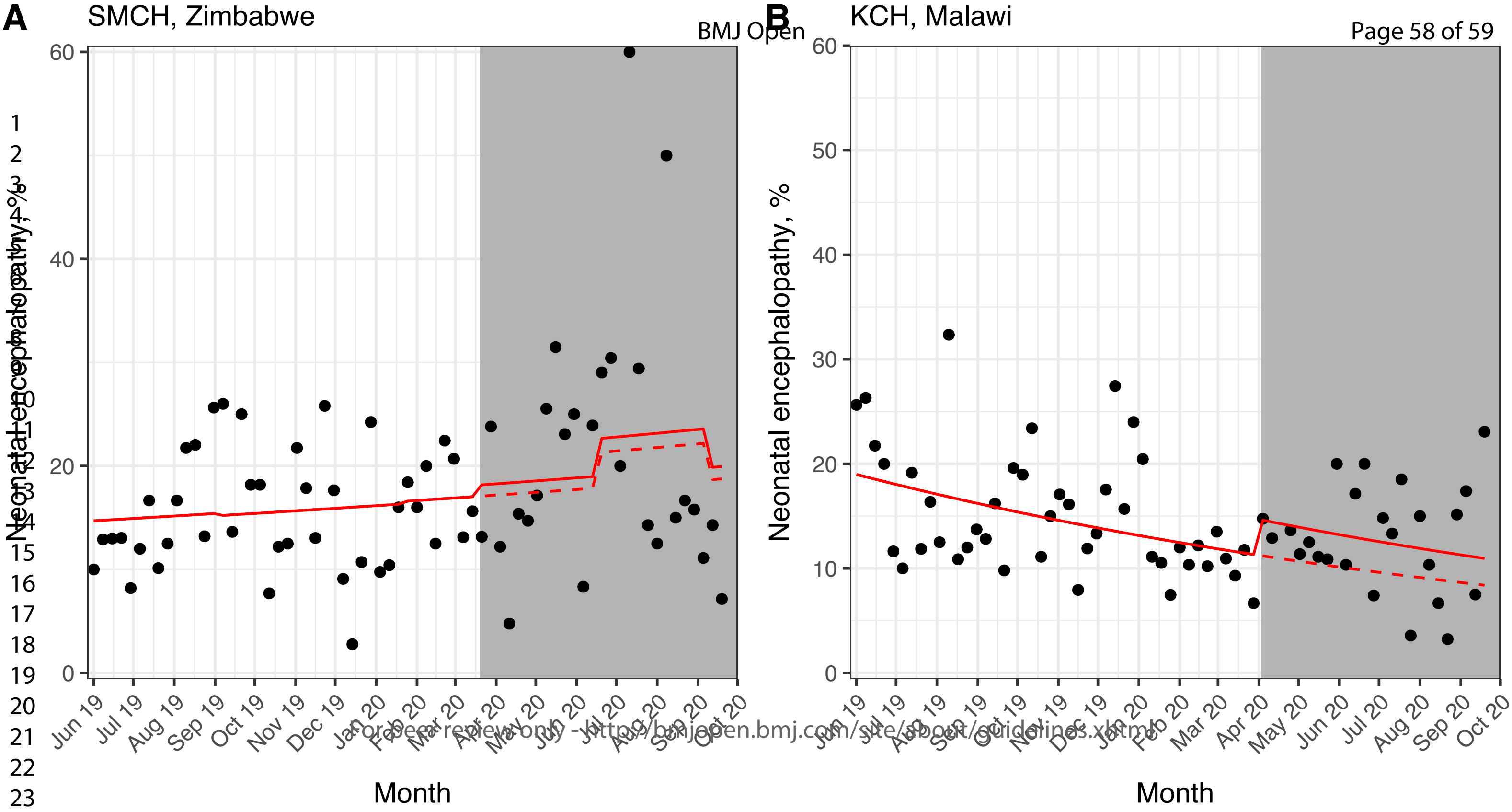
A
SMCH, Zimbabwe
Neonatal encephalopathy, %
0
20
40
60
Jun 19
Jul 19
Aug 19
Sep 19
Oct 19
Nov 19
Dec 19
Jan 20
Feb 20
Mar 20
Apr 20
May 20
Jun 20
Jul 20
Aug 20
Sep 20
Oct 20
Month
B
KCH, Malawi
Neonatal encephalopathy, %
0
10
20
30
40
50
60
Jun 19
Jul 19
Aug 19
Sep 19
Oct 19
Nov 19
Dec 19
Jan 20
Feb 20
Mar 20
Apr 20
May 20
Jun 20
Jul 20
Aug 20
Sep 20
Oct 20
Month

APPENDIX 1: STROBE CHECKLIST

	Item No.	Recommendation	Page No.
**Title and abstract**	1	(*a*) Indicate the study's design with a commonly used term in the title or the abstract	1-2
		(*b*) Provide in the abstract an informative and balanced summary of what was done and what was found	2
**Introduction**			
Background/rationale	2	Explain the scientific background and rationale for the investigation being reported	4-5
Objectives	3	State specific objectives, including any prespecified hypotheses	5
**Methods**			
Study design	4	Present key elements of study design early in the paper	9-10
Setting	5	Describe the setting, locations, and relevant dates, including periods of recruitment, exposure, follow-up, and data collection	6-8
Participants	6	(*a*) *Cohort study*—Give the eligibility criteria, and the sources and methods of selection of participants. Describe methods of follow-up<br>*Case-control study*—Give the eligibility criteria, and the sources and methods of case ascertainment and control selection. Give the rationale for the choice of cases and controls<br>*Cross-sectional study*—Give the eligibility criteria, and the sources and methods of selection of participants	7
		(*b*) *Cohort study*—For matched studies, give matching criteria and number of exposed and unexposed<br>*Case-control study*—For matched studies, give matching criteria and the number of controls per case	n/a
Variables	7	Clearly define all outcomes, exposures, predictors, potential confounders, and effect modifiers. Give diagnostic criteria, if applicable	8
Data sources/ measurement	8*	For each variable of interest, give sources of data and details of methods of assessment (measurement). Describe comparability of assessment methods if there is more than one group	7-8
Bias	9	Describe any efforts to address potential sources of bias	9-10
Study size	10	Explain how the study size was arrived at	7
Quantitative variables	11	Explain how quantitative variables were handled in the analyses. If applicable, describe which groupings were chosen and why	9-10

For peer review only

Statistical methods	12	(*a*) Describe all statistical methods, including those used to control for confounding	9-10
		(*b*) Describe any methods used to examine subgroups and interactions	9-10, Appendix 5
		(*c*) Explain how missing data were addressed	9-10, Appendix 4
		(*d*) *Cohort study*—If applicable, explain how loss to follow-up was addressed<br>*Case-control study*—If applicable, explain how matching of cases and controls was addressed<br>*Cross-sectional study*—If applicable, describe analytical methods taking account of sampling strategy	9
		(*e*) Describe any sensitivity analyses	9-10, Appendix 5
**Results**			
Participants	13*	(a) Report numbers of individuals at each stage of study—eg numbers potentially eligible, examined for eligibility, confirmed eligible, included in the study, completing follow-up, and analysed	12, Appendix 3
		(b) Give reasons for non-participation at each stage	9, Appendix 3
		(c) Consider use of a flow diagram	Appendix 3
Descriptive data	14*	(a) Give characteristics of study participants (eg demographic, clinical, social) and information on exposures and potential confounders	12-13, Appendix 5
		(b) Indicate number of participants with missing data for each variable of interest	Appendix 4
		(c) *Cohort study*—Summarise follow-up time (eg, average and total amount)	7
Outcome data	15*	*Cohort study*—Report numbers of outcome events or summary measures over time	12-15
		Case-control study—Report numbers in each exposure category, or summary measures of exposure	n/a
		Cross-sectional study—Report numbers of outcome events or summary measures	n/a
Main results	16	(*a*) Give unadjusted estimates and, if applicable, confounder-adjusted estimates and their precision (eg, 95% confidence interval). Make clear which confounders were adjusted for and why they were included	12-15
		(*b*) Report category boundaries when continuous variables were categorized	12-15
		(*c*) If relevant, consider translating estimates of relative risk into absolute risk for a meaningful time period	12-15

For peer review only

Other analyses	17	Report other analyses done—eg analyses of subgroups and interactions, and sensitivity analyses	Appendix 5, Appendix 6
**Discussion**			
Key results	18	Summarise key results with reference to study objectives	16
Limitations	19	Discuss limitations of the study, taking into account sources of potential bias or imprecision. Discuss both direction and magnitude of any potential bias	18-19
Interpretation	20	Give a cautious overall interpretation of results considering objectives, limitations, multiplicity of analyses, results from similar studies, and other relevant evidence	16-19
Generalisability	21	Discuss the generalisability (external validity) of the study results	16-19
**Other information**			
Funding	22	Give the source of funding and the role of the funders for the present study and, if applicable, for the original study on which the present article is based	26

Adapted from: von Elm E, Altman DG, Egger M, Pocock SJ, Gøtzsche PC, et al. (2007) The Strengthening the Reporting of Observational Studies in Epidemiology (STROBE) Statement: Guidelines for Reporting Observational Studies. PLOS Medicine 4(10): e296. https://doi.org/10.1371/journal.pmed.0040296

For peer review only
